# Identification of a novel bovine enterovirus possessing highly divergent amino acid sequences in capsid protein

**DOI:** 10.1186/s12866-016-0923-0

**Published:** 2017-01-17

**Authors:** Shinobu Tsuchiaka, Sayed Samim Rahpaya, Konosuke Otomaru, Hiroshi Aoki, Mai Kishimoto, Yuki Naoi, Tsutomu Omatsu, Kaori Sano, Sachiko Okazaki-Terashima, Yukie Katayama, Mami Oba, Makoto Nagai, Tetsuya Mizutani

**Affiliations:** 1 The United Graduate School of Veterinary Sciences, Gifu University, 1-1 Yanagito, Gifu-shi, Gifu 501-1193 Japan; 2 Research and Education Center for Prevention of Global Infectious Disease of Animals, Tokyo University of Agriculture and Technology, 3-5-8 Saiwai-cho, Fuchu-shi, Tokyo 183-8509 Japan; 3 Joint Faculty of Veterinary Medicine, Kagoshima University, 1-21-24 Korimoto, Kagoshima-shi, Kagoshima 890-0065 Japan; 4 Faculty of Veterinary Science, Nippon Veterinary and Life Science University, 1-7-1 Kyonan-cho, Musashino-shi, Tokyo 180-8602 Japan; 5 Faculty of Bioresources and Environmental Sciences, Ishikawa prefectural University, 1-308, Suematsu, Nonoichi-shi, Ishikawa 921-8836 Japan

**Keywords:** Bovine enterovirus, Deep sequencing, Phylogenetic analysis

## Abstract

**Background:**

Bovine enterovirus (BEV) belongs to the species *Enterovirus E* or *F*, genus *Enterovirus* and family *Picornaviridae*. Although numerous studies have identified BEVs in the feces of cattle with diarrhea, the pathogenicity of BEVs remains unclear. Previously, we reported the detection of novel kobu-like virus in calf feces, by metagenomics analysis. In the present study, we identified a novel BEV in diarrheal feces collected for that survey. Complete genome sequences were determined by deep sequencing in feces. Secondary RNA structure analysis of the 5′ untranslated region (UTR), phylogenetic tree construction and pairwise identity analysis were conducted.

**Results:**

The complete genome sequences of BEV were genetically distant from other EVs and the VP1 coding region contained novel and unique amino acid sequences. We named this strain as BEV AN12/*Bos taurus*/JPN/2014 (referred to as BEV-AN12). According to genome analysis, the genome length of this virus is 7414 nucleotides excluding the poly (A) tail and its genome consists of a 5′UTR, open reading frame encoding a single polyprotein, and 3′UTR. The results of secondary RNA structure analysis showed that in the 5′UTR, BEV-AN12 had an additional clover leaf structure and small stem loop structure, similarly to other BEVs. In pairwise identity analysis, BEV-AN12 showed high amino acid (aa) identities to *Enterovirus F* in the polyprotein, P2 and P3 regions (aa identity ≥82.4%). Therefore, BEV-AN12 is closely related to *Enterovirus F*. However, aa sequences in the capsid protein regions, particularly the VP1 encoding region, showed significantly low aa identity to other viruses in genus *Enterovirus* (VP1 aa identity ≤58.6%). In addition, BEV-AN12 branched separately from *Enterovirus E* and *F* in phylogenetic trees based on the aa sequences of P1 and VP1, although it clustered with *Enterovirus F* in trees based on sequences in the P2 and P3 genome region.

**Conclusions:**

We identified novel BEV possessing highly divergent aa sequences in the VP1 coding region in Japan. According to species definition, we proposed naming this strain as “Enterovirus K”, which is a novel species within genus *Enterovirus*. Further genomic studies are needed to understand the pathogenicity of BEVs.

**Electronic supplementary material:**

The online version of this article (doi:10.1186/s12866-016-0923-0) contains supplementary material, which is available to authorized users.

## Background

Bovine enterovirus (BEV) is a single positive-stranded RNA virus belonging to the genus *Enterovirus* within family *Picornaviridae*. The viral particle is composed of a small, non-enveloped and icosahedral virion and 7.5 k-base genome containing a single open reading frame (ORF) flanked by untranslated regions (UTRs) at the 5′ and 3′ ends. The ORF encodes a single long polyprotein containing structural proteins (VP1, VP2, VP3 and VP4 encoded in P1) and non-structural proteins (2A, 2B and 2C encoded in P2 as well as 3A, 3B, 3C and 3D encoded in P3) [1, 2].

Genus *Enterovirus* is divided into 12 species defined as *Enterovirus A*–*H* and *J* (EV-A, B, C, D, E, F, G, H and J) and *Rhinovirus A*–*C* (RV-A, B and C) [2]. BEVs belong to EV-E and EV-F (formerly known as BEV-A and BEV-B, respectively) and can be distinguished from other EVs by the unique secondary structure of their RNA genome: a 5′-cloverleaf and internal ribosome entry site (IRES) linked by additional nucleotide sequences at the 5′UTR [3–5]. Since the isolation of BEVs from cattle in the late 1950s [6–8], studies worldwide have detected BEVs not only in cattle but also in other animal species including possums, bottlenose dolphins, camels and alpacas [8–12]. Although BEVs have been classified based on virus antigenicity determined by cross neutralization testing [13–16], the genotype based on the capsid protein (particularly in VP1) amino acid sequences are also used to classify BEVs [4, 10–12, 17, 18]. BEVs are classified into 4 sero-/genotypes and 6 sero-/genotypes in EV-E (E1, E2, E3 and E4) and EV-F (F1, F2, F3, F4, F5 and F6), respectively.

Although most EVs cause only mild symptoms, including hand-foot-and-mouth disease, herpangina, pleurodynia and rashes [19, 20], some members belonging to the genus *Enterovirus* can cause severe diseases. The most well known pathogen is poliovirus affecting humans. Poliovirus and some of other EVs, including coxsackie virus and echovirus, can invade the central nervous system causing neurological diseases, including aseptic meningitis, encephalitis and ataxia [21, 22]. In other animals, although porcine teschovirus, formerly classified as porcine enterovirus, can cause a neurological disorder known as Teschen/Talfan disease [23], the pathogenicity of EVs infecting animals are still unclear. In case of cattle, foot-and-mouth disease virus belonging to the genus *Aphthovirus* of the family *Picornaviridae* can cause vesicular diseases leading to a serious economic impact for farmers [24]; the pathogenicity of viruses belonging to the genus *Enterorovirus* is still unclear. Several reports have claimed that BEVs can cause diarrhea, respiratory diseases, reproductive diseases and infertility in cattle [25–27]; however, BEVs have also been widely detected in asymptomatic cattle and their environment, and experimental infection trials of BEV have failed to produce clinical signs [28–30]. Therefore, whether BEV infection is clinically important remains unclear.

It is widely known that most viruses belonging to genus *Enterovirus* utilize “canyon” as their binding site to cells surface receptors, which is formed by outer capsid proteins including VP1, VP2 and VP3 [31]. Several studies of other enteroviruses revealed that sequences of the VP1 coding region are responsible for the phenotype of viruses; some amino acid substitutions in this region altered the pathogenicity and cell tropism of the viruses [32–34]. Although the cell surface receptor to BEV has not been identified, it is likely that the capsid proteins, including VP1, may be responsible for the phenotype of BEVs, as their capsid proteins also form a “canyon” on the outer side of the virion, and a strain isolated from cattle with severe symptoms contained specific amino acid substitutions in the capsid regions [27, 35]. To elucidate the determinants of BEV virulence in hosts, genomic information of BEVs must be determined.

Recently, deep sequencing techniques using high-throughput sequencers have been used to evaluate virome including novel viruses in clinical samples without viral isolation to determine total genomic information within samples [36, 37]. We previously identified novel viruses infecting the intestinal tracts of livestock using high-throughput sequencers to study enterovirus, picornavirus and astrovirus in the feces of goat, swine and cattle, respectively [38–41].

Previously, we reported the detection of novel kobu-like virus in Japanese Black cattle, using feces of calf, by metagenomics analysis. In the present study, we identified a novel BEV in feces collected for that survey [42]. To characterize the genomic features of this virus, complete genome sequences were determined and phylogenetic trees were constructed. In addition, secondary RNA structures in the 5′UTR and pairwise identity were analyzed.

## Methods

### Fecal sample and virus isolation

Previously, we reported the detection of a novel kobu-like virus in Japanese black cattle by deep sequencing method [42]. During the metagenomics surveillance, nucleotide sequences with high similarity to BEVs were identified in feces collected from a calf with diarrhea. This feces was collected from a 1-month-old calf with diarrhea in Kagoshima prefecture (Kagoshima sample) in 2014. No other clinical sign was observed except diarrhea. Feces was collected directly from the rectum on the onset day. One gram feces was diluted with 9 mL PBS (−) to prepare a 10% fecal suspension and centrifuged at 10,000 × g for 10 min. The supernatant was collected and stored at −80 °C before RNA extraction and virus isolation.

The supernatant of the Kagoshima sample was subjected to virus isolation. The fecal supernatant was filtered through a 0.45-μm pore size membrane and treated with 10 μg/mL acetylated trypsin (Sigma-Aldrich, St. Louis, MO, USA) for 60 min at room temperature before virus isolation. Treated samples were inoculated into Mardin-Darby bovine kidney cells. Blind passage was subsequently conducted three times. Minimum Essential Medium was used as negative control (Sigma-Aldrich).

### Isolated BEV strains

In this study, three BEVs isolated in Japan, BEV IS1/*Bos taurus*/JPN/1990 (BEV-IS1) and IS2/*Bos taurus*/JPN/1990 (BEV-IS2), were additionally sequenced and analyzed. These viruses were isolated from a fecal sample collected from one cow at the same time in 1990 in the Ishikawa prefecture (The clinical features of cattle infected with BEV-IS1 and IS2 have not been recorded). In addition, BEV Ho12/*Bos taurus*/JPN/2009 (BEV-Ho12) was isolated from diarrheic feces collected in Hokkaido in 2009 by as described above [39].

### RNA extraction, cDNA library construction and whole genome sequencing

Total RNA was extracted from 0.25-mL supernatants of isolated viruses and 10% fecal samples using TRIzol LS Reagent (Life Technologies, Carlsbad, CA, USA). RNA samples were normalized to 10–100 ng of RNA per reaction, using a Qubit_2.0 Fluorometer (Invitrogen, Carlsbad, CA, USA). The cDNA library of sample RNA was constructed using the NEBNext Ultra RNA Library Prep Kit for Illumina version 2.0 (New England Biolabs, Ipswich, MA, USA) as described previously [40] and sequenced using MiSeq (Illumina, San Diego, CA, USA) with the MiSeq reagent kit V2 (300 cycles) (Illumina). Briefly, all reads were generated as 151 paired end reads. Each sample was multiplexed with other 23 samples prepared from diarrheal feces of other calves (data not shown). 5′-Full RACE Core Set (TaKaRa Bio, Shiga, Japan) and 3′Full RACE Core Set (TaKaRa Bio) were used to complement virus sequences of the 5′ end and 3′ end, respectively.

### Analysis of genome sequences

All nucleotide sequences determined by Miseq (referred to as “reads”) were converted to FASTAQ format on MiSeq reporter V2.3 and subsequently analyzed using CLC Genomics Workbench 6.0 (CLC bio, Cambridge, MA, USA). Briefly, the ends of all reads were trimmed to remove adaptor sequences located at both ends of each read. Trimmed reads were assembled into contigs using a de novo assembly algorithm. Contigs generated by de novo assembly algorithm were analyzed using BlastN.

Hypothetical polyprotein cleavage sites of the viruses were predicted by multiple alignments with other BEVs and confirmed by the NetPicoRNA [43]. Nucleotide (nt) sequences or amino acid (aa) sequences were aligned using ClustalW. Phylogenetic trees were constructed by maximum likelihood (ML) methods on MEGA5.2.2 [44]. The mtREV24 + G + F model (5′UTR), rtREV + F model (3′UTR), rtREV + G + F model (P1), rtREV + G + I (P2 and P3), and WAG + G + I (VP1) were employed as evolutionary models for ML method. Pairwise identity was analyzed on CLC Genomics Workbench and the secondary RNA structure of the 5′UTR was predicted by Mfold [45].

### VP1 genome sequencing

RT-PCR was performed by using PrimeScript One Step RT-PCR Kit Ver.2 (TaKaRa Bio) to confirm the sequences of the contigs obtained from the Kagoshima sample. Three primer sets were designed based on the contig sequences of this sample. Primer sequences are given in Additional file 1: Table S1. PCR products were sequenced using a 3130xl Genetic analyzer (Applied Biosystems, Foster City, CA, USA).

### Detection of other pathogens causing diarrhea

To confirm the presence of other pathogens in the Kagoshima sample, detection of agents causing diarrhea using our real-time PCR system, referred to as “Dembo-PCR,” was performed [46]. This system can identify 19 species of pathogens, including virus, bacteria and protozoa. Briefly, viral DNA and RNA were extracted by high pure viral nucleic acid extraction kit (Roche Diagnostics GmbH, Mannheim, Germany) and bacteria and protozoa DNA were extracted by QIAamp Fast DNA stool mini kit (QIAGEN, Hilden, Germany). Nucleic acids extracted by each kit were subjected to Dembo-PCR, according to a previous report [46].

## Results

### Virus isolation and determination of viral genome sequences

Although virus isolation using supernatants of the Kagoshima feces was repeated three times, no cytopathic effect could be detected. Therefore, RNA extracted from the Kagoshima sample collected in 2014 and virus stocks of BEV-IS1, BEV-IS2 and BEV-Ho12 were subjected to deep sequencing. The Kagoshima sample was sequenced twice, and all reads obtained from the two runs were used to generate contigs (the first and second deep sequencing yielded 1,304,032 and 929,976 reads, respectively). The results of BlastN analysis revealed that bovine enterovirus F, group A rotavirus (RVA), bovine kobu-like virus and bovine picornavirus were identified with E value = 0. However, RVA was not detected in feces by Dembo-PCR.

The total BEV read counts (percentages indicate BEV reads per total reads of the first and second deep sequencing) of the Kagoshima sample were 1202 reads (0.05%), and an approximately 7400 nt contig was obtained from the integrated result with a 24.24 average sequence read depth (maximum read depth was 46). The complete genome was determined using 5′ and 3′ end RACE methods. Because amino acid sequences of VP1 were not similar to those of other enteroviruses by homology analysis as described below, the VP1 genome sequence was confirmed by directly sequencing the PCR product. As a result, sequences obtained from direct sequencing agreed with the results of deep sequencing. The genome length of BEV from the Kagoshima sample was 7414 nt, excluding the poly (A) tail. We named this BEV as BEV AN12/*Bos taurus*/JPN/2014 (BEV-AN12).

Viral genomes of isolated viruses including complete ORFs were also determined. The genome lengths of BEV-IS1, BEV-IS2 and BEV-Ho12 were 7413 nt (P1: 2517 nt, P2: 1737 nt, and P3: 2271 nt), 7394 nt (P1: 2496 nt, P2: 1734 nt, and P3: 2271 nt), and 7350 nt (P1: 2496 nt, P2: 1734 nt, and P3: 2271 nt), respectively. The sequences of BEV-AN12, BEV-Ho12, BEV-IS1 and BEV-IS2 were deposited in the DDBJ/EMBL/GenBank database under the accession numbers LC038188, LC150008, LC150009 and LC150010, respectively.

### Pairwise identity and genome analysis

Table 1 shows the pairwise aa (polyprotein, 2C + 3CD, P1-P3, VP1-VP4 and 3D) or nt (5′UTR and 3′UTR) identity of BEV-AN12 to representative strains of each species belonging to BEVs and other Japanese BEVs. Deduced aa sequences encoding polyprotein, 2C + 3CD, P1, P2, P3, 3D and four capsid proteins (encoding VP4, VP2, VP3 and VP1) were compared to each EV-E and F. BEV-AN12 possessed showed identity to EV-Fs in polyprotein, 2C + 3CD, P2, P3 and 3D than to those of EV-Es. However, low aa identity (aa identity <70%) was observed in P1 to EV-Es and EV-Fs. Particularly, the VP1 region of BEV-AN12 encoded in P1 showed a significantly low aa identity to other BEVs (54.7% ≤ aa identity ≤ 58.6%). As a result of multiple alignment analysis, several motifs conserved among the genomes of genus *Enterovirus* were detected in the genome of BEV-AN12. In particular, the [PS] ALXAAXETG motif in VP1, GXCG motif in 2A, GXXGXGKS motif for NTP-binding in 2C, GXCG motif forming part of the catalytic active site in 3C, and KDE [LI] R in 3D were identified [47–50]. However, the putative cleavage site at the junction of VP3/VP1 was a glutamine/serine for BEV-AN12. In addition, a 6-aa insertion in the 2A region was identified at position 835–840 aa (PLRTTG) in the BEV-AN12 genome. Multiple alignment using aa sequences encoding polyprotein is supplemented as Additional file 2: Table S2.

**Table 1:** Pairwise nucleotide (5′UTR and 3′UTR) and amino acid (polyprotein, 2C + 3CD, P1-P3, 3D and VP1-4) identity (%) of BEV-AN12 to Japanese BEVs, EV-Es and EV-Fs

	BEV AN12/*Bos Taurus*/JPN/2014 (LC038188)										
	Bovine enterovirus in Japan			EV-E1	EV-E2	EV-E3	EV-E4	EV-F1	EV-F2	EV-F3	EV-F4
	IS1/*Bos taurus*/JPN/1990(LC150009)	IS2/*Bos taurus*/JPN/1990(LC150010)	Ho12/*Bos taurus*/JPN/2009 (﻿LC150008)	LC-R4 (DQ092769)	K2577 (AF123432)	HY12 (KF748290)	PAK-NIH-21E5 (AFK92921)	BEV-261 (NC_021220)	3A (AY508697)	PS87/Belfast (DQ092794)	Possum enterovirus W6 (AY462107)
5′UTR	76.1	79.8	80.8	75.8	74.2	71.1	N/A	81.8	80.4	81.3	78.0
Polyprotein	73.6	86.6	86.2	73.2	73.6	72.7	N/A	85.0	84.6	85.6	82.4
2C + 3CD	80.9	98.8	98.6	80.7	80.9	80.2	N/A	98.1	98.1	93.6	97.9
P1	65.8	68.7	68.3	65.1	66.0	64.5	N/A	68.2	67.2	67.6	67.1
VP4	75.4	85.5	85.5	75.4	79.7	75.4	N/A	82.6	85.5	85.5	82.6
VP2	74.2	72.6	73.0	72.2	72.6	73.4	N/A	72.6	71.4	71.0	70.2
VP3	65.0	71.6	71.2	65.8	65.8	64.6	N/A	70.0	70.0	70.8	72.8
VP1	56.9	57.9	56.8	54.7	56.5	56.2	54.7	56.1	58.6	55.0	56.8
P2	77.8	96.4	95.7	78.0	77.6	77.3	N/A	92.3	91.6	94.9	89.7
P3	79.0	98.8	98.5	78.6	79.1	78.3	N/A	98.0	98.3	98.3	93.7
3D	84.0	99.1	98.7	83.7	83.7	83.5	N/A	98.7	98.9	98.9	95.0
3′UTR	71.6	86.1	N/A	71.6	71.6	73.0	N/A	84.7	86.1	86.1	81.9

N/A: Sequences are not available

### Secondary RNA structure of 5′UTR

**Fig. 1:**
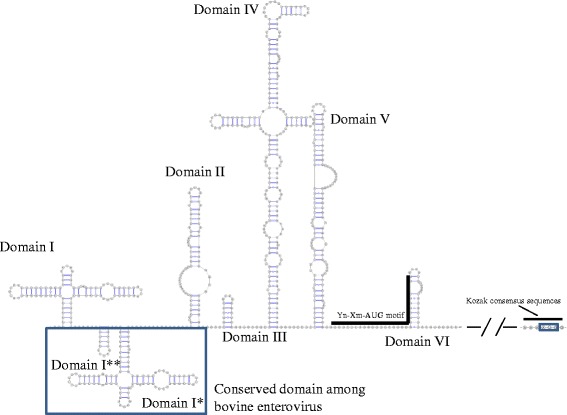
Secondary RNA structure of 5′UTR of BEV-AN12. Putative secondary RNA structure of 5′UTR of BEV-AN12 was predicted by Mfold. Domains I, I*, I**, II, III, IV, V and VI domain were predicted. Domains I* and I** conserved among BEVs are shown with a bold line. Yn-Xm-AUG motif conserved in domain VI is indicated by a bold line. Kozak consensus sequences with start codon are also indicated by a bold line

The putative secondary 5′UTR RNA structure of BEV-AN12 is shown in Fig. 1. Zell et al. reported that the 5′UTR of BEVs form additional cloverleaf structures and small stem loops between the 5′-cloverleaf structure and IRES, which clearly distinguish BEVs from other EVs [3]. Similarly, our analysis revealed that all Japanese BEVs had BEV-specific structures (domains I* and I**). Domains II, III, IV, V and VI, which are the main domains of type 1 IRES directing cap-independent translation [51], were also observed in all Japanese BEVs.

### Phylogenetic analysis

**Fig. 2:**
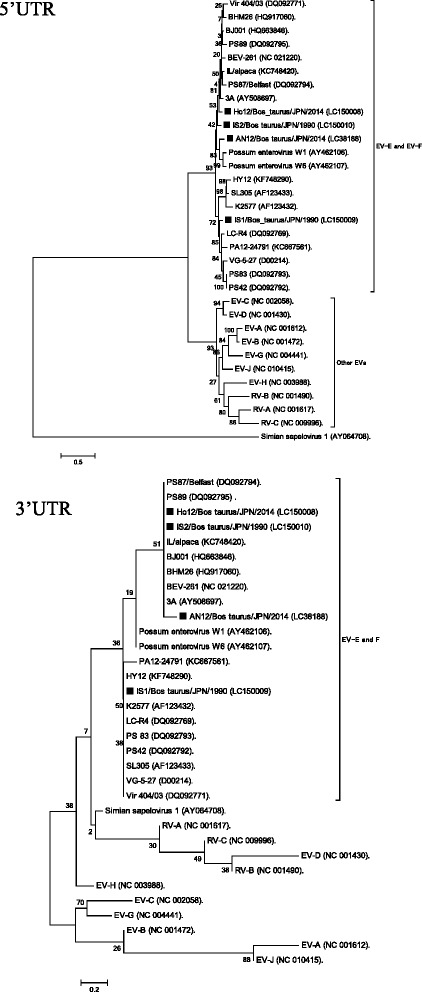
Phylogenetic trees using nucleic acid sequences of 5′UTR and 3′UTR. Phylogenetic trees were constructed using nucleic acid sequences of EV-A to EV-J and Japanese BEVs based on the maximum likelihood method in MEGA5.22 with bootstrap values calculated for 1000 replicates. Scale bar indicates nucleotide substitutions per site. BEVs in Japanese were indicated as ■. *Simian sapelovirus* (AY064708) was used as outgroup

**Fig. 3:**
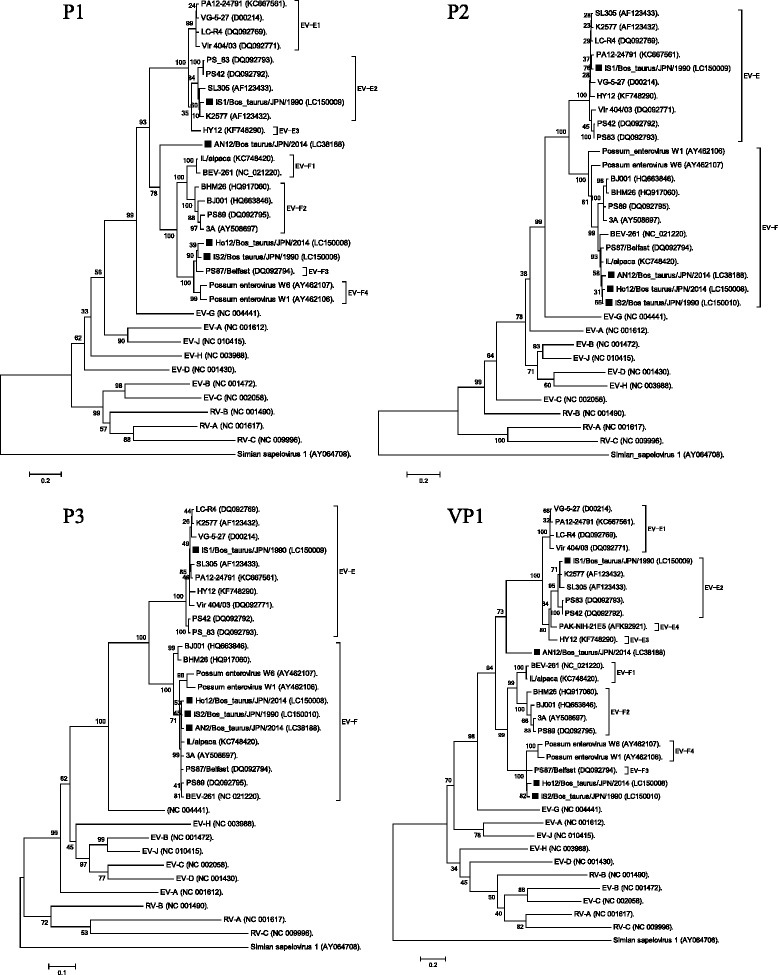
Phylogenetic trees using amino acid sequences of P1, P2 and P3 and VP1 proteins. Phylogenetic trees were constructed using amino acid sequences of EV-A to EV-J and Japanese BEVs based on the maximum likelihood method in MEGA5.22 with bootstrap values calculated for 1000 replicates. Scale bar indicates amino acid substitution per site. BEVs in Japanese were indicated as ■. *Simian sapelovirus* (AY064708) was used as outgroup

Phylogenetic trees based on the nt sequences of 5′UTR and 3′UTR and aa sequences of P1, P2, P3 and VP1 are shown in Fig. 2 and Fig. 3, respectively. Phylogenetic analysis showed that all Japanese strains of BEV including BEV-AN12 formed a cluster with other BEVs in all six trees. However, BEV-AN12 was completely separated from other BEVs in the P1 and VP1 trees.

### Detection of agents causing diarrhea by Dembo-PCR

Dembo-PCR was performed to identify agents causing diarrhea in calf. According to the results, only BEV was detected by this test. Genome of other pathogens was not identified in the fecal sample of Kagoshima.

## Discussion

According to the species demarcation criteria for the genus *Enterovirus* defined by the International Committee on Taxonomy of Viruses, members of a species in the genus *Enterovirus* should share high aa identity (aa >70% in the polyprotein, aa >60% in P1 and >80% aa identity in 2C + 3CD) and compatibility in processing, replication and encapsidation [2]. In addition, EV-E and F can be distinguished from other EVs because of their unique secondary RNA structures in the 5′UTR region (domains I* and I**) [4]. Our genome analysis revealed that BEV-AN12 shared aa sequences and protease cleavage site positions with EV-Fs. In addition, BEV-AN12 contained domains I* and II* in the 5′UTR similarly to other BEVs. Therefore, BEV-AN12 is closely related to EV-Fs. However, pairwise identity analysis revealed that aa sequences in the VP1 region of the BEV-AN12 genome had significantly low identities to other BEVs strains (VP1 aa identity ≤58.6%). Furthermore, BEV-AN12 did not cluster with any other EV-E and EV-F in the VP1 phylogenetic tree, although its P2 and P3 regions were closely related to EV-F. The percentage of aa identity of VP1 is commonly utilized for species and sero-/genotype definition (range from 50 to 55% for heterologous species, 70 to 85 % for heterologous sero-/genotypes/homologous species, and greater than 90 % for homologous sero-/genotypes) [4]. According to the classification definition, our results indicate that BEV-AN12 is taxonomically distant from previously reported BEVs. Therefore, we named this strain as “*Enterovirus K*”, which is a novel species within the genus *Enterovirus*. Other Japanese BEVs were classified as typical BEVs (BEV-IS1: EV-E2, BEV-IS2 and BEV-Ho12: EV-F4).

Our recombination analysis could not reveal the source of mutation (data not shown), although several reports suggested that recombinant viruses belonging to the genus *Enterovirus* were generated by intra/interspecies transmission [52, 53]. Point mutations in the viral genome are common among picornaviruses because their polymerase lacks the proofreading ability and fidelity of amplification [54–56]. In addition, VP1 is a capsid protein, which likely influences host immunity in infected animals [35]. Therefore, the accumulation of mutations in the viral genome and subsequent selection by immunity in infected hosts may result in the generation of novel species.

Although the complete ORF and complete or partial UTRs sequences of four Japanese BEVs were determined, the virus could not be isolated from one diarrheal feces of a calf (BEV-AN12). BEV-AN12 has mutations in VP1 and 2A, which are involved in the formation of the capsid protein-host receptor binding site and cell proliferation, respectively [35, 49]. Although critical motifs for their function including [PS] ALXAXETG and GXCG were identified in the BEV-AN12 genome, a short insertion (6 aa) in the 2A protein region and non-synonymous substitution at the junction of VP3/VP1 were observed in the BEV-AN12 genome. Because these mutations may show alter receptor binding or virus replication, further crystal structure analysis of virions should be conducted.

The VP1 proteins in viruses belonging to the genus *Enterovirus* are widely known to components that form the receptor binding site (this site is referred to as the “canyon”) together with VP2 and VP3 [31]. Reverse genetic analysis of other enteroviruses revealed that amino acid substitution in the VP1 region was responsible for the virus phenotype, such as pathogenicity and cell tropism [32–34]. Mutations in capsid protein genes may influence the structure of the “canyon” and receptor-binding capacity. BEVs also form a “canyon” on the outer side of the virion, although the cell surface receptor for BEVs is unknown. Therefore, BEVs may also have specific determinants for their phenotypes based on the aa sequences in the capsid protein encoding region. We also tried to investigate the prevalence of BEV-AN12, using VP1 specific primers, (Additional file 1: Table S1) in 38 diarrheal and 28 non-diarrheal feces samples collected from calves in Kagoshima prefecture during 2014–2015; feces from only one calf was positive, as revealed in the results. Therefore, we could not analyze the relationship between BEV-AN12 and its pathogenicity. According to the results of deep sequencing, we identified bovine picornavirus- and bovine kobu-like virus in the Kagoshima sample. There are reports suggesting that bovine picornavirus and bovine kobuvirus are associated with diarrhea [39, 57]. However, the pathogenicity of these viruses is still unknown. There is a possibility that all viruses can cause diarrhea. To clarify the determinants of the pathogenicity of BEVs, experimental infection based on reverse genetic analysis is necessary.

## Conclusions

The present study identified novel BEV possessing highly divergent aa sequences in the VP1 coding region in Japan. We name this strain as “*Enterovirus K*”, which is a novel species within the genus *Enterovirus*. To exclude the pathogenicity of BEVs, further genomic information must be accumulated.

## Additional files

Additional file 1: Table S1. Primers information for VP1 sequencing. (PDF 84 kb)
Additional file 2: Table S2. Multiple alignments result using amino acid sequences of polyprotein. (PDF 458 kb)

## Abbreviations

aa: Amino acid
BEV: Bovine enterovirus
EV: Enterovirus
IRES: Internal ribosome entry site
nt: Nucleotide
ORF: Open reading frame
UTR: Untranslated region
