# Supplementary material for: Identification of a novel bovine enterovirus possessing highly divergent amino acid sequences in capsid protein
Source: BMC Microbiol. 2017 Jan 17;17:18. doi: 10.1186/s12866-016-0923-0 (PMC5240211; doi:10.1186/s12866-016-0923-0)
Supplement: Additional file 1: Table S1. — Primers information for VP1 sequencing. (PDF 84 kb) [file 12866_2016_923_MOESM1_ESM.pdf]

Additional file 1: Table S1. Primers information for VP1 sequencing

Primer set	Primer sequences (5'-/-3')	Position*	Predicted amplicom size (bp)
Set 1	GTAGCGTTTGTAGCAGCTCA	2,412..2,431	559
	CACTCTGCCACTGGTAAGTATC	2,949..2,970	
Set 2	TAATCAACTTTGGGGAGTTTGTC	2,761..2,783	523
	GTCACAGCGGTGTAAGAATTG	3,263..3,283	
Set 3	CAACCCATCTCTGCTCTCAA	2,975..2,994	438
	CAGTCTGCCAGGTGTCTG	3,395..3,412	

* The position was based on BEV/AN12/*Bos taurus*/JPN/2014 (GenBank accession No. LC038188)
