# Supplementary material for: Identification of a novel bovine enterovirus possessing highly divergent amino acid sequences in capsid protein
Source: BMC Microbiol. 2017 Jan 17;17:18. doi: 10.1186/s12866-016-0923-0 (PMC5240211; doi:10.1186/s12866-016-0923-0)
Supplement: Additional file 2: Table S2. — Multiple alignments result using amino acid sequences of polyprotein. (PDF 458 kb) [file 12866_2016_923_MOESM2_ESM.pdf]

Additional file 2: Table S2. Multiple alignments result using amino acid sequences of polyprotein

P1 protein

Strain	VP4	VP2
AN12/Bos taurus/JPN/2014	MGGQFSKNTAGSHTTGTYAAGGSTIHYTNINYYENAASSSLNKQDLTQDPTKFTQPVVDVIKESAVPLK	SPSAEACGYSDRVAQLTLGNSTITTQEAANI
EV-E1 PA12-24791 (KC667561)	MGAQLSRNTAGSHTTGTYATGGSTINYNNINYYSHAASAAQNKQDFTQDPSKFAQPIADVIKETAVPLK	SPSAEACGYSDRVAQLTLGNSTITTQEAANI
EV-E1 VG-5-27 (D00214)	MGAQLSRNTAGSHTTGTYATGGSTINYNNINYYSHAASAAQNKQDFTQDPSKFTQPIADVIKETAVPLK	SPSAEACGYSDRVAQLTLGNSTITTQEAANI
EV-E1 LC-R4 (DQ092769)	MGAQMSRNTAGSHTTGNYATGGSTINYNNINYYSHAASDAQNKQDFTQDPSKFTQPIADVIKETAVPLK	SPSAEACGYSDRVAQLTLGNSTITTQEAANI
EV-E1 Vir 404/03 (DQ092771)	MGAQMSRNTAGSHTTGTYATGGSTINYNNINYYSNAASAAQNKQDFTQDPSKFTQPIADVIKETAVPLK	SPSAEACGYSDRVAQLTLGNSTITTQEAANI
EV-E2 PS83 (DQ092793)	MGAQLSRNTAGSHTTGTYAAGGSTINYNNINYYSNAASAAQNKQDFTQDPSKFTQPIADVIKETAVPLK	SPSAEACGYSDRVAQLTLGNSTITTQEAANI
EV-E2 PS42 (DQ092792)	MGAQLSRNTAGSHTTGTYAAGGSTINYNNINYYSNAASAAQNKQDFTQDPSKFTQPIADVIKETAVPLK	SPSAEACGYSDRVAQLTLGNSTITTQEAANI
EV-E2 SL305 (AF123433)	MGAQMSRSTAGSHTTGTYATGGSTINYNNINYYSHAASAAQNKQDFTQDPSKFTQPIVDVVKETAVPLK	SPSAEACGYSDRVAQLTLGNSTITTQEAANI
EV-E2 IS1/Bos_taurus/JPN/1990	MGAQMSRNTAGSHTTGTYATNGSTINYHNINYYSHAASAAQNKQDFTQDPSKFTQPIADVIKETAVPLK	SPSAEACGYSDRVAQLTLGNSTITTQEAANI
EV-E2 K2577 (AF123432)	MGAQMSRNTAGSHTTGTYATGGSTINYNNINYYSHAASAAQNKQDLTQDPAKFTQPIVDVIKETAVPLK	SPSAEACGYSDRVAQLTLGNSTITTQEAANI
EV-E3 HY12 (KF748290)	MGGQFSRNVAGSHTTRTYATGGSTINYHNINYYSSSASAAQNKQDLAQDPSKFTQPIADVIKEAAVPLK	SPSAEACGYSDRVAQLTLGNSTITTQEAANI
EV-F1 BEV-261 (NC_021220)	MGAQMSRNTAGSHTTSTYATGGSNIHYTNINYYENAASNSLNKQDFTQDPEKFTRPVVDVMKEAAVPLK	SPSAEACGYSDRVAQLTLGNSTITTQEAANI
EV-F1 IL/alpaca (KC748420)	MGAQLSKNTAGSHTTGTYATGGSNIHYTNINYYENAASNSLNKQDFTQDPEKFTRPVVDVMKEAAVPLK	SPPAEACGYSDRVAQLTLGNSTITTQEAANI
EV-F2 BHM26 (HQ917060)	MGAQVSKNTAGSHTTGTYATGGSNIHYTNINYYENAASNSLNKQDFTQDPEKFTRPVVDVMKEAVVPLK	SPSAEACGYSDRVAQLTLGNSTITTQEAANI
EV-F2 BJ001 (HQ663846)	MGAQVSKNTAGSHTTGTYATGGSNIHYTNINYYENAASNSLNKQDLTQDPEKFTRPVVDVMKEAAVPLK	SPSAEACGYSDRVAQLTLGNSTITTQEAANI
EV-F2 PS89 (DQ092795)	MGAQVSKNTAGSHTTRTYATGGSNIHYTNINYYENAASNSLNKQDFTQDPEKFTRPVVDVMKEAAVPLK	SPSAEACGYSDRVAQLTLGNSTITTQEAANI
EV-F2 3A (AY508697)	MGAQLSKNTAGSHTTGTYATGGSNIHYTNINYYENAASNSLNKQDFTQDPEKFTRPVVDVMKEAAVPLK	SPSAEACGYSDRVAQLTLGNSTITTQEAANI
Ho12/Bos taurus/JPN/2014	MGAQLSKNTAGSHTTGTYATGGSNIHYTNINYYENAASNSLNKQDFTQDPEKFTRPVVDVMKEAAVPLK	SPSAEACGYSDRVAQLTLGNSTITTQEAANI
IS2/Bos taurus/JPN/1990	MGAQVSKNTAGSHTTGTYATGGSNIHYTNINYYENAASNSLNKQDFTQDPDKFTRPVVDVMKEAAVPLK	SPSAEACGYSDRVAQLTLGNSTITTQEAANI
EV-F3 PS87/Belfast (DQ092794)	MGAQMSKNTAGSHTTGTYATGGSNIHYTNINYYENAASNSLNKQDFTQDPEKFTRPVVDVMKEAAVPLK	SPSAEACGYSDRVAQLTLGNSTITTQEAANI
EV-F4 Possum enterovirus W1 (AY462106)	MGAQLSKNTAGSHTTGTYATGGSNIHYTNINYYENAASNSLNKQDLTQDPEKFTRPVVDMMKEAAVPLK	SPSAEACGYSDRVAQLTLGNSTITTQEAANI
EV-F4 Possum enterovirus W6 (AY462107)	MGAQLSKNTAGSHTTGTYATGGSSIHYTNINYYENAASNSLNKQDFTQDPDKFTRPVADIMKEAAVPLK	SPSAEACGYSDRVAQLTLGNSTITTQEAANI
EV-A (NC 001612)	MGSQVSTQRSGSHENSNSASEGSTINYTTINYYKDAYAASAGRQDMSQDPKKFTDPVMDVIHEMAPPLK	SPSAEACGYSDRVAQLTIGNSTITTQEAANI
EV-B (NC 001472)	MGAQVSTQKTGAHETGLNASGNSIIHYTNINYYKDAASNSANRQDFTQDPGKFTEPVKDIMIKSMPALN	SPSAEECGYSDRVRSITLGNSTITTQECANV
EV-C (NC 002058)	MGAQVSSQKVGAHENSNRAYGGSTINYTTINYYRDSASNAASKQDFSQDPSKFTEPIKDVLIKTAPMLN	SPNIEACGYSDRVLQLTLGNSTITTQEAANS
EV-D (NC 001430)	MGAQVSRQQTGTHENANVATGGSSITYNQINFYKDSYAASASKQDFSQDPSKFTEPVAEALKAGAPVLK	SPSAEACGYSDRVLQLKLGNSSIVTQEAANI
EV-G (NC 004441)	MGMQMSKNTAGSHTTVTQASGGSHINYTNINYYSHSASASQNKQDITQDPSKFTQPMVDIMKESAVPLK	SPSAEACGYSDRVAQLTLGNSTITTQEAANI
EV-H (NC 003988)	MGAQVSRQTSGAHDTRIRAEQGANIHYTNINYYRDAASNAASKMDYSQDPDKFTKPVLDAITEPLPTLK	SPSAEACGYSDRVAQLTIGNSTITTQEAANV
EV-J (NC 010415)	MGAQVSRQQTGSHETSNIATNGSTLHYTNINYYNDSYAASAAKQDFTQDPGKFTQPVADVLKQTAVPLK	SPSAEACGYSDRVAQLKLGNTSITTQEAANI
RV-A (NC 001617)	MGAQVSRQNVGTHSTQNSVSNGSSLNYFNINYFKDAASSGASRLDFSQDPSKFTDPVKDVLEKGIPTLQ	SPTVEACGYSDRLIQITRGDSTITSQDTANA
RV-B (NC 001490)	MGAQVSTQKSGSHENQNILTNGSNQTFTVINYYKDAASTSSAGQSLSMDPSKFTEPVKDLMLKGAPALN	SPNVEACGYSDRVQQITLGNSTITTQEAANA
RV-C (NC 009996)	MGAQVSKQNVGSHENSVSATGGSVIKYFNINYYKDSASSGLTKQDFSQDPSKFTQPLAEALTN--PALM	SPTVEACGMSDRLKQITIGNSTITTQDTLNS

Strain	Sequence (continued)
AN12/Bos taurus/JPN/2014	CVGYGEWPSHLDDHDATAVDRPTHPGVSCERFYTLPSVEWTNTWKGQYWKLPNALTETGMFGQNAQFHYLYRGGWAVHVQCNATKFHQGLLLVVMIPECV
EV-E1 PA12-24791 (KC667561)	CVAYGCWPSKLSDTDATSVDKPTEPGVSAERFYTLRSKPWQADSKGWYWKLPDALNNTGMFGQNAQFHYIYRGGWAIHVQCNATKFHQGTLLVLAIPEHQ
EV-E1 VG-5-27 (D00214)	CVAYGCWPAKLSDTDATSVDKPTEPGVSAERFYTLRSKPWQADSKGWYWKLPDALNNTGMFGQNAQFHYIYRGGWAVHVQCNATKFHQGTLLVLAIPEHQ
EV-E1 LC-R4 (DQ092769)	CVAYGCWPAKLSDSDATSVDKPTEPGVSAERFYTLRSKPWQADSKGWYWKLPDALNNTGMFGQNAQFHYIYRGGWAIHVQCNATKFHQGTLLVLAIPEHQ
EV-E1 Vir 404/03 (DQ092771)	CVAYGCWPSKLSDSDATSVDKPTEPGVSAERFYTLRSKPWQADSKGWYWKLPDALNNTGMFGQNAQFHYIYRGGWAIHVQCNATKFHQGTLLVLAIPEHQ
EV-E2 PS83 (DQ092793)	CVAYGVWPSKLSDHDATSVDKPTEPGVSAERFYTLRSKAWGANSTGWYWKLPDALNNTGMFGQNAQFHYIYRGGWAIHVQCNATKFHQGTLLVVAIPEHQ
EV-E2 PS42 (DQ092792)	CVAYGVWPSKLSDHDATSVDKPTEPGVSAERFYTLRSKAWGANSTGWYWKLPDALNNTGMFGQNAQFHYIYRGGWAIHVQCNATKFHQGTLLVVAIPEHQ
EV-E2 SL305 (AF123433)	CVAYGTWPSKLSDHDATSVDKPTEPGVSAERFYTLRSKAWEANSPGWYWKLPDALNNTGMFGQNAQFHYIYRGGWAIHVQCNATKFHQGTLLVLAIPEHQ
EV-E2 IS1/Bos_taurus/JPN/1990	CVAYGTWPSKLSDHDATSVDKPTEPGVSAERFYTLRSKAWESTSRGWYWKLPDALNNTGMFGQNAQFHYIYRGGWAVHVQCNATKFHQGTLLVVAIPEHQ
EV-E2 K2577 (AF123432)	CVAYGTWPSKLSNHDATSVDKPTEPGVSAERFYTLRSKAWESNSTGWYWKLPDALNNTGMFGQNAQFHYIYRGGWAIHVQCNATKFHQGTLLVVAIPEHQ
EV-E3 HY12 (KF748290)	CVAYGTWPSKLSDTDATSVDKPTEPGVSAERFYTLRSKAWESTSTGWYWKLPDALNNTGMFGQNAQFHYIYRGGWAVHVQCNATKFHQGTLLVVAIPEHQ
EV-F1 BEV-261 (NC_021220)	VVAYGKWPEKLSDSDATAVDRPTEPGVSAERFYTLPSVQWTTTFKGRFWKLPDALSQLGLFGQNLQFHYLYRGGWAIHVQCNATKFHQGTLLVVAIPEHK
EV-F1 IL/alpaca (KC748420)	VVAYGKWPEKLSDSDATAVDRPTEPGVSAERFYTLPSVQWTNTFKGRFWKLPDALSQLGLFGQNLQFHYLYRGGWAIHVQCNATKFHQGTLLVVAIPEHK
EV-F2 BHM26 (HQ917060)	VVAYGQWPETLSDNDATAIDKPTEPGVSAERFYTLPSVQWTPTFKGRYWKLPDALSDLGLFGQNLQYHYLYRGGWAIHVQCNATKFHQGTLLVVAIPEHK
EV-F2 BJ001 (HQ663846)	VVAYGQWPEPLSDTDATAIDKPTEPGVSAERFYTLPSVQWTGTFPGRYWKLPDALSDLGLFGQNLQFHYLYRGGWAVHVQCNATKFHQGTLLVVAIPEHK
EV-F2 PS89 (DQ092795)	VVAYGQWPETLSDTDATAIDKPTEPGVSAERFYTLPSVQWTETFGGRYWKLPDALSELGLFGQNLQFHYLYRGGWAIHVQCNATKFHQGTLLVVAIPEHK
EV-F2 3A (AY508697)	VVAYGQWPETLSDTDATAIDKPTEPGVSAERFYTLPSVQWTEAFSGRYWKLPDALSDLGLFGQNLQFHYLYRGGWAIHVQCNATKFHQGTLLVVAIPEHK
Ho12/Bos taurus/JPN/2014	VVGYGRWPSSLKDTDATAVDKPTQPGVSAERFYTLPSVQWTNTFIGHYWKLPDALSELGLFGQNLQFHYLYRGGWVVHVQCNATKFHQGTLLVVATPEHK
IS2/Bos taurus/JPN/1990	VVGYGRWPSSLKDTDATAVDKPTQPGVSAERFYTLPSVQWTNNFPGHYWKLPDALSELGLFGQNLQFHYLYRGGWAVHVQCNATKFHQGTLLVVAVPEHK
EV-F3 PS87/Belfast (DQ092794)	VVGYGRWPTSLRDTDATAVDKPTQPGVSAERFYTLPSVQWTNSFKGHYWKLPDALSELGLFGQNLQFHYLYRGGWVIHVQCNATKFHQGTLLVVATPEHK
EV-F4 Possum enterovirus W1 (AY462106)	VVAYGRWPSNLRDTDATAVDKPTQPGVSAERFYTLPSVQWTTTFKGHYWKLPDALSELGLFGQNLQFHYLYRGGWAIHVQCNATKFHQGTLLVVAVPEHK
EV-F4 Possum enterovirus W6 (AY462107)	VVAYGRWPSGLRDTDATAVDKPTQPGVSAERFYTLPSVQWTTSFTGHYWKLPDALSDLGLFGQNLQFHYLYRGGWAIHVQCNATKFHQGTLLVVAVPEHK
EV-A (NC 001612)	IIAYGEWPEYCKDADATAVDKPTRPDVSVNRFFTLDTKSWAKDSKGWYWKFPDVLTEVGVFGQNAQFHYLYRSGFCVHVQCNASKFHQGALLVAILPEYV
EV-B (NC 001472)	VVGYGVWPEYLKDNEATGEDQPTQPDVATCRFYTLESVQWMKNSAGWWWKLPDALSQMGLFGQNMQYHYLGRTGYTIHVQCNASKFHQGCLLVVCVPEAE
EV-C (NC 002058)	VVAYGRWPEYLRDSEANPVDQPTEPDVAACRFYTLDTVSWTKESRGWWWKLPDALRDMGLFGQNMYYHYLGRSGYTVHVQCNASKFHQGALGVFAVPEMC
EV-D (NC 001430)	CCAYGEWPTYLPDNEAVAIDKPTQPETSTDRFYTLKSKKWESNSTGWWWKLPDALNQIGMFGQNVQYHYLYRSGFLCHVQCNATKFHQGTLLIVAIPEHQ
EV-G (NC 004441)	TVAYGEWPSYLSDLDATAVDKTTKPGVSCDRFYTLPGKKWEATTKGWEWKLPDALTELGVFGQNCQFHFLYRCGWSIHVQCNATKFHQGTLLVVAVPDHQ
EV-H (NC 003988)	VVAYGQWPEYLDSKDATAVDKPTQPDVASNRFYTLKTVSWEKSSTGWYWKFSDCLASVGLFGQNVQYHYLGRYGLAVHVQCNASKFHQGTLLVLAIPEWE
EV-J (NC 010415)	TVGYGEWPTFLSNVDANAVDAPTHPDVSVNRFYTLKAVXWEKTSTGWYWKLPDCLNQLGVFGQNAQYHYLYRSGFAVHVQCNASKFHQGALLVVAIPEWT
RV-A (NC 001617)	VVAYGVWPSYLTPDDATAIDKPTQPDTSSNRFYTLDSRSWTSASSGWWWKLPDALKNMGIFGENMFYHFLGRSGYTIHVQCNSSKFHQGLLIVAAIPEHQ
RV-B (NC 001490)	VVCYAEWPEYLPDVDASDVNKTSKPDTSVCRFYTLDSKTWTTGSKGWCWKLPDALKDMGVFGQNMFFHSLGRSGYTVHVQCNATKFHSGCLLVVVIPEHQ
RV-C (NC 009996)	ILAYGEWPKYLSDLDASSVDKPTHPETSSDRFYTLTSVDWTTTSKGWWWKLPDCLKDMGIFGQNLYHHALGRSGYIIHTQCNATKFNSGCLIVAVVPEHQ

Table S2. Multiple alignments result using amino acid sequences of polyprotein (continued)

AN12/Bos taurus/JPN/2014	V N - - - - - - - - L R G A P P I N K T Q P G E N G R E I E - - - - - - - - - - - - - E P F L L D S - - - - - G E A L G N A L I Y P H Q W I N L R T N N A A T I I V P Y V N A I P M D S A I R H S N W T
EV-E1 PA12-24791 (KC667561)	I A - - - - - - - - T Q E Q P V F D R T M P G S E G G A F Q - - - - - - - - - - - - - E P F W L E D - - - - - G T S L G N S L I Y P H Q W I N L R T N N S A T L I L P Y V N A I P M D S A I R H S N W T
EV-E1 VG-5-27 (D00214)	I A - - - - - - - - T Q E Q P A F D R T M P G S E G G T F Q - - - - - - - - - - - - - E P F W L E D - - - - - G T S L G N S L I Y P H Q W I N L R T N N S A T L I L P Y V N A I P M D S A I R H S N W T
EV-E1 LC-R4 (DQ092769)	I A - - - - - - - - T Q E Q P A F D R T M P G S D G G T F Q - - - - - - - - - - - - - E P F W L E D - - - - - G T S L G N S L I Y P H Q W I N L R T N N S A T L I L P Y V N A I P M D S A I R H S N W T
EV-E1 Vir 404/03 (DQ092771)	I A - - - - - - - - T Q E Q P A F D R T M P G S E G G T F Q - - - - - - - - - - - - - E P F W L E D - - - - - G T S L G N S L I Y P H Q W I N L R T N N S A T L I L P Y V N A I P M D S A I R H S N W T
EV-E2 PS83 (DQ092793)	I A - - - - - - - - T Q E Q P T F D R T M P G D T G G T F Q - - - - - - - - - - - - - E P F W L E D - - - - - G T S L G N A L I Y P H Q W I N L R T N N S A T L I L P Y V N A I P M D S A I R H S N W T
EV-E2 PS42 (DQ092792)	I A - - - - - - - - T Q E Q P T F D R T M P G D T G G T F Q - - - - - - - - - - - - - E P F W L E D - - - - - G T S L G N A L I Y P H Q W I N L R T N N S A T L I L P Y V N A I P M D S A I R H S N W T
EV-E2 SL305 (AF123433)	I A - - - - - - - - T Q E Q P Q F D R T M P G D A G G T F Q - - - - - - - - - - - - - E P F W L E D - - - - - G T S L G N S L I Y P H Q W I N L R T N N S A T L V L P Y V N A I P M D S A I R H S N W T
EV-E2 IS1/Bos_taurus/JPN/1990	I A - - - - - - - - T Q E Q P D F A R T M P G D T G G E F Q - - - - - - - - - - - - - E P F W L E D - - - - - G T S L G N A L I Y P H Q W I N L R T N N S A T L I L P Y V N A I P M D S A I R H S N W T
EV-E2 K2577 (AF123432)	I A - - - - - - - - T Q E Q P D F G R T M P G D E G G T F Q - - - - - - - - - - - - - E P F W L E D - - - - - G T C L G N A L V Y P H Q W I N L R T N N S A T L I L P Y V N A I P M D S A I R H S N W T
EV-E3 HY12 (KF748290)	I A - - - - - - - - T Q E Q P D F N R T M P G D Q G G T F Q - - - - - - - - - - - - - E A F W L E D - - - - - G T S L G N A L I Y P H Q W I N L R T N N S A T L I L P Y V N A I P M D S A I R H S N W T
EV-F1 BEV-261 (NC_021220)	I Q - - - - - - - - S Q Q T P E F A K T N P G E D G Y E F Q - - - - - - - - - - - - - F P F T L E D - - - - - G S A L G N A L I Y P H Q W I N L R T N N S A T L I L P Y V N A L P M D S G I R H N N W T
EV-F1 IL/alpaca (KC748420)	I Q - - - - - - - - S Q Q T P D F A K T N P G E D G F E F Q - - - - - - - - - - - - - F P F T L E D - - - - - G S A L G N A L I Y P H Q W I N L R T N N S A T L I L P Y V N A L P M D S G I R H N N W T
EV-F2 BHM26 (HQ917060)	I Q - - - - - - - - A Q Q T P D F T R T N P G E N G A E F N - - - - - - - - - - - - - F N F T L E D - - - - - G S A L G N A L I Y P H Q W I N L R T N N S A T L I L P Y V N A I P M D S G I R H N N W T
EV-F2 BJ001 (HQ663846)	I Q - - - - - - - - A Q Q T P D F D R T N P G E A G A N F N - - - - - - - - - - - - - F P F T L E D - - - - - G S A L G N A L V Y P H Q W I N L R T N N S A T L I L P Y V N A I P M D S G I R H N N W T
EV-F2 PS89 (DQ092795)	I Q - - - - - - - - A Q Q T P A F N R T N P G E D G A E F N - - - - - - - - - - - - - Y P F T L E D - - - - - G S A L G N A L V Y P H Q W V N L R T N N S A T L I L P Y V N A V P M D S G I R H N N W T
EV-F2 3A (AY508697)	I Q - - - - - - - - A Q Q T P A F D R T N P G E N G A E F R - - - - - - - - - - - - - F P F T L E D - - - - - G S A L G N A L I Y P H Q W V N L R T N N S A T L I L P Y V N A I P M D S G I R H N N W T
Ho12/Bos taurus/JPN/2014	I Q - - - - - - - - S A E A P A F E R T N P G E R G A A Y Q - - - - - - - - - - - - - F P F T F E D - - - - - G T A L G N A L I Y P H Q W I N L R T N N S A T L V L P Y V N A I P M D S G I R H N N W T
IS2/Bos taurus/JPN/1990	I Q - - - - - - - - S A E T P T F G R T N P G E A G A P Y Q - - - - - - - - - - - - - F P F T F E D - - - - - G T A L G N A L I Y P H Q W I N L R T N N S A T L V L P Y V N A I P M D S G I R H N N W T
EV-F3 PS87/Belfast (DQ092794)	I Q - - - - - - - - S A E S P A F A R T N P G E Q G A A Y Q - - - - - - - - - - - - - F P F T F E D - - - - - G T A L G N A L I Y P H Q W V N L R T N N S A T L V L P Y V N A L P M D S G I R H N N W T
EV-F4 Possum enterovirus W1 (AY462106)	I Q - - - - - - - - A Q S N P T F D R T N P G E N G A T C Q - - - - - - - - - - - - - F P F T F E D - - - - - G T A L G N A L I Y P H Q W I N L R T N N S A T L I L P Y V N A I P M D S G I K H N N W T
EV-F4 Possum enterovirus W6 (AY462107)	I Q - - - - - - - - A Q S N P S F G R T N P G E A G A A C Q - - - - - - - - - - - - - F P F T F E D - - - - - G T A L G N A L I Y P H Q W I N L R T N N S A T L V L P Y V N A I P M D S G I K H N N W T
EV-A (NC 001612)	L G T I A G G D G N E N S H P P Y V T T Q P G Q V G A V L T - - - - - - - - - - - - - N P Y V L D A - - - - - G V P L S Q L T V C P H Q W I N L R T N N C A T I I V P Y M N T V P F D S A L N H C N F G
EV-B (NC 001472)	M G - - - - - - - C S N L N N T P K F A E L S - - G G D N A R M F T D T E V G T S N D K K V Q T A V W N A G M G V G V G N L T I F P H Q W I N L R T N N S A T I V M P Y I N S V P M D N M Y R H N N L T
EV-C (NC 002058)	L A G D S N - - - T T T M H T S Y Q N A N P G E K G G T F T G T F T P D N N Q T S P A R R F C P V D Y L L G N G T L L G N A F V F P H Q I I N L R T N N C A T L V L P Y V N S L S I D S M V K H N N W G
EV-D (NC 001430)	I G - - - - - K K G T G T S A S F A E V M K G A E G G V F E - - - - - - - - - - - - - Q P Y L L D D - - - - - G T S L A C A L V Y P H Q W I N L R T N N S A T I V L P W M N S A P M D F A L R H N N W T
EV-G (NC 004441)	L G - - - - - - - - T T Y Q P E F D N V M P G K A G R E V K - - - - - - - - - - - - - Y P Y N F E D - - - - - G T S L A N S L I Y P H Q W I N L R T N N S A T L V L P Y A N A I P M D S P I R H S S W S
EV-H (NC 003988)	I G - - - - - - V S N A D R A S F N L T N P D K N G H T M T G Q - - - - - - - - - - - E A Y C L H N - - - - - G T N I H S S L V F P H Q F I N L R T N N C A T L V L P Y V G A T P L D T P I K H N V W S
EV-J (NC 010415)	Q G - - R G L T S S T N T Y M E Y S Q T N P G K N G G T F N - - - - - - - - - - - - - H P Y L L D N - - - - - G T A V S M A L I Y P H Q W I N L R T N N C A T I V L P Y V N N V P S D C A L K H N N W T
RV-A (NC 001617)	L A S A T - - - - S G N V S V G Y N H T H P G E Q G R E V V P S R T S S D N K R P S D D S W L N F D - - - - - G T L L G N L P I Y P H Q Y I N L R T N N S A T L I L P Y V N A V P M D S M L R H N N W S
RV-B (NC 001490)	L A S H E - - - - G G N V S V K Y T F T H P G E R G I D L S S A N E V G G - - - P V K D V I Y N M N - - - - - G T L L G N L L I F P H Q F I N L R T N N T A T I V I P Y I N S V P I D S M T R H N N V S
RV-C (NC 009996)	L A Y I G - - - - E A N V N V G Y D H T H P G E - G G H V I G S N V R R D N K Q P D E D P F F N C N - - - - - G T L L G N I T I F P H Q L I N L R T N N S S T I V V P Y I N C V P M D N M L R H N N L S

		VP3
AN12/Bos taurus/JPN/2014	L M V I P V T E L A Y A A N T S - P L V P I T V T L A P M C T E F G G L R A - - A S E - - Q	G L P V L A T P G S Y Q F L T T A D F Q T P C L L P K F E P S P E I H I P A E V R N L L E I V Q V E S M V E
EV-E1 PA12-24791 (KC667561)	L A I I P V A P L K Y A A E T T - P L V P I T V T I A P M E T E Y N G L R R - - A I A S N Q	G L P T K P G P G S Y Q F M T T D E D C S P C V L P D F Q P T P E I F I P G K V N N L L E I A Q V E S I V E
EV-E1 VG-5-27 (D00214)	L A I I P V A P L K Y A A E T T - P L V P I T V T I A P M E T E Y N G L R R - - A I A S N Q	G L P T K P G P G S Y Q F M T T D E D C S P C I L P D F Q P T P E I F I P G K V N N L L E I A Q V E S I L E
EV-E1 LC-R4 (DQ092769)	L A I I P V A P L K Y A A E T T - P L V P I T V T I A P M E T E Y N G L R R - - A I A S N Q	G L P T K P G P G S Y Q F M T T D E D C S P C I L P D F Q P T P E I F I P G K V N N L L E I A Q V E S I V E
EV-E1 Vir 404/03 (DQ092771)	L A I I P I A P L K Y A A E T T - P L V P I T V T I A P M E T E Y N G L R R - - A I A S N Q	G L P T K P G P G S Y Q F L T T D E D C S P C I L P D F Q P T P E I F I P G R V N N L L E I A Q V E S I V E
EV-E2 PS83 (DQ092793)	L A I I P V A P L R Y A A D T T - P L V P I T V T I A P M E T E Y N G L R R - - A V A S N Q	G L P T K P G P G S Y Q F M T S D E D C T P C I L P D F Q P T P M I F I P G E V T N L L Q I A Q V E S I V E
EV-E2 PS42 (DQ092792)	L A I I P V A P L R Y A A D T T - P L V P I T V T I A P M E T E Y N G L R R - - A V A S N Q	G L P T K P G P G S Y Q F M T S D E D C T P C I L P D F Q P T P M I F I P G E V T N L L Q I A Q V E S I V E
EV-E2 SL305 (AF123433)	L A I I P V A P L R Y A A D T T - P L V P I T V T I A P M E T E Y N G L R R - - A I A S N Q	G L P T K P G P G S Y Q F L T S D E D S T P C I L P D F Q P T P E I F I P G E V T N L L Q I A Q V E S I V E
EV-E2 IS1/Bos_taurus/JPN/1990	L A I I P V A P L K Y A A D T T - P L V P I T I T I A P M E T E Y N G L R R - - A I A S N Q	G L P T K P G P G S Y Q F M T S D E D S T P C I L P D F Q P T P E I F I P G E V T N L L Q I A Q V E S I V E
EV-E2 K2577 (AF123432)	L A I I P V A P L K Y A A D T T - P L V P I T V T I A P M E T E Y N G L R R - - A I A S N Q	G L P T K P G P G S Y Q F L T S D E D S T P C I L P D F Q P T P E I F I P G E V T N L L Q I A Q V E S I V E
EV-E3 HY12 (KF748290)	L A I I P I A P L K Y A A E T T - P L V P I T V T I A P M E T E Y N G L R R - - A I A S N Q	G L P T K P G P G S Y Q F M T T D E D C S P C I L P D F Q P T P E I F I P G R V N N L L E I V Q V E S I V E
EV-F1 BEV-261 (NC_021220)	L A V I P L V D L A Y A D G S T - T F V P I T V T I A P M A T E F N G L R R - - A V T - - Q	G L P T M Y T P G S G Q F L T T D D F Q S P S I L P K F K A T P E I E I P G R V L N I L E I V Q V E S L V E
EV-F1 IL/alpaca (KC748420)	L A V I P L V D L A Y A E G S T - T F V P I T V T I A P M A T E F N G L R R - - A V T - - Q	G L P T M Y T P G S G Q F L T T D D F Q S P S I L P K F K A T P E I E I P G R V L N I L E I V Q V E S L V E
EV-F2 BHM26 (HQ917060)	L A V I P V V D L A Y A T G S T - T Y V P I T I T I A P M A T E F N G L R R - - A V T - - Q	G L P T M Y T P G S G Q F L T T D D F Q A P C I L P K F E A S P E I K I P G E V K N I L E I V Q V E S L V E
EV-F2 BJ001 (HQ663846)	L A V I P I V P L A Y A T G S T - T Y V P I T I T I A P M A T E F N G L R R - - A I A - - Q	G L P T M Y T P G S G Q F L T T D D F Q A P C I L P K F Q A S P E I K I P G E V K N I L E I L Q I E S L V E
EV-F2 PS89 (DQ092795)	L A V I P I V P L A Y A V G S T - T Y V P I T I T I A P M A T E F N G L R R - - A I T - - Q	G L P T M Y T P G S G Q F L T T D D F Q A P C I L P K F Q A S P E I K I P G E V K N I L E I V Q V E S L V E
EV-F2 3A (AY508697)	L A V I P V V P L A Y A T G S T - T Y V P I T I T V A P M A T E F N G L R R - - A I A - - Q	G L P T M Y T P G S G Q F L T T D D F Q A P C I L P K F Q A S P E I K I P G E V K N I L E I V Q V E S L V E
Ho12/Bos taurus/JPN/2014	L S V I P L V P L E Y A A G A T - T Y V P I T V T V A P M C T E Y N G L R A - - A V T - - Q	G I P T M Y T P G S G Q F L T T D D F Q T P C M L P K F Q P T P V I D I P G E V K N F L E V I Q V E S L V E
IS2/Bos taurus/JPN/1990	L S V I P L V Q L E Y A A G A T - T Y V P I T V T I A P M C T E Y N G L R A - - A V T - - Q	G I P T M Y T P G S G Q F L T T D D F Q T P C M L P K F Q P T P V I D I P G E V R N F L E V I Q V E S L V E
EV-F3 PS87/Belfast (DQ092794)	L S V I P I V P L E Y A A G A T - T Y V P I T V T I A P M C T E Y N G L R A - - A V T - - Q	G I P T L Y T P G S G Q F L T T D D F Q T P C M L P K F Q P T P V I D I P G E V K N F L E V V Q V E S L V E
EV-F4 Possum enterovirus W1 (AY462106)	L L V I P L V P L E Y A A G A T - T F V P I T V T I A P M C T E Y N G L R A - - A I A - - Q	G I P T L Y T P G S G Q F L T T D D F Q S P C M L P K F Q P T P V I D I P G E V K N F L E V I Q V E S L V E
EV-F4 Possum enterovirus W6 (AY462107)	L L V I P L V P L E Y A V G A T - T F V P I T V T I A P M C T E Y N G L R A - - A V T - - Q	G C P T L Y T P G S G Q F L T T D D F Q T P C M L P K F Q P T P V I D I P G E V K N F L E V I Q V E S L V E
EV-A (NC 001612)	L I V V P V V P L D F N A G A T - S E I P I T V T I A P M C A E F A G L R Q - - A I K - - Q	G I P T E L K P G T N Q F L T T D D G V S A P I L P G F H P T P A I H I P G E V R N L L E I C R V E T I L E
EV-B (NC 001472)	L M I I P F V P L N Y S E G S S - P Y V P I T V T I A P M C A E Y N G L R - - - - L A S S Q	G L P V M T T P G S T Q F L T S D D F Q S P S A M P Q F D V T P E M Q I P G R V N N L M E I A E V D S V V P
EV-C (NC 002058)	I A I L P L A P L N F A S E S S - P E I P I T L T I A P M C C E F N G L R N I T - L P R L Q	G L P V M N T P G S N Q Y L T A D N F Q S P C A L P E F D V T P P I D I P G E V K N M M E L A E I D T M I P
EV-D (NC 001430)	L A V I P V C P L A G G T G N T N T Y V P I T I S I A P M C A E Y N G L R N - - A I T - - Q	G V P T C L L P G S N Q F L T T D D H S S A P A F P D F S P T P E M H I P G Q V H S M L E I V Q I E S M M E
EV-G (NC 004441)	L L V I P V V P L A C A T G T T - P F V G I T I T L A P M F S E F S G L R R - - A I A - - Q	G I P T T N T P G S Y Q F L T T D E D S S A C I L P D F T P T Q E I H I P G E V K N L Q A L C Q V E S L L E
EV-H (NC 003988)	L V V I P V V P L D Y T T G A T - T Q V P I T I T M A P M A C E F N G L R N - - A I T - - Q	G L P V L N T P G S G Q F V T T D N F Q S P N L I P N F D V T Q V F N S P G E I I N L Q Q Y V Q I E G I M E
EV-J (NC 010415)	L A V I P I S P L N Y D A G A T - P L V P I T I T I A P M A C E F S G L R K - - A I S - - Q	G L P A Q L T P G S T Q F V T T D N D S S Q P L I P N F D E T T P I H I P G E V R N M L E I C Q V E S I L E
RV-A (NC 001617)	L V I I P I C P L Q V Q P G G T - Q S I P I T V S I S P M F S E F S G P R S K V V F S T T Q	G L P V M L T P G S G Q F L T T D D T Q S P S A F P Y F H P T K E I F I P G Q V R N L I E M C Q V D T L I P
RV-B (NC 001490)	L M V I P I A P L T V P T G A T - P S L P I T V T I A P M C T E F S G I R S K - - S I V P Q	G L P T T T L P G S G Q F L T T D D R Q S P S A L P N Y E P T P R I H I L G K V H N L L E I I Q V D T L I P
RV-C (NC 009996)	L V I I P I V P L R A A N G - V - T K V P I T I S I A P D K S E F S G A R - - - - Q S V K Q	G L P T R L P S G S Q Q F L T T E D E Q S A N L L P D F S V T K I I D I P G R I T N I L H M A M V D S F M P

Table S2. Multiple alignments result using amino acid sequences of polyprotein (continued)

AN12/Bos taurus/JPN/2014	A N N L A D - - V E G A K R Y N I P L N V Q T G M D A Q I F A L N V D P G R D G P L Q H T L L G I Y T R Y F T Q W S G S L E F T F M F T G T F M T T G K I L I A Y T P P G A A V P A S R R E A M L G T H
EV-E1 PA12-24791 (KC667561)	A N N R E G - - V A G V D R Y V I P V S V Q D A L D A Q I Y A L K L E L G G T G P L S S S L L G T L A K H Y T Q W S G S V E I T C M F T G T F M T T G K V L L A Y T P P G G D M P R N R E E A M L G T H
EV-E1 VG-5-27 (D00214)	A N N R E G - - V E G V E R Y V I P V S V Q D A L D A Q I Y A L R L E L G G S G P L S S S L L G T L A K H Y T Q W S G S V E I T C M F T G T F M T T G K V L L A Y T P P G G D M P R N R E E A M L G T H
EV-E1 LC-R4 (DQ092769)	A N N R E G - - V E G V E R Y V I P V S V Q D A L D A Q I Y A L K L E L G G S G P L S S S L L G T L A K H Y T Q W S G S V E I T C M F T G T F M T T G K V L L A Y T P P G G D M P R N R E E A M L G T H
EV-E1 Vir 404/03 (DQ092771)	A N N R E G - - V E G V E R Y V I P V S V Q D A L D A Q I Y A L K L E L G G T G P L S S S L L G T L A K H Y T Q W S G S V E I T C M F T G T F M T T G K V L L A Y T P P G G D M P R N R E E A M L G T H
EV-E2 PS83 (DQ092793)	A D N V A D - - A I G V E R Y V I P I S V Q D R L D S Q I Y V L K L E L G G R G P L S S T L L G T M A K H F T Q W S G S V E I T C M F T G T F M T T G K V L L A Y T P P G G D M P R N R D E A M L G T H
EV-E2 PS42 (DQ092792)	A N N V A D - - V I G V E R Y V I P I S V Q D R L D S Q I Y V L K L E L G G T G P L S S T L L G T M A K H F T Q W S G S V E I T C M F T G T F M T T G K V L L A Y T P P G G D M P R N R D E A M L G T H
EV-E2 SL305 (AF123433)	A N N T N D - - A N G V E R Y V I P V G V Q D A L D S Q I Y V L K L E L G G S G P L S S S L L G T M A K H F T Q W S G S V E I T C M F T G T F M T T G K V L L A Y T P P G G D M P R N R D E A M L G T H
EV-E2 IS1/Bos_taurus/JPN/1990	A N N V A D - - A T G V E R Y V I P V S V Q D R L D S Q I Y V L K L E L G G T G P L S S S L L G T L A K H F T Q W S G S V E I T C M F T G T F M T T G K V L L A Y T P P G G D M P R N R E E A M L G T H
EV-E2 K2577 (AF123432)	A N N T A D - - A A G V E R Y V I P V S V Q D A L D S Q I Y V L K L E L G G T G P L S S T L L G T M A K H F T Q W S G S V E I T C M F T G T F M T T G K V L L A Y T P P G G D M P R N R D E A M L G T H
EV-E3 HY12 (KF748290)	A N N R N G - - V L G V E R Y V I P I S V Q D A L D S Q I Y A L K L E L G G T G P L S S S L L G T M A K H F T Q W S G S I E I T C M F T G T F M T T G K V L L A Y T P P G G D M P R N R E E A M L G T H
EV-F1 BEV-261 (NC_021220)	I N N V D Q - - V N G V A R Y R I P L S V Q D D M D Q Q I M A L R V D P G T S G P L Q S T L L G V F S R Y Y T Q W S G S I E F T F M F C G T F M S T G K V I I A Y T P P G G T A P T S R R E A M L G T H
EV-F1 IL/alpaca (KC748420)	I N N V E G - - S S G V A R Y R I P L S V Q D D M D Q Q I M A L R V D P G T N G P L Q S T L L G V F T R Y Y T Q W S G S V E F T F M F C G T F M S T G K V I I A Y T P P G G A A P T S R R E A M L G T H
EV-F2 BHM26 (HQ917060)	I N N V E A - - A T G I A R Y R I P L S V Q D D M D Q Q I M A L R V D P G V D G P L Q S T L L G V F T R Y Y T Q W S G S V E F T F M F C G T F M S T G K V V V A Y T P P G G T A P T T R R E A M L G T H
EV-F2 BJ001 (HQ663846)	I N N V E G - - E D G V T R Y R I P L S V Q D E M D Q Q I M A L C V D P G I N G P L Q S T L L G V F T R Y Y T Q W S G S I E F T F M F C G T F M S T G K V V I A Y T P P G G A A P T S R R D A M L G T H
EV-F2 PS89 (DQ092795)	I N N V D G - - Q D G V A R Y R I P L S V Q D A M D Q Q I M A L R V D P G V D G P L Q S T L L G V F T R Y Y T Q W S G S V E F T F M F C G T F M S T G K V V V A Y T P P G G T A P T T R R D A M L G T H
EV-F2 3A (AY508697)	I N N V E G - - Q S G V A R Y R I P L S V Q D A M D Q Q I M A L R V D P G I D G P L Q S T L L G V F T R Y Y T Q W S G S V E F T F M F C G T F M S T G K V V I A Y T P P G G T A P T T R R D A M L G T H
Ho12/Bos taurus/JPN/2014	I N N V A D - - A E G V A R Y R I P L S V Q D A M D G Q I M A L R V D P G I D G P M Q S T L L G V F T R Y Y A Q W S G S I E F T F M F C G T F M T T G K V I I A Y T P P G G D Q P T S R Q Q A M L G T H
IS2/Bos taurus/JPN/1990	I N N V D N - - V E G V A R Y R I P L N V Q D A M D G Q I M A L R V D P G I D G P M Q S T L L G V F T R Y Y A Q W S G S L D F T F M F C G T F M T T G K V I I A Y T P P G G D Q P T S R Q Q A M L G T H
EV-F3 PS87/Belfast (DQ092794)	I N N V E S - - A E G V A R Y R I P L N V Q D A M D G Q I M A L R V D P G I D G P M Q S T L L G V L H R Y Y A Q W S G S L D F T F M F C G T F M T T G K V I I A Y T P P G G D Q P T A R R Q A M L G T H
EV-F4 Possum enterovirus W1 (AY462106)	I N N V V N - - V E G V E R Y R I P L N V Q D A M D G Q I M A M R V D P G A D G P L Q S T L L G V F T R Y Y T Q W S G S L E F T F M F C G T F M T T G K V I I A Y T P P G G D Q P E S R Q Q A M L G T H
EV-F4 Possum enterovirus W6 (AY462107)	I N N V S G - - V E G V A R Y R I P L N V Q D A M D G Q I M A V R V D P G A D G P M Q S T L L G V F T R Y Y T Q W S G S L D F T F M F C G T F M T T G K V I I A Y T P P G G D Q P G S R Q Q A M L G T H
EV-A (NC 001612)	V N N L Q S N E T T P M Q R L C F P V S V Q S K T G E L C A V F R A D P G R N G P W Q S T I L G Q L C R Y Y N Q W S G S L E V T F M F A G S F M A T G K M L I A Y T P P G G V P A D R L T A M L G T H
EV-B (NC 001472)	V N N T D N N - V N G L K A Y Q I P V Q S N S D N R R Q V F G F P L Q P G A N N V L N R T L L G E I L N Y Y T H W S G S I K L T F M F C G S A M A T G K F L L A Y S P P G A G V P K N R R D A M L G T H
EV-C (NC 002058)	F D L S A T K - K N T M E M Y R V R L S D K P H T D D P I L C L S L S P A S D P R L S H T M L G E I L N Y Y T H W A G S L K F T F L F C G F M M A T G K L L V S Y A P P G A D P P K K R K E A M L G T H
EV-D (NC 001430)	I N N V N D - - A S G V E R L R V Q I S A Q S D M D Q L L F N I P L D I Q L E G P L R N T L L G N I S R Y Y T H W S G S L E M T F M F C G S F M T T G K L I I C Y T P P G G S S P T D R M Q A M L A T H
EV-G (NC 004441)	I N N V D G - - K T G I E R L R L E V S T Q S E L D R Q L F A L K V S F T E G E I M S K T L L C G V M C S Y Y T Q W S G S L E I T F M F T G S F M T T G K L L L A Y T P P G G S A P A S R E D A M L G T H
EV-H (NC 003988)	I N N V A S - - A N N L E R I R I P I S V Q S G I D E M L F A I N C N P G T A Q E F R R T P L G D V C R Y Y T Q W S G S I Q I T F T F C G S F M T T G K L L I C Y T P P G G R V P Q N R E E A M L G T N
EV-J (NC 010415)	V N N V T G - - V N G M S R L L I P I S V Q S D L D Q Q I A S F R V D P G A D G P W Q S T L L G N I C R Y Y N Q W S G S L E I T F M F T G S F M A T G K I L I G Y S P P G G S Q P A T R E D A M L G T Y
RV-A (NC 001617)	V N N T Q E N - V R S V N M Y T V D L R T Q V D L A K E V F S I P V D I A - S Q P L A T T L I G E L A S Y Y T H W T G S L R F S F M F C G S A S S T L K L L I A Y T P P G V G K P K S R R E A M L G T H
RV-B (NC 001490)	M N N T H T - - K D E V N S Y L I P L N A N R Q - N E Q V F G T N L F I G - D G V F K T T L L G E I V Q Y Y T H W S G S L R F S S M Y T G P A L S S A K L T L A Y T P P G A R G P Q D R R E A M L G T H
RV-C (NC 009996)	L N N I E T N - V G Q I S I Y N V Q V - T K K T A D K P I V V I P M D I S - N T L F S T T L L G E V L N Y F A N W S G S L S I T F M C V C D S F S T G K F L L A Y T P P G A A L P P D R K T A M L G V H

AN12/Bos taurus/JPN/2014	V V W D F G L Q S S I T L V V P W I S A A H F R G T D S T S T L Y K Y K Y Y E A G V I T G W Y Q T N M I T P P D F P Q T A S I V A F V A A Q S N F S L R I Q K D R P D M T Q T A S L Q - - - - - - - - -
EV-E1 PA12-24791 (KC667561)	V V W D F G L Q S S I T L V I P W I S A S H F R G V S N D D I L N - Y Q Y Y A A G H V T I W Y Q T N M V I P P G F P N T A G I I M M I A A Q P N F S F R I Q K D R E D M T Q T A V L Q N - - - - - - - -
EV-E1 VG-5-27 (D00214)	V V W D F G L Q S S I T L V I P W I S A S H F R G V S N D D V L N - Y Q Y Y A A G H V T I W Y Q T N M V I P P G F P N T A G I I M M I A A Q P N F S F R I Q K D R E D M T Q T A I L Q N - - - - - - - -
EV-E1 LC-R4 (DQ092769)	V V W D F G L Q S S I T L V I P W I S A S H F R G V S S D D T L N - Y Q Y Y A A G H I T I W Y Q T N M V I P P G F P N T A G I I M M I A A Q P N F S F R I Q K D R E D M T Q T A V L Q N - - - - - - - -
EV-E1 Vir 404/03 (DQ092771)	V V W D F G L Q S S I T L I I P W I S A S H F R G V A N D D T L N - Y Q Y Y A A G H V T I W Y Q T N M V I P P G F P N T A G I I M M I A A Q P N F S F R I Q K D R E D M T Q T A I L Q N - - - - - - - -
EV-E2 PS83 (DQ092793)	V I W D F G L Q S S I T L V V P W I S A S H F R G V S V D D T L N - Y Q Y Y A A G Y V T M W Y Q T N M V I P P G F P N T A G I I M L V A A Q P N F S F R I Q K D R E D M T Q T A A L Q N - - - - - - - -
EV-E2 PS42 (DQ092792)	V I W D F G L Q S S I T L V V P W I S A S H F R G V S V D D T L N - Y Q Y Y A A G Y V T M W Y Q T N M V I P P G F P N T A G I I M L V A A Q P N F S F R I Q K D R E D M T Q T A A L Q N - - - - - - - -
EV-E2 SL305 (AF123433)	V I W D F G L Q S S I T L V V P W I S A S H Y R G V S T D D S L N - Y Q Y Y A A G Y V T M W H Q T N M V I P P G F P N T A G I I M L V A A Q S N F S F R I Q K D R E D M A Q T A A L Q N - - - - - - - -
EV-E2 IS1/Bos_taurus/JPN/1990	V I W D F G L Q S S I T L V V P W I S A S H F R G V S V D D T L N - Y Q Y Y A A G Y V T M W Y Q T N M V I P P G F P N T A G I I M L V A A Q P N F S F R I Q K D R E D M T Q T A A L Q N - - - - - - - -
EV-E2 K2577 (AF123432)	V I W D F G L Q S S I T L V I P W I S A S H F R G V S V D D T L N - Y Q Y Y A A G Y V T M W Y Q T N M V I P P G F P N T A G I V M L V A A Q P N F S F R I Q K D R E D M T Q T A A L Q N - - - - - - - -
EV-E3 HY12 (KF748290)	V I W D F G L Q S S I T L V V P W I S A S H F R G V S V D D T L N - Y Q Y Y A S G H I T I W Y Q T N M V I P P G F P N V A G I I M L V A A Q P N F S F R I Q K D R E D M I Q T A A L Q N - - - - - - - -
EV-F1 BEV-261 (NC_021220)	I V W D F G L Q S S I T L V V P W I S S G H F R A A D I N A T L F K Y K Y Y E A G F V T M W Y Q T N L V V P P S F P N Q A A I L M F V A A Q P N F S M R I L K D R P E M T Q T A A L Q G - - - - - - - -
EV-F1 IL/alpaca (KC748420)	I V W D F G L Q S S I T L V V P W I S S G H F R A A D I N A T L F K Y K Y Y E A G F V T M W Y Q T N L V V P P S F P N Q A T I L M F V A A Q P N F S M R I L K D R P E M T Q T A A L Q G - - - - - - - -
EV-F2 BHM26 (HQ917060)	I I W D F G L Q S S I T L V V P W I S S G H F R G V A A N D T T F K Y R Y Y E T G F I T M W Y Q T N L V V P P A F P N D A S I I M F V A A Q P N F S L R I M K D R P E M T Q T A A L Q G - - - - - - - -
EV-F2 BJ001 (HQ663846)	I V W D F G L Q S S I T L I V P W I S S G H F R G V A A D E S T F K Y R Y Y E T G F V T M W Y Q T N F V V P P Q F P N E A S I I M F V A A Q P N F S L R I M K D R P E M A Q S A A L Q G - - - - - - - -
EV-F2 PS89 (DQ092795)	I I W D F G L Q S S I T L V V P W I S S G H F R G V T A D D T T F K Y R Y Y E T G F I T M W Y Q T N L V V P P Q F P N D A S I I M F V A A Q P N F S L R I M K D R P E M S Q T A A L Q G - - - - - - - -
EV-F2 3A (AY508697)	I V W D F G L Q S S I T L V V P W I S S G H F R G V A A D D S N F K Y R Y Y E T G F I T M W Y Q T N L V V P P Q F P N D A S I I M L V A A Q P N F S L R I M K D R P E M T Q T A A L Q G - - - - - - - -
Ho12/Bos taurus/JPN/2014	V V W D F G L Q S S I T L V V P W I S S G H F R G T T L E N T I Y K Y R Y Y E A G Y I T M W Y Q T N M V V P P N F P T G A S V L M F V A A Q S N F S L R I L K D R P D I S Q V G A L Q G - - - - - - - -
IS2/Bos taurus/JPN/1990	V V W D F G L Q S S I T L V V P W I S S G H F R G T T L E N T I Y K Y R Y Y E A G Y I T M W Y Q T N M V V P P N F P T T A S V L M F V A A Q P N F S L R I L K D R P D I S Q E G A L Q G - - - - - - - -
EV-F3 PS87/Belfast (DQ092794)	V V W D F G L Q S S I T L V V P W I S S G H F R G T T L E N T I Y K Y R Y Y E R G Y I T M W Y Q T N M V V P P N F P N H A S I L M F V A A Q P N F S L R I L K D R P D I S Q E G A L Q G - - - - - - - -
EV-F4 Possum enterovirus W1 (AY462106)	V V W D F G L Q S S I T L V V P W I S S G H F R G T S L D N T I Y K Y R Y Y E A G Y I T M W Y Q T N M V V P P N F P T T A S I L M F V A A Q P N F S L R I L K D R P D V S Q T A A L Q G - - - - - - - -
EV-F4 Possum enterovirus W6 (AY462107)	V V W D F G L Q S S I T L V V P W I S S G H F R G T S L D N T I Y K Y R Y Y E A G Y I T M W Y Q T N M V V P P N F P T E A S I L M F V A A Q P N F S L R I L K D R P D I T Q V A S L Q G - - - - - - - -
EV-A (NC 001612)	V I W D F G L Q S S V T L V I P W I S N T H Y R A H A K D G Y F D - - - Y Y T T G T I T I W Y Q T N Y V V P I G A P T T A Y I V A L A A A Q D N F T M K L C K D T E D I E Q S A N I Q G - D - - - - - -
EV-B (NC 001472)	V I W D V G L Q S S C V L C V P W I S Q T H Y R Y V E D E - - - - - - Y T A A G Y V T C W Y Q T N I I V P A D V Q S T C D I L C F V S A C N D F S V R M L K D T P F I R Q D - N F Y Q G P - - - - -
EV-C (NC 002058)	V I W D I G L Q S S C T M V V P W I S N T T Y R Q T I D D S - - - - - - F T E G G Y I S V F Y Q T R I V V P L S T P R E M D I L G F V S A C N D F S V R L L R D T T H I E Q K - A L A Q G L G Q M L E S
EV-D (NC 001430)	V V W D F G L Q S S I T I I I P W I S G S H Y R M F N T D A K A I - - - N A N V G Y V T C F M Q T N L V A P V G A A D Q C Y I V G M V A A K K D F N L R L M R D S P D I G Q S A I L P E Q - - - - - - -
EV-G (NC 004441)	V I W D F G L Q S S I T L V V P W I C G G Y Y R D V N - - - - - R A N N Y Y A A G Y V T G W F Q T N M V A P P D F P S T A Y I L C F L A A Q P N F S L R I L K D R P D I T Q T A A L Q A - - - - - - - -
EV-H (NC 003988)	V I W D F G L Q S S V T L N I P W I S G A H F R N - T S V N V D G - - - F D N T G Y V S A W F Q T N M V V P P D A P T T A Y I L A F T S A K D D F S M R L L R D T A E I S Q D G F L Q G - - - - - - -
EV-J (NC 010415)	V V W D F G L Q S S V T L A I P W I S N V M F R N L Q S I A G Q P - - - Y F Y A G I V T M W Y Q T N F V V P D G A P T S A D I I A L G S A Q S N F S L R L L K D S P D I S Q T A V L N G P N - - - - - -
RV-A (NC 001617)	L V W D V G L Q S T A S L V V P W V S A S H F R F T T P D T - - - - - - Y S S A G Y I T C W Y Q T N F V V P D S T P D N A K M V C M V S A C K D F C L R L A R D T N L H T Q E G V L T Q N P - - - - - -
RV-B (NC 001490)	V V W D I G L Q S T I V M T I P W T S G V Q F R Y T D P D T - - - - - - Y T S A G F L S C W Y Q T S L I L P P E T T G Q V Y L L S F I S A C P D F K L R L M K D T Q T I S Q T V A L T E G L - - - - - -
RV-C (NC 009996)	V I W D L G L Q S S C T I V V P W M S G T F H R R T K A D N - - - - - - Y T Q A G Y V S L W Y Q T D F V S P N D G I G - - T I V A T C S G C P D L S V R M L R D T P M M K Q E K D L T Q N P - - - - - -

Table S2. Multiple alignments result using amino acid sequences of polyprotein (continued)

VP1

AN12/Bos taurus/JPN/2014	----SPKEAIKGAVDK-------------VVG-TVATEHNISTNNTPALQAAETGATSTATDEGMIETRHVVATHGVAETSLESFYGRSSLVGMP-----
EV-E1 PA12-24791 (KC667561)	----DPGKVLKDAIDKQVAGA--------LVAGTSTSTHSVATDSTPALQAAETGATSTARDESMIETRTIVSTHGIHETSVESFFGRSSLVGMP-----
EV-E1 VG-5-27 (D00214)	----DPGKMLKDAIDKQVAGA--------LVAGTTTSTHSVATDSTPALQAAETGATSTARDESMIETRTIVPTHGIHETSVESFFGRSSLVGMP-----
EV-E1 LC-R4 (DQ092769)	----DPGKMLKDVIDKQVAGA--------LVAGTSTSTHTVATDSTPALQAAETGATSTARDENMIETRTVVPTHGIHETSVESFFGRSSLVGMP-----
EV-E1 Vir 404/03 (DQ092771)	----DPGSVLKDAIDKQVASA--------LVAGTTTSSHGVATDSTPALQAAETGATSTARDEGMIETRTIVPTHGIHETSVESFFGRSSLVGMP-----
EV-E2 PS83 (DQ092793)	----DPGTALEAAIQKQVAGA--------LVAGTTASTHSIATNSTPALQAAETGATSTAEDSSMIETRTIVPTHGVHETSVESFFGRSALVGMP-----
EV-E2 PS42 (DQ092792)	----DPGTALEAAIQKQVAGA--------LVAGTTASTHSIATDSTPALQAAETGATSTAEDSSMIETRTIVPTHGVHETSVESFFGRSALVGMP-----
EV-E2 SL305 (AF123433)	----DPGKALESAIQKQVANA--------LVAGTTQSSHSIATDSTPALQAAETGATSTASDESMIETRTVVPTHGVHETSVESFYGRSALVGMV-----
EV-E2 IS1/Bos_taurus/JPN/1990	----DPGKTLEAAIQKQVAGA--------LVAGTTSSTHSIATDSTPALQAAETGATSTAQDESMIETRTIVPTHGVHETSVESFYGRSALVGMV-----
EV-E2 K2577 (AF123432)	----DPGKALESAIQKQVANA--------LVAGTTSSTHSIATDSTPALQAAETGATSTAQDESMIETRTIIPTHGVHETSVESFYGRSALVGMV-----
EV-E3 HY12 (KF748290)	----DPGKVLKDIIDTQVAGA--------LVAGTATSTHAIATDATPALQAAETGATSTASDESMIETRTIVPTHGIHETSVESFFGRSALVGMP-----
EV-F1 BEV-261 (NC_021220)	----DVKDSIKGAVAT-------------TVKNTTESHHSISTENTPALQAAETGATSNASDEGLIETRNVVNTHGVEETSVEAFFGRSGLVGMT-----
EV-F1 IL/alpaca (KC748420)	----DVKDVIKSAVST-------------TVKNTTESHHSISTENTPALQAAETGATSNASDEGLIETRNVVNTHGVEETSVEAFFGRSGLVGMT-----
EV-F2 BHM26 (HQ917060)	----ETGLAIKQAVHK-------------TVANTVESHHTISTESTPALQAAETGATSNASDESLVETRNVVNLHSVAETSVEAFFGRSGLVGMA-----
EV-F2 BJ001 (HQ663846)	----ETGLAIKQAVHK-------------TVANTVESHHTVSTENTPALQAAETGATSNASDESLIETRNVVNVHSVAETSVEAFFGRSGLIGMA-----
EV-F2 PS89 (DQ092795)	----ETGLALKQAVHK-------------TVANTLESHHTVSTENTPALQAAETGATSNASDESLIETRNVVNTHSVAETSIEAFFGRSGLVGMA-----
EV-F2 3A (AY508697)	----ETGLAIKQAVHK-------------TVANTVESHHTVSTESTPALQAAETGATSNASDESLIETRNVVNTHSVAETSIEAFFGRSGLVGMA-----
Ho12/Bos taurus/JPN/2014	----ETGQVVKAAVKS-------------TVENTVQSTHSISTDATPALQAAETGATSNTSDEAMIETRNVVNTHGVAETSLEAFYGRAGLVAMF-----
IS2/Bos taurus/JPN/1990	----ETGQAIKNAIKS-------------TVENTVESTHSISTEATPALQAAETGATSNASDESMIETRNVVNTHGVAETSLEAFYGRAGLVAMF-----
EV-F3 PS87/Belfast (DQ092794)	----ETGQVIKSAVRS-------------TVENTVQSTHSITTEATPALQAAETGATSNASDESMIETRNVVNTHGVAETSLEAFYGRAGLVAMF-----
EV-F4 Possum enterovirus W1 (AY462106)	----ETGHMIKDTIKD-------------TVENTVQSTHSISTEATPALQAAETGATSNTSDESMLETRNVINTHGVAETSLEGFYGRAGLVAMF-----
EV-F4 Possum enterovirus W6 (AY462107)	----ETGQLIKDTIKN-------------TVENTVESTHSISTEATPALQAAETGATSNASDESMIETRNVVNTHGVAETSLEGFYGRAGLVAMF-----
EV-A (NC 001612)	----GIADMIDQAVTSRVGRALTSLQVEPTAANTNASEHRLGTGLVPALQAAETGASSNAQDENLIETRCVLNHHSTQETTIGNFFSRAGLVSIITMPTT
EV-B (NC 001472)	-----VEESVERAMVR---------------VADTVSSKPTNSESIPALTAAETGHTSQVVPSDTMQTRHVKNYHSRSESSIENFLCRSACVYYATYNN-
EV-C (NC 002058)	MIDNTVRETVGAATSRD-------------ALPNTEASGPTHSKEIPALTAVETGATNPLVPSDTVQTRHVVQHRSRSESSIESFFARGACVTIMTVDNP
EV-D (NC 001430)	----AATTQIGEIVKT--------------VANTVESEIKAELGVIPSLNAVETGATSNTEPEEAIQTRTVINMHGTAECLVENFLGRSALVCMRSFEYK
EV-G (NC 004441)	----PVETALNSAISSVIAGI--------TAQDTQPSSHNISTSETPALQAAETGASSNASDEGMMETRHVVNTNTVSETSIESFYGRCGLVSIK-----
EV-H (NC 003988)	----PIDQAIEKVITDVVSD-------------TRESSSDFSIGAVPALNAVETGATSQASVESTIETRAVQNRHRTSEMSVESFLGRSSLVTRFTINNG
EV-J (NC 010415)	----PVGQALDKMVTNKIEG----------LSNTSASDAVIETANTPALQAAETGATSNADDAGLIETRDVINTQRPTETTVQAFYARAGLVGMVDIIHS
RV-A (NC 001617)	-----VENYIDSVLNEVL------------VVPNIQPSTSVSSHAAPALDAAETGHTSSVQPEDMIETRYVITDQTRDETSIESFLGRSGCIAMIEFNTS
RV-B (NC 001490)	-----GDELEEVIVEKTK------------QTVASISSGPKHTQKVPILTANETGATMPVLPSDSIETRTTYMHFNGSETDVECFLGRAACVHVTEIQNK
RV-C (NC 009996)	-----VEDYIDKVVDTVL------------QVPNTQPSGPQHSIQPSALGAMEIGASSTTIPGDLIETRYVINSNTNSEALIENFMGRSALWAKIQVAN-

AN12/Bos taurus/JPN/2014	------SLNPGDTVTEWIINFGEFVQLRAKLCLFTYMRFDIEFTIVATAYSATGDPISDP--TYITYQVMYVPPGASAPTDQDTYQWQSGANPSLLSRVG
EV-E1 PA12-24791 (KC667561)	------LLATGSNITYWRIDFREFVQLRAKMSWFTYMRFDVEFTIIATSSTGQNVTTEQ----HTTYQVMYVPPGAPVPSNQDSFQWQSGCNPSVFADTD
EV-E1 VG-5-27 (D00214)	------LLATGTSITNWRIDFREFVQLRAKMSWFTYMRFDVEFTIIATSSTGQNVTTEQ----HTTYQVMYVPPGAPVPSNQDSFQWQSGCNPSVFADTD
EV-E1 LC-R4 (DQ092769)	------LLATGSSVTSWRIDFREFVQLRAKMSWFTYMRFDVEFTIIATSSTGQNVTTEQ----HTTYQVMYVPPGAPVPSNQDSFQWQSGCNPSVFADTD
EV-E1 Vir 404/03 (DQ092771)	------LLAAGSGVTYWRIDFREFVQLRAKMSWFTYMRFDVEFTIIATSSTSQGVTTEQ----STTYQVMYVPPGAPVPSNQDSFQWQSGCNPSVFSDTD
EV-E2 PS83 (DQ092793)	------TLQAGSRVTNWRIDFREFVQLRAKMSWFTYMRFDVEFTIVARTAN-ATAAVEH----RNRFQVMYVPPGAPQPADQDSFQWQSGCNPSVIADVE
EV-E2 PS42 (DQ092792)	------TLQAGSRVTNWRIDFREFVQLRAKMSWFTYMRFDVEFTIVARTAN-ATAAVEH----RNRFQVMYVPPGAPQPADQDSFQWQSGCNPSVIADVE
EV-E2 SL305 (AF123433)	------TLPSGSRVVNWVIGFREFVQLRAKMSWFTYMRFDVEFTIVATTAS-STAAVEH----RNRFQVMYVPPGAPQPADQDSFQWQSGCNPSVIADVE
EV-E2 IS1/Bos_taurus/JPN/1990	------TLPSGSRVIQWRIDFREFVQLRAKMSWFTYMRFDVEFTIVATTAN-ATAAVEH----RNRFQVMYVPPGAPQPADQDSFQWQSGCNPSVIADVE
EV-E2 K2577 (AF123432)	------TLASGSRVISWRIDFREFVQLRAKMSWFTYMRFDVEFTIVATTAS-ATAAVEH----RNRFQVMYVPPGAPQPADQDSFQWQSGCNPSAIADVE
EV-E3 HY12 (KF748290)	------ILENGSRVTMWRIDFREFVQLRAKMSWFTYMRFDVEFTIVATTAN-TSGSVDQ----NNRFQVMYVPPGAPQPADQDSYQWQSGCNPSVIADTE
EV-F1 BEV-261 (NC_021220)	------STVGG--IGRWIVNFGEFVQVRAKLELFTYVRFDIELTIVAEVLTQTGNKVAH---QHVNYQVMYVPPGASEPENQDSYQWQSSCNPSVISNTA
EV-F1 IL/alpaca (KC748420)	------STVGG--IGRWIVNFGEFVQVRAKLELFTYVRFDIELTIVAEVLTQTGNKVAH---QHVNYQVMYVPPGASEPENQDSYQWQSSCNPSVISNTA
EV-F2 BHM26 (HQ917060)	------TTTGG--IGRWIVNFGEFVQVRAKIELFTYIRFDIELTIVGEVLTMQGDKVSH---EHTKYQVMYVPPGATLPENQDTYQWQSSCNPSVISSTD
EV-F2 BJ001 (HQ663846)	------ETVGG--IGRWIVNFGEFVQVRAKLELFTYIRFDIELTIVGEVLDAN-SKVEH---VPAKYQVMYVPPGATLPENQDTFQWQSSCNPSIISSTD
EV-F2 PS89 (DQ092795)	------ETIGG--IGRWIVNFGEFVQVRAKLELFTYVRFDIELTIVGEVLDAK-SKVEH---VPVKYQVMYVPPGATLPENQDSFQWQSSCNPSVISSTE
EV-F2 3A (AY508697)	------ETIGG--IGRWIVNFGEFVQVRAKLELFTYVRFDIELTIVGEVLDAN-SKVEH---VPVKYQVMYVPPGATLPENQDTFQWQSSCNPSVISSTE
Ho12/Bos taurus/JPN/2014	------STDGG--IYRWYINFGEYVQLRAKLELLTYARFDIEFTIVAQVVNAQ-AKVQD---FNVDYQVMYVPPGASVPENQDSFQWQSSCNPSVISNTG
IS2/Bos taurus/JPN/1990	------STDGG--IYRWYINFGEYVQLRAKLELLTYARFDIEFTIVAQVVNAQ-AKVLD---FNVDYQVMYVPPGASVPENQDSYQWQSSCNPSVISNTG
EV-F3 PS87/Belfast (DQ092794)	------STDGG--IYRWYINFGEYVQLRAKLELLTYARFDMEFTIVAQVVNAQ-SKVQD---FNVDYQVMFVPPGASVPENQDSYQWQSSCNPSVISNTG
EV-F4 Possum enterovirus W1 (AY462106)	------TTDGG--IRSWYINFGEYVQLRTKLELLTYARFDIEFTIVAQAVDEQ-AKVKD---FNVDYQVMYVPPGASAPDGQDSFQWQSSCNPSVFSNTG
EV-F4 Possum enterovirus W6 (AY462107)	------TTEGG--IRSWYINFGKYVQLRAKLELLTYARFDVEFTIVAQVVDEQ-AKVKD---FNVDYQVMYVPPGASAPDGQDSFQWQSSCNPSVISNTG
EV-A (NC 001612)	------GTQNTDGYVNWDIDLMGYAQMRRKCELFTYMRFDAEFTFVAAKPNGE--LVP------QLLQYMYVPPGAPKPTSRDSFAWQTATNPSIFVKLT
EV-B (NC 001472)	--------NSEKGYAEWVINTRQVAQLLRRKLEFTYLRFDLELTFVITSAQEPSTATSVD-APVQTQQIMYVPPGGPVPTKVTDYAWQTSTNPSVFWTEG
EV-C (NC 002058)	AST----TNKDKLFAVWKITYKDTVQLRRKLEFFTYSRFDMELTFVVTAN--FTETNNGH-ALNQVYQIMYVPPGAPVPEKWDDYTWQTSSNPSIFYTYG
EV-D (NC 001430)	NHS-TSTSSIQKNFFIWTLNTRELVQIRRKMELFTYLRFDTEITIVPTLRLFSSSNVSFSGLPNLTLQAMYVPTGARKPSSQDSFEWQSACNPSVFFKIN
EV-G (NC 004441)	------EIADNKQVEKWLVNFNEFVQLRAKIELFTYMRFDIEFTLVATFTKGNSASQHP-----VQVQVMYLPPEQLLQLQQDSYAWQSAANPSAIFSAN
EV-H (NC 003988)	------GTNNATKFRNWKINLKEVVQLRRKLEMFTYVRFDLEVTIVAVNLTGN--GGVR-----YMYQAMYCPPGAPLPTNADQYLWQSSTNPSIIGAVG
EV-J (NC 010415)	-------DSNSPKFASWTIDTMAFVQQRRKLEMFTYMRFAAEFTFVVSTESTT--KIPN-----TMIQYMYVPPGAPIPTTSNGYEWQSGTNPSVIVRMN
RV-A (NC 001617)	SDK-TEHDKIGKGFKTWKVSLQEMAQIRRKYELFTYTRFDSEITIVTAAAAQ--GNDSGH-I---VLQFMYVPPGAPVPEKRDDYTWQSGTNASVFWQEG
RV-B (NC 001490)	DATGIDNHREAKLFNDWKINLSSLVQLRKKLELFTYVRFDSEYTILATASQPDSANYSSN-L---VVQAMYVPHGAPKSKRVGDYTWQSASNPSVFFKVG
RV-C (NC 009996)	------------GFAKWDINFQEHAQVRKKFEMFTYARFDMEVTVVTNNTG--------------LVQIMFVPPGIDAPDSIDSRLWDSASNPSVFYQPK

Table S2. Multiple alignments result using amino acid sequences of polyprotein (continued)

AN12/Bos taurus/JPN/2014	D V P A A F S V P F M G T A N A Y S Y A Y D G Y A F F N - - T T D H - - - - L D Y G V L P S N Y L G S M Y F R T L E R V T - - - Q K I R F R I Y A K P K H V R A W V P R A P R A V P Y Q S R Y N N S Y T
EV-E1 PA12-24791 (KC667561)	G P P A Q F S V P F M S S A N A Y S T V Y D G Y A R F M - - D T D P - - - - D R Y G I L P S N F L G F M Y F R T L E D A A - - - H Q V R F R I Y A K I K H T S C W I P R A P R Q A P Y K K R Y N L V F S
EV-E1 VG-5-27 (D00214)	G P P A Q F S V P F M S S A N A Y S T V Y D G Y A R F M - - D T D P - - - - D R Y G I L P S N F L G F M Y F R T L E D A A - - - H Q V R F R I C A K I K H T S C W I P R A P R Q A P Y K K R Y N L V F S
EV-E1 LC-R4 (DQ092769)	G P P A Q F S V P F M S S A N A Y S T V Y D G Y A R F M - - D T E P - - - - D R Y G I L P S N F L G F M Y F R T L E D A A - - - H Q V R F R I Y A K I K H T S C W I P R A P R Q A P Y K K R Y N L V F S
EV-E1 Vir 404/03 (DQ092771)	G P P A Q F S V P F M S S A N A Y S N V Y D G Y A R F M - - D T D P - - - - D R Y G I L P S N F L G F M Y F R T L E D A A - - - H Q V R F R I Y A K I K H T S C W I P R A P R Q A P Y K K R Y N L V F S
EV-E2 PS83 (DQ092793)	G P P V Q F S V P F M S T A N A Y S N F Y D G Y A R F M - - D T N P - - - - D R Y G L L P S N F L G L M Y F R C L E D T T - - - D A V R F R I Y A K I K H T H C W I P R A P R Q A P Y K K R Y N L V F D
EV-E2 PS42 (DQ092792)	G P P V Q F S V P F M S T A N A Y S N F Y D G Y A R F M - - D T N P - - - - D R Y G L L P S N F L G L M Y F R C L E D T T - - - D A V R F R I Y A K I K H T H C W I P R A P R Q A P Y K K R Y N L V F D
EV-E2 SL305 (AF123433)	G P P V Q F S V P F M S T A N A Y S N F Y D G Y A R F M - - D T H P - - - - D R Y G I L P S N F L G L M Y F R C L E D T T - - - D N V R F R I Y A K I K H T R C W I P R A P R Q A P Y K K R Y N L V F D
EV-E2 IS1/Bos_taurus/JPN/1990	G P P V Q F S V P F M S T A N A Y S N F Y D G Y A R F M - - D T H P - - - - D R Y G L L P S N F L G L M Y F R C L E D T T - - - D S V R F R I Y A K I K H T R C W I P R A P R Q A P Y K K R Y N L V F D
EV-E2 K2577 (AF123432)	G P P V Q F S V P F M S T A N A Y S N F Y D G Y A R F M - - D T N P - - - - D R Y G L L P S N F L G L M Y F R C L E D T T - - - D N V R F R I Y A K I K H T R C W I P R A P R Q A P Y K K R Y N L V F D
EV-E3 HY12 (KF748290)	G P P V Q F S V P F M S T A N A Y S T V Y D G Y A R F M - - N T D P - - - - D K Y G I L P S N F L G L M Y F R C L E D T H - - - T Q M R F R I Y A K I K H T Q C W I P R A P R Q A P Y K K R Y H L V F G
EV-F1 BEV-261 (NC_021220)	A P P A R V S I P F M S T A N A Y S M S Y D G Y A T F D - - D T A G - - - - S N Y G M V P S N Y L G T I V F R T M E D L D Q - - K L L K V R F Y A K P K H L K C W M P R A P R A V P Y K S R Y T G V Y D
EV-F1 IL/alpaca (KC748420)	A P P A R V S I P F M S T A N A Y S M S Y D G Y A T F D - - D T A G - - - - S N Y G M V P S N Y L G T I V F R T M E D L D Q - - K L L K V R F Y A K P K H L K C W M P R A P R A V P Y K S R Y T G V Y D
EV-F2 BHM26 (HQ917060)	L P P A R V S I P F M S T A N A Y S M S Y D G Y A T F G - - D T A G - - - - G N Y G M V P S N Y L G T I V F R T M E D L D G - - L K L K L R F Y A K P K H V K C W I P R A P R A V P Y K S R Y T G V Y D
EV-F2 BJ001 (HQ663846)	L P P A R V A I P F M S T A N A Y S M S Y D G Y A T F G - - N T G G - - - - D N Y G M V P S N Y L G T I V F R T I E D L D G - - L K L K L R F Y A K P K H V K C W I P R A P R A V P Y K S R Y T G V Y D
EV-F2 PS89 (DQ092795)	L P P A R V A I P F M S T A N A Y S M S Y D G Y A T F G - - D T G G - - - - A N Y G I V P S N Y L G T I V F R T I E D L D G - - L K L K L R F Y A K P K H V K C W I P R A P R A V P Y K S R Y T G V Y D
EV-F2 3A (AY508697)	L P P A R V A I P F M S T A N A Y S M S Y D G Y A T F G - - D T G G - - - - A N Y G M V P S N Y L G T I V F R T I E D L D G - - L K L K L R F Y T K P K H V K C W I P R A P R A V P Y K S R Y S G V Y D
Ho12/Bos taurus/JPN/2014	L P P A R V S V P F M S S A N A Y S F S Y D G Y T Q F G - - D T S G - - - - S S Y G I V P S N Y L G Q L V V R T C E D L D G - - T R L R V R I Y A K P K H I R G W I P R S P R M R P Y K S R Y T G V Y S
IS2/Bos taurus/JPN/1990	M P P A R V S V P F M S S A N A Y S F S Y D G Y T Q F G - - D T S G - - - - S S Y G I V P S N Y L G Q L V V R T C E D L D G - - T R L R V R I Y A K P K H I R G W I P R S P R M R P Y K S R Y T G V Y T
EV-F3 PS87/Belfast (DQ092794)	L P P A R V S V P F M S S A N A Y S F S Y D G Y T Q F G - - D T S G - - - - S S Y G I V P S N Y L G M L V V R T C E D L D G - - T R L R V R V Y A K P K H V K G W I P R S P R M T P Y K S R Y T G V Y T
EV-F4 Possum enterovirus W1 (AY462106)	M P P A R V S V P F M S S A N A Y S F S Y D G Y T E F G - - D K S G - - - - S S Y G I L P S N Y L G Q L V V R T C E D L D S - - A H L R V R I Y A K P K H M R G W I P R S P R M R P Y V S R F T G V Y T
EV-F4 Possum enterovirus W6 (AY462107)	L P P A R V S V P F M S S A N A Y S F S Y D G Y I Q F G - - D T S S - - - - S S Y G I L P I H Y L G Q L V V R T C E D L D S - - A R L R V R I Y A K P K H M R G W I P R S P R M R P Y V S R F T G I Y T
EV-A (NC 001612)	D P P A Q V S V P F M S P A S A Y Q W F Y D G Y P T F G A H P Q S N - - - D A D Y G Q C P N N M M G T F S I R T V G T E K S P - H S I T L R V Y M R I K H V R A W I P R P L R N Q P Y L F K T N P N Y K
EV-B (NC 001472)	N A P P R M S I P F I S I G N A Y S C F Y D G W T Q F S R - - - - - - - - N G V Y G I N T L N N M G T L Y M R H V N E A G Q G P I K S T V R I Y F K P K H V K A W V P R P P R L C Q Y E K Q K N V N F N
EV-C (NC 002058)	T A P A R I S V P Y V G I S N A Y S H F Y D G F S K V P L K D Q S A A L G D S L Y G A A S L N D F G I L A V R V V N D H N P T K V T S K I R V Y L K P K H I R V W C P R P P R A V A Y Y G - P G V D Y K
EV-D (NC 001430)	D P P A R L T I P F M S I N S A Y A N F Y D G F A G F E - - K K A T - - - - V L Y G I N P A N T M G N L C L R V V N S Y Q P V Q Y T L T V R V Y M K P K H I K A W A P R A P R T M P Y T N I L N N N Y A
EV-G (NC 004441)	T V P A R F S V P F V G T A N A Y T I M Y D G Y N V F G - - S N R P S - - - A D Y G M I N S S H M G S M A F R A I S Q L Q A T - E K V K F M D L C Q V K D V R A W C P R A P R M A P Y K Y I R N P V F E
EV-H (NC 003988)	E V P G R V S V P F V S N A N M Y A T F Y D G Y P S F G S I N G Q G N - - G S D Y G A F I P N D M G T L C F R L L N I F N N G - P Q I Q F R V F M K P K H V R V W C P R P C R T L Q Y V K L G T P R Y S
EV-J (NC 010415)	E A P A R V R V P F I S P A G A Y A W F Y D G Y T D F G T H P S P N G K G D A N Y G L N P A N Q F G T F A T R I I G N G - A I - S A I R I R I Y M K P T Q V R C W I P R A I R T C P Y I E K N R P S Y N
RV-A (NC 001617)	Q P Y P R F T I P F M S I A S A Y Y M F Y D G Y D G - - D S A - - - - - - A S K Y G S V V T N D M G T I C V R I V T S N Q K H D S N I V C R I Y H K A K H I K A W C P R P P R A V A Y Q H T H S T N Y I
RV-B (NC 001490)	- D T S R F S V P Y V G L A S A Y N C F Y D G Y S H - - D D A - - - - - - E T Q Y G I T V L N H M G S M A F R I V N E H D E H K T L V K I R V Y H R A K L V E A W I P R A P R A L P Y T S I G R T N Y P
RV-C (NC 009996)	S G F P R F T I P F T G L G S A Y Y M F Y D G Y D V P R N K S - - - - - - N A V Y G I T S T N D M G T L C F R A M E D T N E H S - - - - I R V F V K P K H T I A W I P R P P R A T Q Y T H K F S T N Y H

		P2 protein 2A
AN12/Bos taurus/JPN/2014	A - - - - - - - - - - - V T A L A P N R D T I T T L -	G P L R T T G P F G Q Q S G A A Y V G N Y K I M N R H L A D C V D W Q N L V W E S Y E R D L L V
EV-E1 PA12-24791 (KC667561)	G - - - - - - - - - - D T D R I C N N R A S L T S Y -	- - - - - - G P F G Q Q Q G A A Y V G S Y K I V N R H L A T Y T D W E N E V W Q S Y Q R D L L V
EV-E1 VG-5-27 (D00214)	G - - - - - - - - - - D S D R I C S N R A S L T S Y -	- - - - - - G P F G Q Q Q G A A Y V G S Y K I L N R H L A T Y A D W E N E V W Q S Y Q R D L L V
EV-E1 LC-R4 (DQ092769)	G - - - - - - - - - - D T D R I C S N R A S S T S Y -	- - - - - - G P F G Q Q Q G A A Y V G S Y K I V N R H L A T Y T D W E N E V W Q S Y Q R D L L V
EV-E1 Vir 404/03 (DQ092771)	G - - - - - - - - - - D T D R I C S N R A S L T S Y -	- - - - - - G P F G Q Q Q G A A F V G N Y K I V N R H L A T V G D W E N E V W Q S Y Q R D L L V
EV-E2 PS83 (DQ092793)	G - - - - - - - - - - A T D K I C M D R S S L T T L -	- - - - - - G P F G Q Q Q G A A F V G C Y K I V N R H L A T V Q D W E N E V W Q S Y Q R D L L V
EV-E2 PS42 (DQ092792)	G - - - - - - - - - - A T D K I C M D R S S L T T L -	- - - - - - G P F G Q Q Q G A A F V G C Y K I V N R H L A T V Q D W E N E V W Q S Y Q R D L L V
EV-E2 SL305 (AF123433)	G - - - - - - - - - - T T D R I C T S R S S L T T L -	- - - - - - G P F G Q Q Q G A A F V G S Y K I V N R H L A T Y T D W E N E V W Q S Y Q R D L L V
EV-E2 IS1/Bos_taurus/JPN/1990	G - - - - - - - - - - T T D R I C T D R S T L T T L -	- - - - - - G P F G Q Q Q G A A Y V G S F K I V N R H L A T Y S D W E N E V W Q S Y Q R D L L V
EV-E2 K2577 (AF123432)	G - - - - - - - - - - T T D R I C T N R S S L T T L -	- - - - - - G P F G Q Q Q G A A Y V G G Y K I V N R H L A T Y Q D W E N E V W Q S Y Q R D L L V
EV-E3 HY12 (KF748290)	G P - - - - - - - N - F Q D K I C A D R A S L T S Y -	- - - - - - G P F G Q Q Q G A A Y V G S Y K I V N R H L A T Y A D W E N E V W Q S Y Q R D L L V
EV-F1 BEV-261 (NC_021220)	T - - - - - - - - - - - V E K F C D D R R S I T T A -	- - - - - - G A F G Q Q S G A A Y V G N Y K I M N R H L A E K Q D W D N L V W D S Y E R D L L V
EV-F1 IL/alpaca (KC748420)	T - - - - - - - - - - - V E R F C D N R S S I T T A -	- - - - - - G A F G Q Q S G A V C V G S Y R I L N R H L A T T N D W E N V V W E S Y E R D L L V
EV-F2 BHM26 (HQ917060)	A - - - - - - - - - - - V Q K F C P N R D S I T T T -	- - - - - - G A F G Q Q S G A A Y V G N Y K I M N R H L A E K Q D W D N L V W D S Y E R D L L V
EV-F2 BJ001 (HQ663846)	A - - - - - - - - - - - V Q K F C V N R D S I T T T -	- - - - - - G A F G Q Q S G A V C V G S Y R I L N R H L A T E S D W E N V V W E S Y E R D L L V
EV-F2 PS89 (DQ092795)	A - - - - - - - - - - - V Q K F C A N R A D I K T T -	- - - - - - G A F G Q Q S G A V C V G S Y R I L N R H L A T Q A D W D N V V W D S Y E R D L L V
EV-F2 3A (AY508697)	T - - - - - - - - - - - V E S F C A N R A N I K T T -	- - - - - - G A F G Q Q S G A V C V G S Y R I L N R H L A T Q A D W N S V I W D S Y E R D L L V
Ho12/Bos taurus/JPN/2014	N - - - - - - - - - - - T T K F C A D R G T I T T A -	- - - - - - G A F G Q Q S G A A Y V G N Y K I M N R H L A E K A D W D N L V W D S Y E R D L L V
IS2/Bos taurus/JPN/1990	D - - - - - - - - - - - A T K F C A D R A R I T T A -	- - - - - - G A F G Q Q S G A A Y V G N Y K I M N R H L A E Q A D W D N L V W D S Y E R D L L V
EV-F3 PS87/Belfast (DQ092794)	D - - - - - - - - - - - T T K F C A N R A R I T T A -	- - - - - - G A F G Q Q S G A A Y V G N Y K I M N R H L A E K Q D W D N L V W D S Y E R D L L V
EV-F4 Possum enterovirus W1 (AY462106)	D - - - - - - - - - - - V P R F C V N R D S I K T A -	- - - - - - G A F G Q Q S G A A Y V G S Y K I M N R H L A K E Q D W K N H I W D S Y E R D L L V
EV-F4 Possum enterovirus W6 (AY462107)	D - - - - - - - - - - - A P S F C V N R E S I T T A -	- - - - - - G A F G Q Q S G A I Y V G C Y K V L N R H L A V E Q D W E N V V W D S Y E R D L L V
EV-A (NC 001612)	G N - - - - - - - D - - I K C T S T S R D K I T T L -	- - - - - - G K F G Q Q S G A I Y V G N Y R V V N R H L A T H N D W A N L V W E D S S R D L L V
EV-B (NC 001472)	P T - - - - - - - - - - - - G V T T T R S N I T T T -	- - - - - - G A F G Q Q S G A V Y V G N Y R V V N R H L A T R E D W Q R C V W E D Y N R D L L V
EV-C (NC 002058)	D G - - - - - - - - - - - - - T L T P L S T K D L T T Y -	- - - - - - - G F G H Q N K A V Y T A G Y K I C N Y H L A T Q D D L Q N A V N V M W S R D L L V
EV-D (NC 001430)	G R S A A - - - - P N A P T A I V S H R S T I K T M P N D I N L T T A G P G Y - - - - - - - - - - - - -	- - - - - - - - - - - - - G G A F V G S Y K I I N Y H L A T D E E K E R S V Y V D W Q S D V L V
EV-G (NC 004441)	T - - - - - - - - - - - Q D R I V P N R N N I T T T -	- - - - - - G A F G Q Q S G A I Y V G N Y K I M N R H L A T H E D W E N V E W E D Y N R D I L V
EV-H (NC 003988)	N Q - - - - - - - D T H L N K M T T D R A N I K T V T N Y I M S G S T R T V E S E V A V V E D S L K Q Y	- - - - - - - - - G P A T G G V V V G Q Y K I I N Y H L A T T E D R E K E I W S D P R R D L L V
EV-J (NC 010415)	P N - - - - - - - - - - K T A I A K N R Q S I T T T -	- - - - - - G K F G Q Q S G A V Y V G N Y R I V N R H L A N E F D A Q S M V W E S Y E R D L L V
RV-A (NC 001617)	P S N G E A T T Q I K T R P D V F T V T N V G P S S M F -	- - - - - - - - - - - - - - - V H V G N L I Y R N L H L F N S D L - D D S I L V S Y S S D L I I
RV-B (NC 001490)	K N - - - - - - - - - T E P V I K K R K G D I K S Y -	- - - - - - - G L G P R Y G G I Y T S N V K I M N Y H L M T P E D H H N L I A P Y P N R D L A I
RV-C (NC 009996)	V K K P D D T T G L L I Q K H F I N H R T D I K T A -	- - - - - - - - - G P S D L F V H T A T M I Y R N A H L T S L N - - D K T V L L A L S A D L Q V

Table S2. Multiple alignments result using amino acid sequences of polyprotein (continued)

AN12/Bos taurus/JPN/2014	T R V D A H G C D Q I A R C D C R A G V Y Y C K S R M K H Y P V V V T P P S L V H V D K N D Y Y P E R Y Q S H V A L G I G F A E P G D C G G L L R C E H G V M G I L T A G G N N L V A F A D I R D L L W
EV-E1 PA12-24791 (KC667561)	T R V D A H G C D T I A R C N C R S G V Y Y C K S R N K H Y P I V V T P P S I F K I E A N D Y Y P E R M Q T H I L L S I G F A E P G D C G G L L R C E H G V M G I L T V G G G D L V G F A D I R D L L W
EV-E1 VG-5-27 (D00214)	T R V D A H G C D T I A R C N C R S G I Y Y C K S T A K H Y P I V V T P P S I Y K I E A N D Y Y P E R M Q T H I L L G I G F A E P G D C G G L L R C E H G V M G I L T V G G G D H V G F A D V R D L L W
EV-E1 LC-R4 (DQ092769)	T R V D A H G A D T I A R C N C R S G V Y Y C K S R N K H Y P I V V T P P S I F K I E A N D Y Y P E R M Q T H I L L G I G F A E P G D C G G L L R C E H G V M G I L T V G G G D L V G F A D I R D L L W
EV-E1 Vir 404/03 (DQ092771)	T R V D A H G C D K I A R C S C R S G V Y F C K S R N K H Y P I V V T P P S I F K I E A N D Y Y P E R M Q T H I L L G I G F A E P G D C G G L L R C E H G V M G I L T V G G S D L V G F A D I R D L L W
EV-E2 PS83 (DQ092793)	T R I D A H G C D T I A R C N C R S G V Y Y C K S R N K H Y P I V V T P P S I F K I E A N D Y Y P E R M Q T H I L L G I G F A E P G D C G G L L R C E H G V M G I L T V G G G N L V G F A D I R D L L W
EV-E2 PS42 (DQ092792)	T R I D A H G C D T I A R C N C R S G V Y Y C K S R N K H Y P I V V T P P S I F K I E A N D Y Y P E R M Q T H I L L G I G F A E P G D C G G L L R C E H G V M G I L T V G G G N L V G F A D I R D L L W
EV-E2 SL305 (AF123433)	T R V D A H G C D T I A R C N C R S G V Y Y C K S R N K H Y P I V V T P P S I F K I E A N D Y Y P E R M Q T H I L L G T G F A E P G D C G G L L R C E H G V M G I L T V G G G D L V G F A D I R D L L W
EV-E2 IS1/Bos_taurus/JPN/1990	T R V D A H G C D T I A R C N C R S G V Y Y C K S R N K H Y P I V V T P P S I F K I E A N D Y Y P E R M Q T H I L L G I G F A E P G D C G G L L R C E H G V M G I L T V G G G D L V G F A D I R D L L W
EV-E2 K2577 (AF123432)	T R V D A H G C D T I A R C N C R S G V Y F C K S K N K H Y P I V V T P P S I F K I E A N D Y Y P E R M Q T H I L L G I G F A E P G D C G G L L R C E H G V M G I L T V G G G D L V G F A D I R D L L W
EV-E3 HY12 (KF748290)	T R V D A H G R D T I A R C N C R A G V Y Y C K S R N K H Y P I V V T P P S I F K I E A N N Y Y P E R M Q T H I L L G I G F A E P G D C G G L L R C E H G V M G I L T V G G G N L V G F A D I R D L L W
EV-F1 BEV-261 (NC_021220)	T R V E A H G R D Q I A R C Q C T A G V Y Y C K S R M K H Y P V I V T P P V L Y K I G A S D Y Y P E R Y Q S H V A L G I G F A E P G D C G G I L R C Q H G V M G I L T A G G N N M V A F A D V R Q L L W
EV-F1 IL/alpaca (KC748420)	M R H D A A G S D T I A R C N C T S G V Y Y C K S R N K H Y P V V I T P P S L V H V D A N D Y Y P E R Y Q S H V L L G I G F A E P G D C G G I L R C Q H G V M G V L T A G G N N L V A F A D V R D L L W
EV-F2 BHM26 (HQ917060)	T R V D A H G R D Q I A R C D C R A G V Y Y C K S R M K H Y P V V V T P P S L V H V D K N D Y Y P E R Y Q S H V A L G I G F A E P G D C G G L L R C E H G V M G I L T A G G S N L V A F A D I R D L L W
EV-F2 BJ001 (HQ663846)	M R H D A A G S D V I A R C N C T S G V Y Y C K S R G K H Y P V V V T P P S L V H V D A N D Y Y P E R Y Q S H V L L G I G F A E P G D C G G I L R C Q H G C M G I L T A G G N N L V A F A D L R D L L W
EV-F2 PS89 (DQ092795)	M R H D A A G S D T I A R C N C T S G V Y Y C K S R N K H Y P A V V T P P S L V H V D T N D Y Y P E R Y Q S H V L L G I G F A E P G D C G G I L R C Q H G C M G I L T A G G N N L V A F A D L R D L L W
EV-F2 3A (AY508697)	M R H D A A G S D T I A R C S C T S G V Y Y C K S R N K H Y P V V V T P P S L V H V G A N D Y Y P E R Y Q S H V L L G I G F A E P G D C G G I L R C Q H G C M G I L T A G G N N L V A F A D L R D L L W
Ho12/Bos taurus/JPN/2014	A R V D A H G C D Q I A R C D C R A G V Y Y C K S R M K H Y P V V V T P P S L V H I D K N H Y Y P E R Y Q S H V A L G I G F A E P G D C G G L L R C E H G V M G I L T A G G N N L V A F A D V R D L L W
IS2/Bos taurus/JPN/1990	T R V D A H G C D Q I A R C D C R A G V Y Y C K S R M K H Y P V V V T P P S L V H I D K N D Y Y P E R Y Q S H V A L G I G F A E P G D C G G L L R C E H G V M G I L T A G G N N L V A F A D I R D L L W
EV-F3 PS87/Belfast (DQ092794)	T R V D A H G C D Q I A R C N C R A G V Y Y C K S K M K H Y P V V V T P P S L V H I D K N D Y Y P E R Y Q S H V A L G I G Y A E P G D C G G L L R C E H G V M G I L T A G G N N L V A F A D V R D L L W
EV-F4 Possum enterovirus W1 (AY462106)	T R V D A H G K D Q I A R C S C C A G V Y Y S K S R N K H Y P V V V T P P S L A H I D E N D Y Y P E R Y Q S H V I L G V G F A E P G D C G G I L R C E H G V M G I L T A G G S N L V A F A D V R D L L W
EV-F4 Possum enterovirus W6 (AY462107)	T R A D A H G T D Q I A R C D C R A G V Y Y C K S R K R H Y P V V V S P P S L V H V D K N D Y Y P E R F Q S H V T L G V G F A E P G D C G G I L R C E H G V M G I L T A G G N N L V A F A D V R D L L W
EV-A (NC 001612)	S S T T A Q G C D T I A R C D C Q T G V Y Y C S S R R K H Y P V S F S K P S L I F V E A S E Y Y P A R Y Q S H L M L A V G H S E P G D C G G I L R C Q H G V V G I V S T G G N G L V G F A D V R D L L W
EV-B (NC 001472)	S T T T A H G C D I I A R C Q C T T G V Y F C A S R N K H Y P V S F E G P G L V E V Q E S E Y Y P K R Y Q S H V L L A A G F S E P G D C G G I L R C E H G V V G I V T M G G E G V V G F A D V R D L L W
EV-C (NC 002058)	T E S R A Q G T D S I A R C N C N A G V Y Y C E S R R K Y Y P V S F V G P T F Q Y M E A N N Y Y P A R Y Q S H M L I G H G F A S P G D C G G I L R C H H G V I G I I T A G G E G L V A F S D I R D L Y A
EV-D (NC 001430)	T T V A A H G K H Q I A R C R C N T G V Y Y C K H K N R S Y P V C F E G P G I Q W I N E S D Y Y P A R Y Q T N T L L A M G P C Q P G D C G G L L V C S H G V I G L V T A G G E G I V A F T D I R N L L W
EV-G (NC 004441)	A R T T A H G A D K L A R C H C N T G V Y Y C K S R N K H Y P V T F Q G P G I D W V E A S Q Y Y P A R Y Q T H M L L A S G I S E P G D C G G I L R C Q H G V I G V V T A G G Q G V V G F A D V R D L F W
EV-H (NC 003988)	C A S S V H G N N W I A R C A C R T G V Y W S R T Y G K C F P V C F Q G P G I E K F Q A S E Y Y P E R Y Q T H V L L A M G P A Q P G D C G G L L C C P H G V I G L L T G G D E G R V A F A D I R D L L W
EV-J (NC 010415)	S T T T A H G C D V I A R C N C N T G V Y F C R S K G K H Y P V C F Q G P G L T W I E A N E Y Y P A R Y Q S H V L L A P G P A E P G D C G G I L R C Q H G V I G L V T A G G N G I V A F A D L R D L L W
RV-A (NC 001617)	Y R T N T E G N D V I P N C D C T E C T Y Y C H H K D R Y F P I R V T A H D W Y E I Q E S E Y Y P K H I Q Y N L L I G E G P C E P G D C G G K L L C K H G V I G M I T A G G E G H V A F I D L R K F Q C
RV-B (NC 001490)	V S T G G H G A E T I P H C N R T S G V Y Y S T Y Y R K Y Y P I I C E K P T N I W I E G S P Y Y P S R F Q A G V M K G V G P A E L G D C G G I L R C I H G P I G L L T A E G S G Y V C F A D I R Q L E C
RV-C (NC 009996)	D A A D Q P G P D T I P Q C D C L E G C Y Y S K S L D R Y I P V K L E A H D W Y Q I E E T C Y Y P Q H I Q Y N I M I G E G H C Q P G D C G G K L L C T H G V I G I I T A G G D N H V A F T D L R P Y S C

		2B
AN12/Bos taurus/JPN/2014	I E D D V M E	Q G L T D Y V Q S L G N A F G A G F T D E I S N Y V G Q V R D M M V G S D T V V E K I V R N I I K L L S A L V I V V R N R S D I V T V T A T L S L L G C S G S P W R W L K A K I C S I L G
EV-E1 PA12-24791 (KC667561)	I E D D A M E	Q G I T D Y V Q Q L G N A F G A G F T A E I A N Y T N Q L K D M L V G S D S V V E K I I R S L V R L V S A L V I V V R N H Q D L V T V G A T L A L L G C E G S P W K W L K R K V C Q I L G
EV-E1 VG-5-27 (D00214)	I E D D A M E	Q G I T D Y V Q Q L G N A F G A G F T A E I A N Y T N Q L R D M L M G S D S V V E K I I R S L V R L V S A L V I V V R N H Q D L I T V G A T L A L L G C E G S P W K W L K R K V C Q I L G
EV-E1 LC-R4 (DQ092769)	I E D D A M E	Q G I T D Y V Q Q L G N A F G A G F T A E I A N Y T N Q L R D M L V G S D S V V E K I V R S L V R L V S A L V I V V R N H Q D L V T V G A T L A L L G C E G S P W K W L K R K V C Q I L G
EV-E1 Vir 404/03 (DQ092771)	I E D D A M E	Q G I T D Y V Q Q L G N A F G A G F T A E I A N Y T N Q L K D M L V G S D S V V E R I I R A L V R L V S A L V I V V R N H Q D L V T V G A T L A L L G C D G S P W K W L K R K V C Q T L G
EV-E2 PS83 (DQ092793)	I E D D A M E	Q G I T D Y V Q Q L G N A F G A G F T A E I A N Y T N Q L R D M L I G S D S V V E K I I R S L V R L I S A L V I V V R N H Q D L V T V G A T L A L L G C E G S P W K W L K R K T C Q I L G
EV-E2 PS42 (DQ092792)	I E D D A M E	Q G I T D Y V Q Q L G N A F G A G F T A E I A N Y T N Q L R D M L I G S D S V V E K I I R S L V R L I S A L V I V V R N H Q D L V T V G A T L A L L G C E G S P W K W L K R K T C Q I L G
EV-E2 SL305 (AF123433)	I E D D A M E	Q G I T D Y V Q Q L G N A F G A G F T A E I A N Y T N Q L R D M L V G S D S V V E K I I R S L V R L V S A L V I V V R N H Q D L V T V G A T L A L L G C E G S P W K W L K R K V C Q I L G
EV-E2 IS1/Bos_taurus/JPN/1990	I E D D A M E	Q G I T D Y V Q Q L G N A F G A G F T A E I A N Y T N Q L R D M L I G S D S V V E K I I R S L V R L V S A L V I V V R N H Q D L V T V G A T L A L L G C E G S P W K W L K R K V C Q I L G
EV-E2 K2577 (AF123432)	I E D D A M E	Q G I T D Y V Q Q L G N A F G A G F T A E I A N Y T N Q L R D M L V G S D S V V E K I I R S L V R L V S A L V I V V R N H Q D L V T V G A T L A L L G C E G S P W K W L K R K V C Q I L G
EV-E3 HY12 (KF748290)	I E D D A M E	Q G I T D Y V Q Q L G N A F G A G F T A E I A N Y T N Q L R D M L M G S D S V V E K I I R S L V R L V S A L V I V V R N H Q D L V T V G A T L A L L G C E G S P W K W L K R K V C Q T L G
EV-F1 BEV-261 (NC_021220)	I E D D V M E	Q G L T D N V Q S L G N A F G A G F T D E I S N Y V G Q V R D M M V G S D T V V E R V V G N I I K L L S A L V I V V R N R S D I I T V T A T L S L L G C S G S P W R W L K S K I C S I L G
EV-F1 IL/alpaca (KC748420)	I E D D A M E	Q G L T D Y V Q S L G N A F G A G F T D E I S N Y V G Q V R D M M V G S D T V V E K I V R N I I K L L S A L V I V V R N R A D I I T V T A T L S L L G C S G S P W R W L K S K I C S I L G
EV-F2 BHM26 (HQ917060)	I E D D V M E	Q G L T D Y V Q S L G N A F G A G F T D E I S N Y V G Q V R D M M V G S D T V V E K I V R N I I K L L S A L V I V V R N R S D I I T V T A T L S L L G C S G S P W R W L K A K I C S I L G
EV-F2 BJ001 (HQ663846)	I E D D A M E	Q G L T D Y V Q S L G N A F G A G F T D E I S N Y V G Q V R D M M V G S D T V V D K I V R N I I K L L S A L V I V V R N R A D I I T V T A T L S L L G C S G S P W R W L K S K I C S I L G
EV-F2 PS89 (DQ092795)	I E D D A M E	Q G L T D Y V Q S L G N A F G A G F T D E I S N Y I G Q V R D M M V G S D T V V E K I V R N I I K L L S A L V I V V R N R S D I I T V T A T L S L L G C S G S P W R W L K S K I C S I L G
EV-F2 3A (AY508697)	I E D D A M E	Q G L T D Y V Q S L G N A F G A G F T D E I S N Y V G Q V R D M M V G S D T V V E K I V R N I V K L L S A L V I V V R N R S D I I T V T A T L S L L G C S G S P W R W L K S K I C S I L G
Ho12/Bos taurus/JPN/2014	I E D D V M E	Q G L T D Y V Q S L G N A F G A G F T D E I S N Y V G Q V R D M M V G S D T V V E K I V R N I I K L L S A L V I V V R N R A D I I T V T A T L S L L G C S G S P W R W L K S K I C S I L G
IS2/Bos taurus/JPN/1990	I E D D V M E	Q G L T D Y V Q S L G N A F G A G F T D E I S N Y V G Q V R D M M V G S D T V V E K I V R N I I K L L S A L V I V V R N R S D I I T V T A T L S L L G C S G S P W R W L K S K I C S I L G
EV-F3 PS87/Belfast (DQ092794)	V E D D V M E	Q G L T D Y V Q S L G N A F G A G F T D E I S N Y V G Q V R D M M V G S D T V V E K I V R N I I K L L S A L V I V V R N R S D I I T V T A T L S L L G C S G S P W R W L K S K I C S I L G
EV-F4 Possum enterovirus W1 (AY462106)	I E D D V M E	Q G L T D Y V Q G L G N A F G A G F T N E I C N Y V N Q V K D M M I G S D T V V E K I I R N V I R L L S A L V I V V K N P S D I V T V T A T L S L L G C T G S P W R W L K A K I C S I L G
EV-F4 Possum enterovirus W6 (AY462107)	I E D D V M E	Q G F S D Y V Q S L G N A F G T G F T N E I S N Y V G Q V R D M M L G S D T V V E K I I R N V I K L L S A L V I V V K N Q S D I V T V T A T L S L L G C S G S P W R W L K S K I C S I L G
EV-A (NC 001612)	L D E E A M E	Q G V S D Y I K G L G D A F G T G F T D A V S R E V E A L K N H L I G S E G A V E K I L K N L I K L I S A L V I V I R S D Y D M V T L T A T L A L I G C H G S P W A W I K A K T A S I L G
EV-B (NC 001472)	L E D D A M E	Q G V K D Y V E Q L G N A F G S G F T N Q V C E Q V N L L K E S L V G Q D S I L E K S L K A L V K I V S A L V I V V R N H D D L I T V T A T L A L I G C T S S P W R W L K Q K V S Q Y Y G
EV-C (NC 002058)	Y E E E A M E	Q G I T N Y I E S L G A A F G S G F T Q Q I S D K I T E L T N M V T S - - T I T E K L L K N L I K I I S S L V I I T R N Y E D T T T V L A T L A L L G C D A S P W Q W L R K K A C D V L E
EV-D (NC 001430)	L E D D A M E	Q G I T D Y I Q N L G S A F G T G F T E T I S E K A K E I Q N M L V G E D S L L E K L L K A L I K I V S A M V I V I R N S E D L V T V T A T L A L L G C N D S P W A F L K Q K V C S Y L G
EV-G (NC 004441)	V E H E A M E	Q G L T D Y I Q Q L G N S F G Q G F T A E I T N Y A S Q L S E M L I G A D G M V E R C L Q T F V K V I S A V V I A T R S Q G D V P T I L A T L A L I G C D G S P W R W L K R Q F C G I F K
EV-H (NC 003988)	V E D D V M E	Q G V A D Y V K Q L G N A F G A G F T D E I S S Y A Q Q I K N M F V G Q D S L V E K M I K N L V K I V C A L V I L I R N S S D I T T V T A T L A L L G C S T S P W K W L K T K I S Q L C G
EV-J (NC 010415)	L E D D A M E	Q G I T D Y V A Q L G Q A F G S G F T Q E I A D K T Q Q L K D M L I G Q D S I L E K C L K A L I K I V S A L V I I V R N H E D L I T V T A T L A L I G C S G S P W Q W L K T K V C Q Y L Q
RV-A (NC 001617)	A E - - - E Q	G - L S D Y V E H L G Q V F G V G F V D S I K Q Q V N F I N - - P - - T S K I G S K V I K W L L R I V S A M I I M V R N S S D P Q T V I A T L T L L G C S G S P W R F L K E K L C A W L Q
RV-B (NC 001490)	I A E - - - E	Q G L S D Y I T G L G R A F G V G F T D Q I S T K V T E L Q - - E V A K D F L T T K V L S K V V K M V S A L V I I C R N H D D L V T V T A T L A L L G C D G S P W R F L K M Y I S K H F Q
RV-C (NC 009996)	L D T - - H Q	G P I Q D Y V T Q L G S A F G S G F T D T L K D Q L T D V S - - A H I K D K I T S K V V K W L V R I I S A L T I M I R N S T D T P T V L A T L A L L G C H K S P W S F L K D R L C Q W L N

Table S2. Multiple alignments result using amino acid sequences of polyprotein (continued)

2C (marked above column 9, where the 2C region begins)

Sequence	Alignment
AN12/Bos taurus/JPN/2014	IN-MAQKQA-DGWIKKFTEAVNAFKGLDWIAAKFSKFLDWIKSKIIPELRERAEFVKNLKQLPLLEAQINTLEHSNPNQETQEQLFSNVQYLAHHCRKNA
EV-E1 PA12-24791 (KC667561)	IN-MAERQS-DNWMKKFTEMCNAFRGLDWIAAKISKFIDWLKQKILPELKERAEFVKNLKQLPLLEAQVNTLEHSAASQEKQEQLFGNIQYLAHHCRKNA
EV-E1 VG-5-27 (D00214)	IN-MAERQS-DNWMKKFTEMCNAFRGLDWIAAKISKFIDWLKQKILPELKERAEFVKKLKQLPLLEAQVNTLEHSSASQERQEQLFGNVQYLAHHCRKNA
EV-E1 LC-R4 (DQ092769)	IN-MAERQS-DNWMKKFTEMCYAFRGLDWIATKISKFIDWLKQKILPELKERAEFVKNLKQLPLLEAQVNTLEHSSASQEKQEQLFGNVQYLAHHCRKNA
EV-E1 Vir 404/03 (DQ092771)	IN-MAERQS-DNWMKKFTEMCNAFRGLDWIAAKISKFIDWLKQKILPELKERAEFVKNLKQLPLLEAQVNTLEHSSASQEKQEQLFGNIQYLAHHCRKNA
EV-E2 PS83 (DQ092793)	IN-MAERQS-DSWMKKFTEMCNAFRGLDWIAAKISKFIDWLKQKILPELKERAEFLKNLKQLPLLEAQVNTLEHSSASQEKQELLFGNIQYLAHHCRKNA
EV-E2 PS42 (DQ092792)	IN-MAERQS-DSWMKKFTEMCNAFRGLDWIAAKMSKFIDWLKQKILPELKERAEFLKNLKQLPLLEAQVNTLEHSSASQEKQELLFGNIQYLAHHCRKNA
EV-E2 SL305 (AF123433)	IN-MAEKQS-DNWMKKFTEMCNAFRGLDWIAAKISKFIDWLKQKILPELKERAEFVKNLKQLPLLEAQVNTLEHSSASQEKQEQLFGNIQYLAHHCRKNA
EV-E2 IS1/Bos_taurus/JPN/1990	IN-MAERQS-DNWMKKFTEMCNAFRGLDWIAAKISKFIDWLKQKILPELKERAEFVKNLKQLPLLEAQVNTLEHSSASQEKQEQLFGNIQYLAHHCRKNA
EV-E2 K2577 (AF123432)	IN-MAERQS-DNWMKKFTEMCNAFRGLDWIAAKISKFIDWLKQKILPELKERAEFVKNLKQLPLLEAQVNTLEHSSASQEKQEQLFGNVQYLAHHCRKNA
EV-E3 HY12 (KF748290)	VN-MAERQS-DNWIKKFTEMCNAFRGLDWIAVKISKFIDWLKQKILPELKERAEFVKNLRQLPLLEAQVNTLEHSSASQEKQEQLFGNVQYLAHHCRKNA
EV-F1 BEV-261 (NC_021220)	IN-MAQKQA-DGWVKKFTEAVNAFKGLDWIAAKLSKFLDWIKSKIIPELRERAEFVKNLRQLPLLEAQITTLEHSNPNQETQEQLFSNVQYLAHHCRKNA
EV-F1 IL/alpaca (KC748420)	IN-MAQKQA-DSWIKKFTEAVNAFKGLDWIAAKFSKFLDWIKSKIIPELRERTEFVKNLRQLPLLEAQITTLEHSNPNQETQETLFSNVQYLAHHCRKNA
EV-F2 BHM26 (HQ917060)	IN-MAQKQA-DSWIKKFTEAVNAFKGLDWIAAKFSKFLDWIKSKIIPELRERTEFVKHLRQLPLLEAQINTLEHSNPNQETQEQLFSNVQYLAHHCRKNA
EV-F2 BJ001 (HQ663846)	IN-MAQKQA-DSWIKKFTEAVNAFKGLDWIAAKFSRFLDWIKSKIIPELRERTEFVKNLRQLPLLEAQIATLEHSNPNQETQETLFSNVQYLAHHCRKNA
EV-F2 PS89 (DQ092795)	IN-MAQKQA-DSWVKKFTEAVNAFKGLDWIAAKFSKFLDWIKSKIIPELRERTEFVKNLRQLPLLEAQINTLEHSNPNQETQEQLFSNIQYLAHHCRKNA
EV-F2 3A (AY508697)	IN-MAQKQA-DSWIKKFTEAVNAFKGLDWIAAKFSKFLDWIKSKIIPELRERTEFVKNLRQLPLLEAQINTLEHSNPNQETQEQLFSNVQYLAHHCRKNA
Ho12/Bos taurus/JPN/2014	IN-MAQKQA-DGWVKKFTEAVNAFKGLDWIAAKFSKFLDWIKSKIIPELRERTEFVKNLRQLPLLEAQIATLEHSNPNQETQEQLFSNVQYLAHHCRKNA
IS2/Bos taurus/JPN/1990	IN-MAQKQA-DSWVKKFTEAVNAFKGLDWIAAKFSKFLDWIKSKIIPELRERTEFVKNLRQLPLLEAQINTLEHSNPNQETQEQLFSNVQYLAHHCRKNA
EV-F3 PS87/Belfast (DQ092794)	IN-MAQKQA-DSWIKKFTEAVNAFKGLDWIAAKFSKFLDWIKSKIIPELKERTEFVKNLRQLPLLEAQINTLEHSNPNQEAQEQLFSNVQYLAHHCRKNA
EV-F4 Possum enterovirus W1 (AY462106)	IN-MAQKQA-DSWIKKFTEAVNAFKGLDWIAAKFSKFLDWIKSKIIPELKERTEFLKNLRQLPLLEAQIATLEHSNPTQETQEILFSNVQYLAHHCRKNA
EV-F4 Possum enterovirus W6 (AY462107)	IN-MAQKQA-DSWIKKFTEAVNAFKGLDWIAAKFSKFLDWIKSKIIPELKERTEFLKNLKQLPLLEAQIATLEHSNPNQEAQETLFSNVQYLAHHCRRNA
EV-A (NC 001612)	IP-IAQKQS-ASWLKKFNDMANAAKGLEWISNKISKFIDWLKEKIIPAAKEKVEFLNNLKQLPLLENQISNLEQSAASQEDLEAMFGNVSYLAHFCRKFQ
EV-B (NC 001472)	IP-MAERQN-SGWLKKFTEMTNACKGMEWIAIKIQKFIEWLKVKILPEVKEKHEFLNRLKQLPLLESQIATIEQSAPSQSDQEQLFSNVQYFAHYCRKYA
EV-C (NC 002058)	IP-YVIKQG-DSWLKKFTEACNAAKGLEWVSNKISKFIDWLKEKIIPQARDKLEFVTKLRQLEMLENQISTIHQSCPSQEHQEILFNNVRWLSIQSKRFA
EV-D (NC 001430)	IP-YTIRQS-DSWLKKFTEACNALRGLDWLAQKIDKFINWLKTKILPEAREKHEFVQKLKQLPVIESQINTIEHSCPNSEXQQALFNNVQYYSHYCKYA
EV-G (NC 004441)	IP-YVEKQG-DDWLKKFTSYVNAFKGLDWVAEKIMKFIDWMKNKLIPQARERQEFTTNLKTLPLLEAQVATLEHSCPTTEQQETIFGNIQYLAHHCRRYA
EV-H (NC 003988)	VT-IAERQS-DSWLKKFIEFTNAFKGLEWLAAKIEKFIEWVKMKILPAVRERADFVNRLKQLPLLEAQMATLEHAAPNQEKQERLFTNVQYFATYCRKYA
EV-J (NC 010415)	IP-VVEKQG-DSWLKKFTEATNAAKGLEWIAQKISKFIDWLKTKIVPEVKEKVEFLGKLKQLPLLESQITSIEHSAPSQDDQERLFGNVQYLAHYCRRYA
RV-A (NC 001617)	LS-YVHKQS-DSWLKKFTEACNAARGLEWIGQKISKFIDWIKS-MLPQAQLKIDYLTKLKQLNLLEKQIETIRLAPAS--VQEKIFIEINTLHDLSLKFL
RV-B (NC 001490)	VP-YIERQANDGWFRKFNDACNAAKGLEWIANKISKLIEWIKNKVLPQAKEKLEFCSKLKQLDILERQITTMHISNPTQEKREQLFNNVLWLEQMSQKFA
RV-C (NC 009996)	ITRPPSRQG-DGWLKKFTECCNAAKGLEWIGIKISKFIEWLKNRLLPTLQHKKEILDECKKIDLYQEQVKGFSKVDEP--AQQELLIKVEKLKKGLDSLA

Sequence	Alignment
AN12/Bos taurus/JPN/2014	PLYAAEARRVYALEKRVLGAMQFKTKNRIEPVCCLIHGTPGTGKSLATTIIGRKIAEYENSGVYSLPPDPDHFDGYQEQAVVIMDDLHQNPDGKDMSLFC
EV-E1 PA12-24791 (KC667561)	PFYAAEAKRVYHLEKRVLGAMQFKTKNRIEPVCALIHGSPGTGKSLATMIVGRKLAEYEGSDVYSLPPDPDHFDGYQQQAVVVMDDLLQNPDGKDMTLFC
EV-E1 VG-5-27 (D00214)	PLYAAEAKRVYHLEKRVLRAMQFKTKNRIEPVCALIHGSPGTGQSLATMIVGRKLAEYEGSDVYSLPPDPDHFDGYQQQAVVVMDDLLQNPDGKDMTLFC
EV-E1 LC-R4 (DQ092769)	PLYAAEAKRVYHLEKRVLGAMQFKTKNRIEPVCALIHGSPGTGKSLATMIVGRKLAEYEGSDVYSLPPDPDHFDGYQQQAVVVMDDLLQNPDGKDMTLFC
EV-E1 Vir 404/03 (DQ092771)	PLYAAEAKRVYHLEKRVLGAMQFKTKNRIEPVCALIHGSPGTGKSLATMIVGRKLAEYEGSDVYSLPPDPDHFDGYQQQAVVVMDDLLQNPDGKDMTLFC
EV-E2 PS83 (DQ092793)	PLYAAEAKRVYHLEKRVLGAMQFKTKNRIEPVCALIHGSPGTGKSLATMIVGRKLAEYESSDVYSLPPDPDHFDGYQQQAVVVMDDLLQNPDGKDMTLFC
EV-E2 PS42 (DQ092792)	PLYAAEAKRVYHLEKRVLGAMQFKTKNRIEPVCALIHGSPGTGKSLATMIVGRKLAEYESSDVYSLPPDPDHFDGYQQQAVVIMDDLLQNPDGKDMTLFC
EV-E2 SL305 (AF123433)	PLYAAEAKRVYHLEKRVLGAMQFKTKNRIEPVCALIHGSPGTGKSLATMIVGRKLAEYEGSDVYSLPPDPDHFDGYQQQAVVVMDDLLQNPDGKDMTLFC
EV-E2 IS1/Bos_taurus/JPN/1990	PLYAAEAKRVYHLEKRVLGAMQFKTKNRIEPVCALIHGSPGTGKSLATMIVGRKLAEYEGSDVYSLPPDPDHFDGYQQQAVVVMDDLLQNPDGKDMTLFC
EV-E2 K2577 (AF123432)	PLYAAEAKRVYHLEKRVLGAMQFKTKNRIEPVCALIHGSPGTGKSLATMIVGRKLAEYEGSDVYSLPPDPDHFDGYQQQAVVVMDDLLQNPDGKDMTLFC
EV-E3 HY12 (KF748290)	PLYAAEAKRVYHLEKRVLGAMQFKTKNRIEPVCALIHGSPGTGKSLATMIVGRKLAEYESSDVYSLPPDPDHFDGYQQQAVVVMDDLLQNPDGKDMTLFC
EV-F1 BEV-261 (NC_021220)	PLYAAEARRVFALEKRVLGAMQFKTKNRIEPVCCLIHGTPGTGKSLATTIIGRKIAEYENSGVYSLPPDPDHFDGYAEQAVVIMDDLHQNPDGKDMSLFC
EV-F1 IL/alpaca (KC748420)	PLYAAEARRVFALEKRVLGAMQFKTKNRIEPVCCLIHGTPGTGKSLATTIIGRKIAEYENSGVYSLPPDPDHFDGYQEQAVVIMDDLHQNPDGKDMSLFC
EV-F2 BHM26 (HQ917060)	PLYAAEARRVFALEKRVLGAMQFKTKNRIEPVCCLIHGTPGTGKSLATTIIGRKIAEYENSGVYSLPPDPDHFDGYAEQAVVIMDDLHQNPDGKDMSLFC
EV-F2 BJ001 (HQ663846)	PLYAAEARRVFALEKRVLGAMQFKTKNRIEPVCCLIHGTPGTGKSLATTIIGRKIAEYENSGVYSLPPDPDHFDGYQEQAVVIMDDLHQNPDGKDMSLFC
EV-F2 PS89 (DQ092795)	PLYAAEARRVFALEKRVLGAMQFKTKNRIEPVCCLIHGTPGTGKSLATTIIGRKIAEYENSGVYSLPPDPDHFDGYAEQAVVIMDDLHQNPDGKDMSLFC
EV-F2 3A (AY508697)	PLYAAEARRVFALEKRVLGAMQFKTTNRLEPVCCLIHGTPGTGKSLATTIIGRKIAEYENSGVYSLPPDPDHFDGYAEQAVVIMDDLHQNPDGKDMSLFC
Ho12/Bos taurus/JPN/2014	PLYAAEARRVYALEKRVLGAMQFKTKNRIEPVCCLIHGTPGTGKSLATTIIGRKIAEYENSGVYSLPPDPDHFDGYQEQAVVIMDDLHQNPDGKDMSLFC
IS2/Bos taurus/JPN/1990	PLYAAEARRVYALEKRVLGAMQFKTKNRIEPVCCLIHGTPGTGKSLATTIIGRKIAEYENSGVYSLPPDPDHFDGYQEQAVVIMDDLHQNPDGKDMSLFC
EV-F3 PS87/Belfast (DQ092794)	PLYAAEARRVFALEKRVLGAMQFKTKNRIEPVCCLIHGTPGTGKSLATTVIGRKIAEYENSCVYSLPPDPDHFDGYAEQAVVIMDDLHQNPDGKDMSLFC
EV-F4 Possum enterovirus W1 (AY462106)	PLYAAEARRVFALEKRVLGAMQFKTKNRIEPVCCLIHGSPGTGKSLATSIIGRRIAEYENSGVYSLPPDPNHFDGYQEQAVVVMDDLLQNPDGKDMSLFC
EV-F4 Possum enterovirus W6 (AY462107)	PLYAAEARRVFALEKRVLGAMQFKTKHRIEPVCCLIHGSPGTGKSLATTIIGRKIADYEGSGVYSLPPDPDHFDGYQEQAVVIMDDLHQNPDGKDMSLFC
EV-A (NC 001612)	PLYATEAKRVYALEKRMNNYMQFKSKHRIEPVCLIIRGSPGTGKSLATGIIARAIADKYHSSVYSLPPDPDHFDGYKQQVVTVMDDLCQNPDGKDMSLFC
EV-B (NC 001472)	PLYAAEAKRVFSLEKKMSNYIQFKSKCRIEPVCLLLHGSPGAGKSVATNLIGRSLAEKLNSSVYSLPPDPDHFDGYKQQAVVIMDDLCQNPDGKDVSLFC
EV-C (NC 002058)	PLYAVEAKRIQKLEHTINNYIQFKSKHRIEPVCLLVHGSPGTGKSVATNLIARAIAERENTSTYSLPPDPSHFDGYKQQGVVIMDDLNQNPDGADMKLFC
EV-D (NC 001430)	PLYALEAKRVSALERKINNYIQFKSKSRIEPVCLIIHGSPGTGKSVASNLIARAITEKLGGDSYSLPPDPKYFDGYKQQTVVLMDDLMQNPDGNDIAMFC
EV-G (NC 004441)	PLYAAEARRVYALEKRILGYIQFKSKQRIEPVCLLIHGTAGTGKSLATSIIGRKLAEYEHSEVYAIPPDSDHFDGYQQQAVVVMDDLNQNPDGKDMVAFC
EV-H (NC 003988)	PLYASEARRVFQMEKKMNNYIQFKSKSRIEPVCLLIHGSPGAGKSVATSIIGRAIAESISGSVYSLPPDPTFFDGYKQQEVVIMDDLLQNPDGKDVGMLC
EV-J (NC 010415)	PLYAAEARRVFTLEKKMSNYMQFKTKSRIEPVCLLIHGTPGAGKSVASSLIGRAIAEKLNSCVYSLPPDPDHFDGYKQQAVVIMDDLCQNPDGKDVSLLC
RV-A (NC 001617)	PLYASEARRIKNLY--IKCSNVIKGGKRNEPVAVLIHGSPGTGKSLATSVLARMLTV--ETDIYSLPPDPKYFDGYDQQSVVIMDDIMQNPSGEDMTLFC
RV-B (NC 001490)	PFYAVESKRIRELKNKMVNYMQFKSKQRTEPVCVLIHGTPGSGKSLTTSIVGRAIAEHFNSAVYSLPPDPKHFDGYQQQEVVIMDDLNQNPDGQDISMFC
RV-C (NC 009996)	PLYAVENKRVTNIYKELKALSAYKTTQRTEPVCILIRGAPGCGKSLVTSIIARGLTK--PGDIYSLPPDPKHFDGYDQQEVVIMDDLGQNPDGKDLSMFC

Table S2. Multiple alignments result using amino acid sequences of polyprotein (continued)

Strain	Sequence
AN12/Bos taurus/JPN/2014	QMVSTTPFVVPMAALEDKGRLFTSKYVLASTNANHIHPVTVADGKALQRRFHFDTDIELMDGYVRNGK-LDIQKATEACDD--CSPINFQKCMPLICGKA
EV-E1 PA12-24791 (KC667561)	QMVSTAPFTVPMAALEDKGKLFTSKFVLASTNAGQVTPPTVADYKALQRRFFFDCDIEVQKEYRRNGATLDVAKATETCDD--CSPVNFKKCMPLICGKA
EV-E1 VG-5-27 (D00214)	QMVSTAPFTVPMAALEDKGKLFTSKFVLASTNAGQVTPPTVADYKALQRRFFFDCDIEVQKEYKRDGVTLDVAKATETCED--CSPANFKKCMPLICGKA
EV-E1 LC-R4 (DQ092769)	QMVSTAPFTVPMAALEDKGKLFTSKFVLASTNAGQVTPPTVADYKALQRRFFFDCDIEVQKDYKRNGTTLDVAKATETCED--CSPVNFKKCMPLICGKA
EV-E1 Vir 404/03 (DQ092771)	QMVSTAPFTVPMAALEDKGKLFTSKFVLASTNAGQVTPPTVADYKALQRRFFFDCDIEVQKEYRKNGTTLDVAKATEACED--CVPVNFKKCMPLICGKA
EV-E2 PS83 (DQ092793)	QMVSTAPFTVPMAALEDKGKLFTSKFVLASTNAGQVTPPTVADYKALQRRFFFDCDIEVQKEYRKNGTTLDVAKATEACED--CSPVNFKKCMPLICGKA
EV-E2 PS42 (DQ092792)	QMVSTAPFTVPMAALEDKGKLFTSKFVLASTNAGQVTPPTVADYKALQRRFFFDCDIEVQKEYRKNGTTLDVAKATEACED--CSPVNFKKCMPLICGKA
EV-E2 SL305 (AF123433)	QMVSTAPFTVPMAALEDKGKLFTSKFVLASTNAGQVTPPTVADYKALQRRFFFDCDIEVQKDYKRNGVTLDVAKATETCED--CSPANFKKCMPLICGKA
EV-E2 IS1/Bos_taurus/JPN/1990	QMVSTAPFTVPMAALEDKGKLFTSKFVLASTNAGQVTPPTVADYKALQRRFFFDCDIEVQKEYRRNGTTLDVAKATETCDD--CSPANFKKCMPLICGKA
EV-E2 K2577 (AF123432)	QMVSTAPFTVPMAALEDKGKLFTSKFVLASTNAGQVTPPTVADYKALQRRFFFDCDIEVQKDYKKNGVTLDVAKATETCED--CSPANFKKCMPLICGKA
EV-E3 HY12 (KF748290)	QMVSTAPFTVPMAALEDKGKLFTSKFVLASTNAGQVTPPTVADYKALQRRFYFDCDIEVQKDYKKNGVTLDVAKATEVCED--CSPVNFKKCMPLICGKA
EV-F1 BEV-261 (NC_021220)	QMVSTTPFVVPMAALEDKGRLFTSKYVLASTNANHIHPVTVADGKALQRRFHFDTDIELMDGFVKNGK-LDIQRATEACED--CSPINFQKCMPLICGKA
EV-F1 IL/alpaca (KC748420)	QMVSTTPFVVPMAALEDKGRLFTSKYVLASTNANHIHPVTVADGKALQRRFHFDTDIEIMDGYVQNGK-LNIQKATESCED--CSPINFQKCMPLICGKA
EV-F2 BHM26 (HQ917060)	QMVSTTPFVVPMAALEDKGRLFTSKYVLASTNANHIHPVTVADGKALQRRFHFDTDIELMDGFVKNGK-LDIQKATEACDD--CSPINFQKCMPLICGKA
EV-F2 BJ001 (HQ663846)	QMVSTTPFVVPMAALEDKGRLFTSKYVLASTNANHIHPVTVADGKALQRRFHFDTDIEIMDGYVQNGK-LNIQKATESCED--CSPINFQKCMPLICGKA
EV-F2 PS89 (DQ092795)	QMVSTTPFVVPMAALEDKGRLFTSKYVLASTNANHIHPVTVADGKALQRRFHFDTDIELMDSYVKNGK-LDIQKATEACED--CSPINFQKCMPLICGRA
EV-F2 3A (AY508697)	QMVSTTPFVVPMAALEDKGRLFTSKYVLASTNANHIHPVTVADGKALQRRFHFDTDIELMDGYVKNGK-LDIQKATEACDD--CSPINFQKCMPLVCGKA
Ho12/Bos taurus/JPN/2014	QMVSTTPFVVPMAALEDKGRLFTSKYVLASTNANHIHPVTVADGKALQRRFHFDTDIELMDGFVKNGK-LDIQKATEACDD--CSPINFQKCMPLICGKA
IS2/Bos taurus/JPN/1990	QMVSTTPFVVPMAALEDKGRLFTSKYVLASTNANHIHPVTVADGKALQRRFHFDTDIELMDGYVKNGK-LDIQKATETCDD--CSPINFQKCMPLICGKA
EV-F3 PS87/Belfast (DQ092794)	QMVSTTPFVVPMAALEDKGRLFTSKYVLASTNANHIHPVTVADGKALQRRFHFDTDIELMDGYVKNGK-LDIQKATEACDD--CSPINFQKCMPLICGKA
EV-F4 Possum enterovirus W1 (AY462106)	QMVSTVPFVVPMAALEDKGRLFTSKYVLASTNANTMHPVTVADGRALQRRFHFDTVIEIMPDYSDNGK-LNVAKATEQCDD--CSPINFKKCMPLICGKA
EV-F4 Possum enterovirus W6 (AY462107)	QMVSTAPFVVPMAALEDKGRLFTSKYVLASTNANTMHPVTVADGKALQRRFHFDTDIEIMKDYVDNGK-LDIKKATETCED--CSPINFQKCMPLICGKA
EV-A (NC 001612)	QMVSTVDFIPPMASLEEKGVSFTSKFVIASTNASNIIVPTVSDSDAIRRRFYMDCDIEVTDSYKTDLGRLDAGRAARLCSE--NNTANFKRCSPLVCGKA
EV-B (NC 001472)	QMVSSVDFVPPMAALEEKGILFTSPFVLASTNAGSINAPTVSDSRALARRFHFDMNIEVISMYSQNGK-INMPMSVKTCDEE-CCPVNFKKCCPLVCGKA
EV-C (NC 002058)	QMVSTVEFIPPMASLEEKGILFTSNYVLASTNSSRISPPTVAHSDALARRFAFDMDIQVMNEYSRDGK-LNMAMATEMCKN-CHQPANFKRCCPLVCGKA
EV-D (NC 001430)	QMVSTVDFIPPMASLEEKGTLYTSPFLIATTNAGSIHAPTVSDSKALARRFKFDMEIESMESYKDGVR-LDMFKAVELCNPEKCRPTNYKKCCPLICGKA
EV-G (NC 004441)	QMVSTVPYHVPMAAIEEKGMLFTSSYVLASTNSGSIHPPTVSNSKALSRRFAFDVDIEVSEHYKTHNGTLDVVNATQRCED--CCPANFKTCMPLICGEA
EV-H (NC 003988)	QMVSTVDFHPPMAALEEKGMLYTSPFLLASTNASSIHAPTISDSKALNRRFHFDVNIEIMDQYKKEGR-INMPEAARPCD-EECYPCNFKRCCPLVCGKA
EV-J (NC 010415)	QMVSTVDFIPPMASLEEKGILYTSPFMIASTNHGSITAPTISDSRALNRRFYLDVDIAINDEYKVNNK-VDMQRACQHCHN--CDPANFKRCCPLICGRA
RV-A (NC 001617)	QMVSSVPFIPPMADLPDKGKPFTSKFVLASTNHTLLTPPTVSSLPAMARRFYFDLDIQVKKEYL-LDGKLDIAKSFRPCDVN-IKIGN-AKCCPFICGKA
RV-B (NC 001490)	QMVSSVDFLPPMASLDNKGMLFTSNFVLASTNSNTLSPPTILNPEALVRRFGFDLDICLHTTYTKNGK-LNAGMSTKTCKD-CHQPSNFKKCCPLVCGKA
RV-C (NC 009996)	QMVSTTNFIPPMAALEDKGKTFTSKYILASTNLLNLQPPTVTVPEAIDRRFYLDLDLKILQGYQNQVGLLDTAKALQPCANC-SKPPHYKQCCPLLCGKA

P3 protein
3A

Strain	Sequence
AN12/Bos taurus/JPN/2014	LQLRSKKGDGMRYSIDTMITEMRRESARRYNIGNVIEALFQGPPEFRPLRIDVSEETPAPPAIADLLASVDSEDVREYCRQKGWIVQERITKESLERNVS
EV-E1 PA12-24791 (KC667561)	LQLKSRKGDGMRYSLDTLISELRRESNRRYNIGSVLEALFQGPVSYKPLRIEVIEEEPAPSAISDLLQAVDNEEVREYCRSKGWIVEEKVTELKLERNVN
EV-E1 VG-5-27 (D00214)	LQLKSRKGDGMRYSLDTLISELRRESNRRYNIGNVLEALFQGPVCYKPLRIEVHEEEPAPSAISDLLQAVDSEEVREYCRSKGWIVEERVTELKLERNVN
EV-E1 LC-R4 (DQ092769)	LQLKSRKGDGMRYSLDTLISELRRESNRRYNIGNVLEALFQGPVCYKPLRIEVHEEEPAPSAISDLLQAVDSEEVREYCRSKGWIVEEKVTELKLERNVN
EV-E1 Vir 404/03 (DQ092771)	LQLKSRKGDGMRYSLDTLISELRRESNRRFNIGNVLEALFQGPVCYKPLRIEVCEEQPAPSAISDLLQAVDSEEVREYCRSKGWIVEEKVTEMRLERNVN
EV-E2 PS83 (DQ092793)	LQLKSRKGDGMRYSLDTLISELRRESNRRYNIGNVLEALFQGPVCYKPLRIEVHEEEPAPSAISDLLQAVDSEEVREYCRSKGWIIQEKVTEHTLERNVN
EV-E2 PS42 (DQ092792)	LQLKSRKGDGMRYSLDTLISELRRESNRRYNIGNVLEALFQGPVCYKPLRIEVHEEEPAPSAISDLLQAVDSEEVREYCRSKGWIIQEKVTEHTLERNVN
EV-E2 SL305 (AF123433)	LQLKSRKGDGMRYSLDTLISELRRESNRRYNIGNVLEALFQGPVSYKPLRIEVIEEEPAPSAISDLLQAVDNEEVREYCRSKGWIIEEKVTELKLERNVN
EV-E2 IS1/Bos_taurus/JPN/1990	IQLKSRKGDGMRYSLDTLISELRRESNRRYNIGNVLEALFQGPVSYKPLRIEVIEEEPAPSAISDLLQAVDSEEVREYCRSKGWIVEEKVTEMKLERNVN
EV-E2 K2577 (AF123432)	LQLKSRKGDGMRYSLDTLISELRRESNRRHNIGNVLEALFQGPACYKPLRIEVHEEEPAPSAISDLLQAVDSEEVREYCRSKGWIVVEKVTELKLERNVN
EV-E3 HY12 (KF748290)	LQLKSRKGDGMRYSLDTLISELRRESNRRHNIGNVLEALFQGPVTYKPLKIEVIEEEPAPSAISDLLQAVDNEEVREYCRSKGWIVEEKVTELRLERNVN
EV-F1 BEV-261 (NC_021220)	LQLRSKKGDGMRYSIDTMITEMRRESARRYNIGNVIEALFQGPPEFKPLRIDVSEETPAPPAIADLLASIDSEEVREYCRIKGWIVQEKITKESLERNVN
EV-F1 IL/alpaca (KC748420)	LQLRSKKGDGMRYSIDTMITEMRRESARRYNIGNVIEALFQGPPEFKPLRIDVSEETPAPPAIADLLASVDSEDVREYCRQKGWIVQEKITKESLERNVN
EV-F2 BHM26 (HQ917060)	LQLRSKKGDGMRYSIDTMITEMRRESARRYNIGNVIEALFQGPPEFKPLRIDVSEETPAPPAIADLLASVDSEDVREYCRSKGWIVQERITKESLERNVN
EV-F2 BJ001 (HQ663846)	LQLRSKKGDGMRYSIDTMITEMRRESARRYNIGNVIEALFQGPPEFKPLRIDVSEETPAPPAIADLLASVDSEEVREYCRTKGWIVQEKVTKESLERNVN
EV-F2 PS89 (DQ092795)	LQLRSKKGDGMRYSIDTMITEMRRESARRYNIGNVIEALFQGPPEFKPLRIDVSEETPAPPAIADLLASVDSEEVRDYCRTKGWIVQEKITKESLERNVN
EV-F2 3A (AY508697)	LQLRSKKGDGMRYSIDTMITEMRRESARRYNIGNVIEALFQGPPEFKPLRIDVSEETPAPPAIADLLASVDSEDVREYCRQKGWIVQEKITKESLERNVN
Ho12/Bos taurus/JPN/2014	LQLRSKKGDGMRYSIDTMITEMRRESARRYNIGNVIEALFQGPPEFRPLRIDVSEETPAPPAIADLLASVDSEDVREYCRQKGWIVQEKITKESLERNVN
IS2/Bos taurus/JPN/1990	LQLRSKKGDGMRYSIDTMITEMRRESARRYNIGNVIEALFQGPPEFKPLRIDVSEETPAPPAIADLLASVDSEDVREYCRQKGWIVQEKITKESLERNVN
EV-F3 PS87/Belfast (DQ092794)	LQLRSKKGDGMRYSIDTMITEMRRESARRYNIGNVIEALFQGPPEFKPLRIDVSEETPAPPAIADLLASVDSEDVREYCRQKGWIVQEKITKESLERNVN
EV-F4 Possum enterovirus W1 (AY462106)	LQLRSKNGDGMRYSIDTMITEMRRESARRYNIGNVIEALFQGPPAYKPLRIDVSEETPAPPAIADLLSSVDSEEVREYCRQKGWIVQEKITKERLERNVN
EV-F4 Possum enterovirus W6 (AY462107)	LQLRSKKGDGMRYSIDTMITEMRRESARRYNIGNVIEALFQGPPVYKPLRIDISEETPAPPAIADLLSSVDSEDVREYCRQKGWIVQEKITKERLERNVN
EV-A (NC 001612)	IQLRDRK-SKVRYSVDTVVSELIREYNNRYAIGNTIEALFQGPPKFRPIRISLE-EKPAPDAISDLLASVDSEEVRQYCRDQGWIIPETP--TNVERHLN
EV-B (NC 001472)	IQFIDRR-TQVRYSLDMLVTEMFREYNHRHSVGATLEALFQGPPIYREIKISIAPETPPPPAIADLLKSVDSEAVREYCKEKGWLVPEINSTLQIEKHVS
EV-C (NC 002058)	IQLMDKS-SRVRYSIDQITTMIINERNRRSNIGNCMEALFQGPLQYKDLKIDIK-TSPPPECINDLLQAVDSQEVRDYCEKKGWIVNIT-SQVQTERNIN
EV-D (NC 001430)	IQFRDKR-TNVRYSVDMLVTEMIKEYRIRNSTQDKLEALFQGPPTFKEIKISVTPETPAPDAINDLLRSIDSQEVRDYCQKKGWIVMHPPTELVVDKHIS
EV-G (NC 004441)	YQLVDRR-NGMRYSIDTMISAMRAEWKRRNQVGLCYVRLFQGPPQFKPLKISVDPEIPAPPAIADLLASVDSEEVREYCKKKGWIVEVPVTATTLERNVS
EV-H (NC 003988)	IQLVDRR-LGIRYSIDMMVSELLREFTHRHNTQNLVEALFQ-GPIYHDLTITVE-ETPAPSAISDLLCSVDSGEVRDYCRRRGWIVPDTPTEITVSRDYG
EV-J (NC 010415)	IQFIDKR-SGVRYTLDMLVTEMFREYTRRNSIGNVLEALFQGPPIYRDIKISVAPETPAPPAIADLLKSVDSEEVRQYCQEKGWIIPVT--ECQTERHLS
RV-A (NC 001617)	VEFKDRN-SCTTLSLSQLYSHIKEEDRRRSSAAQAMEAIFQ--------GIDLQ-SPPPP-AIADLLRSVKTPEIIKYCQDNNWIVPAECS---IERDLG
RV-B (NC 001490)	ISLVDRT-TNVRYSVDQLVTAIISDFKSKMQITDSLETLFQ-GPVYKDLEIDVC-NTPPSECINDLLKSVDSEEIREYCKKKKWIIPEIPTN--IERAMN
RV-C (NC 009996)	VVLVNRR-TKGSYAINMVVQQLIEESKSRKSVGNNLTAIFQ--------GLGDK-ETPG--FIIDLLSSSKDPKVIQYCQDQGYISPVHGS---IERDFA

Table S2. Multiple alignments result using amino acid sequences of polyprotein (continued)

Strain		3B	3C
AN12/Bos taurus/JPN/2014	RALIVLQSATLIATICGVIYVVYKLFAGLQ	GPYSGIHTNYQKVKPVVRQVTTQ	GPLLDFAMSLLKKNIRTVVTKTGEFTGLGVYDTFMVLPRHAMAHGEV
EV-E1 PA12-24791 (KC667561)	RALAVIQSVSLIAAVAGTIYIVYRLFSGMQ	GPYSGIGSNYATKKPVVRQVQTQ	GPLFDFGVSLLKKNIRTVKTSTGEFTALGVYDTVIVLPRHAMPGKTI
EV-E1 VG-5-27 (D00214)	RALAVIQSVSLIAAVAGTIYIVYRLFSGMQ	GPYSGIGTNYATKKPVVRQVQTQ	GPLFDFGVSLLKKNIRTVKTGAGEFTALGVYDTVVVLPRHAMPGKTI
EV-E1 LC-R4 (DQ092769)	RALAVIQSVSLIAAVAGTIYIVYRLFSGMQ	GPYSGVGASYATKKPVVRQVQTQ	GPLFDFGVSLLKKNIRTVKTGTGEFTALGVYDTVIVLPRHAMPGKTI
EV-E1 Vir 404/03 (DQ092771)	RALSVVQSISLVVAVAGTIYIVYRLFSGIQ	GPYSGIGTNYATKKPVVRQVQTQ	GPLFDFGVSLLKKNIRTVKTSTGEFTALGVYDTVIVLPRHAMPGKTI
EV-E2 PS83 (DQ092793)	RALAVIQSVSLIAAVAGTIYIVYRLFSGMQ	GPYSGIGTNYATKKPVVRQVQTQ	GPLFDFGVSLLKKNIRTVKTDSGEFTALGVYDTAIVLPRHAMPGRTV
EV-E2 PS42 (DQ092792)	RALAVIQSVSLIAAVAGTIYIVYRLFSGMQ	GPYSGIGTNYATKKPVVRQVQTQ	GPLFDFGVSLLKKNIRTVKTDSGEFTALGVHDTVIVLPRHAMPGRTV
EV-E2 SL305 (AF123433)	RALAVIQSVSLIAAVAGTIYIVYRLFSGMQ	GPYSGIGTNYATKKPVVRQVQTQ	GPLFDFGVSLLKKNIRTVKTGAGEFTALGVYDTVIVLPRHAMPGKTI
EV-E2 IS1/Bos_taurus/JPN/1990	RALAVIQSVSLIAAVAGTIYIVYRLFSGMQ	GPYSGIGSNYATKKPVVRQVQTQ	GPLFDFGVSLLKKNIRTVKTGTGEFTALGVYDTVIVLPRHAMPGKTI
EV-E2 K2577 (AF123432)	RALAVIQSVSLIAAVAGTIYIVYRLFSGMQ	GPYSGIGSNYATKKPVVRQVQTQ	GPLFDFGVSLLKKNIRTVKTSTGEFTALGVYDTVIVLPRHAMPGKTI
EV-E3 HY12 (KF748290)	RALAVIQSVSLIAAIAGTIYIVYRLFSGMQ	GPYSGIGTNYATKKPVVRQVQTQ	GPLFDFGVSLLKKNIRTVRTSSGEFTALGVHDTIIVLPRHAMPGKTI
EV-F1 BEV-261 (NC_021220)	RALIVLQSATLIATICGVIYVVYKLFAGLQ	GPYSGIHTNYQKVKPVVRQVTTQ	GPLLDFAMSLLKKNIRTVVTKTGEFTGLGVYDTFMVLPRHAMAHGEV
EV-F1 IL/alpaca (KC748420)	RALIVLQSATLIATICGVIYVVYKLFAGLQ	GPYSGIHTNYQKVKPVVRQVTTQ	GPLLDFAMSLLKKNIRTVVTKTGEFTGLGVYDTFMVLPRHAMAHDEV
EV-F2 BHM26 (HQ917060)	RALIVLQSATLIATICGVIYVVYKLFAGLQ	GPYSGIHTNYQKVKPVVRQVTTQ	GPLVDFAVSLLKKNIKTVQTKVGEFTGLGVYDTFMVLPRHAMAHDEI
EV-F2 BJ001 (HQ663846)	RALIVLQSATLIATICGVIYVVYKLFAGLQ	GPYSGIHTNYQKVKPVVRQVTTQ	GPLVDFAVSLLKKNIKTVQTKVGEFTGLGVYDTFMVLPRHAMAHDEI
EV-F2 PS89 (DQ092795)	RALIILQSATLIATICGVIYVVYKLFAGLQ	GPYSGIHTNYQKVKPVVRQVTTQ	GPLLDFAMSLLKKNIRTVVTKTGEFTGLGVYDTFMVLPRHAMAHGEV
EV-F2 3A (AY508697)	RALIVLQSATLIATICGVIYVVYKLFAGLQ	GPYSGIHTNYQKVKPVVRQVTTQ	GPLLDFAMSLLKKNIRTVVTKTGEFTGLGVYDTFMVLPRHAMAHGEV
Ho12/Bos taurus/JPN/2014	RALIVLQSATLIATICGVIYIVYKLFAGLQ	GPYSGIHTNYQKVKPVVRQVTTQ	GPLLDFAMSLLKKNIRTVVTKTGEFTGLGVYDTFMVLPRHAMAHGEV
IS2/Bos taurus/JPN/1990	RALIVLQSATLIATICGVIYVVYKLFAGLQ	GPYSGIHTNYQKVKPVVRQVTTQ	GPLLDFAMSLLKKNIRTVVTKTGEFTGLGVYDTFMVLPRHAMAHGEV
EV-F3 PS87/Belfast (DQ092794)	RALIVLQSATLIATICGVIYVVYKLFAGLQ	GPYSGIHTNYQKVKPVVRQVTTQ	GPLLDFAMSLLKKNIRTVVTKTGEFTGLGVYDTFMVLPRHAMAYGEV
EV-F4 Possum enterovirus W1 (AY462106)	RALIILQSATLIATICGTIYVVYRLFAGLQ	GPYSGIHTNYQKVKPVVRQVTTQ	GPLLDFAVSLLNKNIRTVTTKNGDFTGLGIYDTFMVLPRHAMAHGEV
EV-F4 Possum enterovirus W6 (AY462107)	RALIVLQSATLIATICGVIYVVYKLFAGLQ	GPYSGIHSNYQKVKPVVRQVTTQ	GPLLDFAVSLLKKNIKTVTTKSGDFTGLGVYDTFMVLPRHAMAHGEI
EV-A (NC 001612)	RAVLIMQSIATVVAVVSLVYVIYKLFAGFQ	GAYSGAP-KQTLKKPILRTATVQ	GPSLDFALSLLRRNIRQVQTDQGHFTMLGVRDRLAVLPRHSQPGKTI
EV-B (NC 001472)	RAFICLQALTTFVSVAGIIYIIYKLFAGFQ	GAYTGMP-NQKPKVPTLRQAKVQ	GPVFEFAVAMMKRNSSTVKTEYGEFTMLGIYDRWAVLPRHAKPGPTI
EV-C (NC 002058)	RAMTILQAVTTFAAVAGVVYVMYKLFAGHQ	GAYTGLP-NKKPNVPTIRTAKVQ	GPGFDYAVAMAKRNIVTATTSKGEFTMLGVHDNVAILPTHASPGESI
EV-D (NC 001430)	RAFIALQAITTFVSIAGVVYVIYKLFAGIQ	GPYTGLP-NQKPKVPTLRTAKVQ	GPSLDFAQAIMRKNTVIARTSKGEFTMLGIYDRIAVVPTHASVEEEI
EV-G (NC 004441)	IATTILSSLVLLTSVITLVYLVYRLFAGYQ	GPYTGLP-NAKPKPPVLREVRAQ	GPLMDFGVGMMKKNIVTVRTGAGEFTGLGVHDHVLVLPKHSHPAEIV
EV-H (NC 003988)	KLSIILQAVTTFVTIAGVIFLVYKLMANFQ	GPYTGLF-KAKHKSTTLRTATVQ	GPSLDFSISLLRHNIRTATTVNGEFTMLGIYDRVAVLPSHAGVTDTI
EV-J (NC 010415)	RALTILQAVSTFVAVAGCIYIVYKLFAGFQ	GAYTGLP-FNKPKVPTIRQAKVQ	GPSFEFAVAMMKRNSAIIKTGQGEFTMLGIWDKWAVVPRHAKPGDTV
RV-A (NC 001617)	IANMTIGIIANVVSIVGVIYIIYKLFCTLQ	GPYSGEPK-PKSRAP-ERRVVTQ	GPEEEFGRSLLKHNCCVVTTDKGKFTGLGIYDQVMVLPTHSDPGSEI
RV-B (NC 001490)	QASMIINTILMFVSTLGIVYVIYKLFAQTQ	GPYSGNPPHNKLKAPTLRPVVVQ	GPNTEFALSLLRKNIMTITTSKGEFTGLGIHDRVCVIPTHAQPGDDV
RV-C (NC 009996)	YVTYALNCLGSLVIILGTIYALYRLMCIAQ	GPYTGMPTTPKKKPE-LRRATLQ	GPEHEFLNALIRRNCHIITTDKGEFNLLGIYSNCAVVPTHAEPGDVV

Strain	
AN12/Bos taurus/JPN/2014	EVDGKKVEIEDAYDLNDTTQTSLELTVIKLKQNEKFRDIRSLIPDQISETTEALVIVNTSAYPNLFMPVGAVKDYGYLNLAGRPTHRTLMYNFPTRAGQC
EV-E1 PA12-24791 (KC667561)	EMNGKDVEVLDAYDLNDKTDTSLELTIVRLKMNEKFRDIRAMIPDQITDYNEAVVVVNTSYYPQLFMPVGRVKDYGFLNLAGRPTHRVLMYEFPTKAGQC
EV-E1 VG-5-27 (D00214)	EMNGKDIEVLDAYDLNDKTDTSLELTIVKLKMNEKFRDIRAMVPDQITDYNEAVVVVNTSYYPQLFTCVGRVKDYGFLNLAGRPTHRVLMYEFPTKAGQC
EV-E1 LC-R4 (DQ092769)	EMNGKDIEVLDAYDLNDKTDTSLELTIVKLKMNEKFRDIRAMIPDQITDYSEAVVVVNTSYYPQLFMPVGRVKDYGFLNLAGRPTHRVLMYEFPTKAGQC
EV-E1 Vir 404/03 (DQ092771)	EMNGRDVEVLDAYDLNDKTDTSLELTIVKLKMNEKFRDIRAMIPDQITDYNEAVVVVNTSYYPQLFMPVGRVKDYGFLNLAGRPTHRVLMYEFPTKAGQC
EV-E2 PS83 (DQ092793)	VVNGKDVEVLDAYDLNDKTDTSLELTIVKLKMNEKFRDIRAMIPDQITDYNEAVVVVNTSYYPQLFMPVGRVKDYGFLNLAGRPTHRVLMYEFPTKAGQC
EV-E2 PS42 (DQ092792)	VVNGKDVEVLDAYDLNDKTDTSLELTIVKLKMNEKFRDIRAMIPDQITDYNEAVVVVNTSYYPQLFMPVGRVKDYGFLNLAGRPTHRVLMYEFPTKAGQC
EV-E2 SL305 (AF123433)	EMNGKDVEVLDAYDLNDKTDTSLELTIVKLKMNEKFRDIRAMIPDQITDYNEAIVVVNTSYYPQLFMPVGRVKDYGFLNLAGRPTHRVLMYEFPTKAGQC
EV-E2 IS1/Bos_taurus/JPN/1990	EMNGRDVEVLDAYDLNDKTDTSLELTIVKLKMNEKFRDIRAMIPDQITDYNEAVVVVNTSYYPQLFMPVGRVKDYGFLNLAGRPTHRVLMYEFPTKAGQC
EV-E2 K2577 (AF123432)	EMNGKDIEVLDAYDLNDTTDTSLELTIVKLKMNEKFRDIRAMIPDQITDYNEAIVVVNTSYYPQLFMPVGRVKDYGFLNLAGRPTHRVLMYEFPTKAGQC
EV-E3 HY12 (KF748290)	EMNGRDIEVLDAYDLNDKTDTSLELTIVRLKMNEKFRDIRAMIPDQITDYNEAIVVVNTSYYPQLFMPVGRVKDYGFLNLAGRPTHRVLMYEFPTKAGQC
EV-F1 BEV-261 (NC_021220)	EVDGKKVEVEDAYDLNDTTQTSLELTVLKLRQNEKFRDIRSLIPDQISETTEALVIVNTSAYPNLFMPVGAVKDYGYLNLAGRPTHRTLMYNFPTRAGQC
EV-F1 IL/alpaca (KC748420)	EVDGKKVQIEDAYDLNDTTQTSLELTVLKLKQNEKFRDIRSLIPDQISETTEALVIVNTSAYPNLFMPVGAVKDYGYLNLAGRPTHRTLMYNFPTRAGQC
EV-F2 BHM26 (HQ917060)	QIDGKTVKIEDAYDLNDTTQTSLELTIVKLKQNEKFRDIRAMIPENITETSEALVIVNTTAYPNLFMPVGAVKDYGYLNLAGRPTHRTLMYNFPTRAGQC
EV-F2 BJ001 (HQ663846)	QVEGKTVKIEDAYDLNDPTQTSLELTIVKLKQNEKFRDIRALIPESITETNEALVIVNTSAYPNLFMPVGAVKDYGYLNLAGRPTHRTLMYNFPTRAGQC
EV-F2 PS89 (DQ092795)	EVDGKRVEIEDAYDLNDTTQTSLELTVLKLRQNEKFRDIRSLIPDQISETTEALVIVNTSAYPNLFMPVGAVKDYGYLNLAGRPTHRTLMYNFPTRAGQC
EV-F2 3A (AY508697)	EIDGRKVGIEDAYDLNDITQTSLELTVLKLKQNEKFRDIRSLIPDQISETTEALVIVNTSAYPNLFMPVGAVKDYGYLNLAGRPTHRTLMYNFPTRAGQC
Ho12/Bos taurus/JPN/2014	EVDGKRVEIEDAYDLNDTTQTSLELTVLKLKQNEKFRDIRSLIPDQISETTEALVIVNTSAYPNLFMPVGAVKDYGYLNLAGRPTHRTLMYNFPTRAGQC
IS2/Bos taurus/JPN/1990	EVDGKRVEIEDAYDLNDTTQTSLELTVLKLKQNEKFRDIRSLIPDQISETTEALVIVNTSAYPNLFMPVGAVKDYGYLNLAGRPTHRTLMYNFPTRAGQC
EV-F3 PS87/Belfast (DQ092794)	EVDGRKVEIEDAYDLNDTTQTSLELTVLKLRQNEKFRDIRSLIPDQISETAEALVIVNTSAYPNLFMPVGAVKDYGYLNLAGRPTHRTLMYNFPTRAGQC
EV-F4 Possum enterovirus W1 (AY462106)	AINGKTIEVEDAYDLVDTTQTSLELTVVKLKQNEKFRDIRSLIPDQISETTEALVIVNTPAYPNLFMPVGAVKDYGYLNLAGRPTHRTLMYNFPTRAGQC
EV-F4 Possum enterovirus W6 (AY462107)	MIDGKTTAIEDAYDLVDTTQTSLELTVLKLKQNEKFRDIRSLIPDQISEATEALVIVNTPAYPNLFMPVGAVKDYGYLNLAGRPTHRTLMYNFPTRAGQC
EV-A (NC 001612)	WVEHKLINILDAVELVDEQGVNLELTLVTLDTNEKFRDITKFIPENISAASDATLVINTEHMPSMFVPVGDVVQYGFLNLSGKPTHRTMMYNFPTKAGQC
EV-B (NC 001472)	LMNDQEIGVLDAKELVDKDGTNLELTLLKLNRNEKFRDIRGFLAKEEVEVNEAVLAINTSKFPNMYIPVGQVTEYGFLNLGGTPTKRMLMYNFPTRAGQC
EV-C (NC 002058)	VIDGKEVEILDAKALEDQAGTNLEITIITLKRNEKFRDIRPHIPTQITETNDGVLIVNTSKYPNMYVPVGAVTEQGYLNLGGRQTARTLMYNFPTRAGQC
EV-D (NC 001430)	YINDVPVKVKDAYALRDINDVNLEITVVELDRNEKFRDIRGFLPKYEDDYNDAILSVNTSKFPNMYIPVGQTLNYGFLNLGGTPTHRILMYNFPTRAGQC
EV-G (NC 004441)	VVDGKETPVEDAYNLTDEQGVSLELTLVTLKRNEKFRDIRAMIPENPCGTNEAVVCVNTSNFPNAFLPVGKVEYYGYLNLAGSPTHRTMMYNFPTKAGQC
EV-H (NC 003988)	LVDGKTVPVVNAVNLVDPEGVNLEITVLTLGWNEKFRDIRKFIPETVEEGTEGTLIVQTSNYPHLICPIGTVKEYGYLNLSGTPTHRVLMYNFHTRIGQC
EV-J (NC 010415)	IVDDKELRVLDAKELVDAEGVNLELTMIKLDRNEKFRDIRGFIPKESGELSEAVLAINTSKFPNMFIPVGAVKEYGFLNLSGTPTHRMLMYNFPTKSGQC
RV-A (NC 001617)	LVDGVKVKVSDSYDLHNHEGVKLEITVVKLIRNEKFKDIRKYLPSREDDYPACNLALLANQDEPTIISVGDAVSYGNILLSGTNTARMIKYHYPTKAGYC
RV-B (NC 001490)	LVNGQKIRVKDKYKLVDPENINLELTVLTLDRNEKFRDIRGFISEDLEGVDAT-LVVHSNNFTNTILEVGPVTMAGLINLSSTPTNRMIRYDYATKTGQC
RV-C (NC 009996)	DIDGRLVRVLKQQVLTDMNDVDTEVTVLWLDQNEKFRDIRRFIPEHQQDWHNIHLATNVTKFPMLNVEVGHTVPYGEINLSGNATCRLYKYDYPTQPGQC

Table S2. Multiple alignments result using amino acid sequences of polyprotein (continued)

Strain		3D
AN12/Bos taurus/JPN/2014	GGVAVSMGKVIGIHIGGNGAQGFAAALLRRYFTQP	QGEIEFMEKSKDAGYPIINAPTKTKLHPSVFFDIFPGEKEPAVLHKKDKRLEVDFEEALFSKYIG
EV-E1 PA12-24791 (KC667561)	GGVVISMGKIVGIHVGGNGAQGFAASLLRRYFTAE	QGQIEYIEKSKDAGYPVINAPTQTKLEPSVFFDVFPGVKEPAVLHKKDKRLETNFEEALFSKYIG
EV-E1 VG-5-27 (D00214)	GGVVISMGKIVGVHVGGNGAQGFAASLLRRYFTAE	QGQIEYIEKSKDAGYPVINAPTQTKLEPSVFFDVFPGVKEPAVLHKKDKRLETNFEEALFSKYIG
EV-E1 LC-R4 (DQ092769)	GGVVISMGKIVGIHVGGNGAQGFAASLLRRYFTAE	QGQIEYIEKSKDAGYPVINAPTQTKLEPSVFFDVFPGVKEPAVLHKKDRRLETNFEEALFSKYIG
EV-E1 Vir 404/03 (DQ092771)	GGVVISMGKIVGIHVGGNGAQGFAASLLRRYLTAE	QGQIEYIEKSKDAGYPVINAPTQTKLEPSVFFDVFPGVKEPAVLHKKDKRLETNFEEALFSKYIG
EV-E2 PS83 (DQ092793)	GGVVISMGKIVGIHVGGNGAQGFAASLLRRYFTAE	QGQIEYIEKSKDAGFPIINAPTQTKLEPSVFYDVFPGVKEPAVLHKKDKRLEVNFEEALFSKYIG
EV-E2 PS42 (DQ092792)	GGVVISMGKIVGIHVGGNGAQGFAASLLRRYFTAE	QGQIEYIEKSKDAGFPIINAPTQTKLEPSVFYDVFPGVKEPAVLHKKDKRLEVNFEEALFSKYIG
EV-E2 SL305 (AF123433)	GGVVVSMGKIVGIHVGGNGAQGFAASLLRRYFTAE	QGQIEYIEKSKDAGYPVINAPTQTKLEPSVFFDVFPGVKEPAVLHKKDKRLETNFEEALFSKYIG
EV-E2 IS1/Bos_taurus/JPN/1990	GGVVISMGKIVGIHVGGNGAQGFAASLLRRYFTAE	QGQIEYIEKSKDAGYPVINAPTQTKLEPSVFFDVFPGVKEPAVLHKKDKRLETNFEEALFSKYIG
EV-E2 K2577 (AF123432)	GGVVVSMGKIVGIHVGGNGAQGFAASLLRRYFTAE	QGQIEYIEKSKDAGYPVINAPTQTKLEPSVFFDVFPGVKEPAVLHKKDKRLETNFEEALFSKYIG
EV-E3 HY12 (KF748290)	GGVVVSMGKIVGIHVGGNGAQGFAASLLRRYFTVE	QGQIEYIEKSKDAGYPVINAPTQTKLEPSVFFDVFPGVKEPAVLHKKDKRLETNFEEALFSKYIG
EV-F1 BEV-261 (NC_021220)	GGVAVSMGKVIGIHIGGNGAQGFAAALLRRYFTQP	QGEIEFMEKSKDAGYPIINAPTKTKLHPSVFFDVFPGEKEPAVLHRKDKRLEVDFEEALFSKYIG
EV-F1 IL/alpaca (KC748420)	GGVAVSMGKVIGIHIGGNGAQGFAAALLRRYFTQP	QGEIEFMEKSKDAGYPIINAPTKTKLHPSVFFDVFPGEKEPAVLHKKDKRLEVDFEEALFSKYIG
EV-F2 BHM26 (HQ917060)	GGVAISMGKIIGIHIGGNGAQGFSAALLRRYFTQP	QGEIEFMEKSKDAGYPIINAPTKTKLHPSVFYDVFPGEKEPAVLHKKDPRLEVDFEEALFSKYIG
EV-F2 BJ001 (HQ663846)	GGVAISMGKVIGIHIGGNGAQGFSAALLRRYFTQP	QGEIEFMEKSKDAGYPTINAPTKTKLHPSVFHDVFPGEKEPAVLHKKDPRLEVDFEEALFSKYIG
EV-F2 PS89 (DQ092795)	GGVAVSMGKVIGIHIGGNGAQGFAAALLRRYFTQP	QGEIEFMEKSKDAGYPIINAPTKTKLHPSVFFDVFPGEKEPAVLHKKDKRLEVDFEEALFSKYIG
EV-F2 3A (AY508697)	GGVAVSMGKVIGIHIGGNGAQGFAAALLRRYFTQP	QGEIEFMEKSKDAGYPIINAPTKTKLHPSVFFDVFPGEKEPAVLHKKDKRLEVDFEEALFSKYIG
Ho12/Bos taurus/JPN/2014	GGVAVSMGKVIGIHIGGNGAQGFAAALLRRYFTQP	QGEIEFMEKSKDAGYPIINAPTKTKLHPSVFFDVFPGEKEPAVLHKKDKRLEVDFEEALFSKYIG
IS2/Bos taurus/JPN/1990	GGVAVSMGKVIGIHIGGNGAQGFAAALLRRYFTQP	QGEIEFMEKSKDAGYPIINAPTKTKLHPSVFFDVFPGEKEPAVLHKRDKRLEVDFEEALFSKYIG
EV-F3 PS87/Belfast (DQ092794)	GGVAVSMGKVIGIHIGGNGAQGFAAALLRRYFTQP	QGEIEFMEKSKDAGYPIINAPTKTKLHPSVFFDVFPGEKEPAVLHKKDKRLEVDFEEALFSKYIG
EV-F4 Possum enterovirus W1 (AY462106)	GGVAISMGKVIGIHIGGNGAQGFAAALLRRYFTQP	QGKIELVEKSKDAGYPIINAPTKTKLYPSVFFDVFPGEKEPAVLHKKDKRLEVDFEEALFSKYIG
EV-F4 Possum enterovirus W6 (AY462107)	GGVAVSMGKVIGIHIGGNGAQGFSAALLRRYFTQP	QGEIELVEKSKDAGYPIINAPTKTKLQPSVFFDVFPGEKEPAVLHKKDKRLEVDFEEAIFSKYIG
EV-A (NC 001612)	GGVVTSVGKVIGIHIGGNGRQGFCAGLKRSYFASE	QGEIQWVKPNKETGRLNINGPTRTKLEPSVFHDVFEGNKEPAVLHSRDPRLEVDFEQALFSKYVG
EV-B (NC 001472)	GGVLMSTGKVLGIHVGGNGHQGFSAALLKHYFNDE	QGEIEFIESSKEAGFPVINTPSKTKLEPSVFHQVFEGNKEPAVLRNGDPRLRANFEEAIFSKYIG
EV-C (NC 002058)	GGVITCTGKVIGMHVGGNGSHGFAAALKRSYFTQS	QGEIQWMRPSKEVGYPIINAPSKTKLEPSAFHYVFEGVKEPAVLTKNDPRLKTDFEEAIFSKYVG
EV-D (NC 001430)	GGVVTTTGKVIGIHVGGNGAQGFAAMLLQNYFTEK	QGEIVSIEKTG----VFINAPAKTKLEPSVFHEVFEGVKEPAVLHSKDKRLKVDFEEAIFSKYVG
EV-G (NC 004441)	GGVVLSTGKVLGIHIGGNGAQGFCAALKRSYFTKP	QGKIDWVEPSKKHGFPVINAPSKTKLEPSVFFDVFEGVKEPAALHPKDPRLEVNLEEALFSKYTG
EV-H (NC 003988)	GGVIATTGRVLGIHVGGNGAQGFAASLFRKYFAIT	QGSIEFKRRLSWQGLRVLY-PEKTSLEPSVFHDIFPGVKQPAVLSKKDPRCQVNFEDAIFSKYKG
EV-J (NC 010415)	GGVLMATGKVIGIHVGGNGHQGFAATLLRDYFNEE	QGEIEWMETNKESGYPIINAPTKTKLEPSVFHDIFPGVKEPAVLNKNDPRLKVDFEEAIFSKYVG
RV-A (NC 001617)	GGVLYKVGSILGIHVGGNGRDGFSAMLLKSYFGET	QGLITKELPVSVKNLPSVHVSSKTRLQPSVFHDVFPGTKEPAVLSSNDPRLETDFDSALFSKYKG
RV-B (NC 001490)	GGVLCATGKIFGIHVGGNGRQGFSAQLKKQYFVEK	QGQVIARHKVREFNINPVNTATKSKLHPSVFYDVFPGDKEPAVLSDNDPRLEVKLTESLFSKYKG
RV-C (NC 009996)	GAVLANTGNIIGIHVGGNGRVGYAAALLRKYFAEE	QGAIISKCNVKEKNWKPVNTPSQTKLHPSVFHDVFPGVKEPAALHPKDPRLEVDLTTAVMSKYKG

Strain	Sequence
AN12/Bos taurus/JPN/2014	NIHKPVTEEMEIAIDHYANQLKQLDIDPTPISMEDAIYGTEGLEALDLGTSAGYPYVALGIKKRDILNKETRDVTKMQQCIDKYGLNLPMVTYVKDELRS
EV-E1 PA12-24791 (KC667561)	NVQRDMPEELLIAIDHYSEQLKMLNIDPRPISMEDAIYGTEGLEALDLGTSAGYPYVAMGIKKRDILNKETRDVTKMQECINKYGLNLPMVTYVKDELRA
EV-E1 VG-5-27 (D00214)	NVQRDMPEELLIAIDHYSEQLKMLNIDPRPISMEDAIYGTEGLEALDLGTSASYPYVAMGIKKRDILNKETRDVTKMQECIDKYGLNLPMVTYVKDELRA
EV-E1 LC-R4 (DQ092769)	NVQRDMPEELLIAIDHYSEQLKMLNIDPRPISMEDAIYGTEGLEALDLGTSAGYPYVAMGIKKRDILNKETRDVTKMQECINKYGLNLPMVTYVKDELRA
EV-E1 Vir 404/03 (DQ092771)	NVQRDMPEELLIAIDHYAEQLKMLNIDPRPISMEDAIYGTEGLEALDLGTSAGYPYVAMGIKKRDILNKETRDVTKMQECIDKYGLNLPMVTYVKDELRA
EV-E2 PS83 (DQ092793)	NVQRDTPDELLIAIDHYSEQLKMLNIDPRPISMEDAIYGTEGLEALDLGTSAGYPYVAMGIKKRDILNKETRDVTKMQECIDKYGLNLPMVTYVKDELRA
EV-E2 PS42 (DQ092792)	NVQRDMPDELLIAIDHYSEQLKMLNIDPRPISMEDAIYGTEGLEALDLGTSAGYPYVAMGIKKRDILNKETRDVTKMQECIDKYGLNLPMVTYVKDELRA
EV-E2 SL305 (AF123433)	NVQRDMPEELLIAIDHYSEQLKMLNIDPRPISMEDAIYGTEGLEALDLGTSAGYPYVAMGIKKRDILNKETRDVTQMQECINKYGLNLPMVTYVKDELRA
EV-E2 IS1/Bos_taurus/JPN/1990	NVQRDMPEELLIAIDHYSEQLKMLNIDPRPISMEDAIYGTEGLEALDLGTSAGYPYVAMGIKKRDILNKETRDVTKMQECIDKYGLNLPMVTYVKDELRA
EV-E2 K2577 (AF123432)	NVQRDMPEELLIAIDHYSEQLKMLNIDPRPISMEDAIYGTEGLEALDLGTSAGYPYVAMGIKKRDILNKETRDVTKMQECINKYGLNLPMVTYVKDELRA
EV-E3 HY12 (KF748290)	NVQRDMPEELLVAIDHYSEQLKMLNIDPRPISMEDAIYGTEGLEALDLGTSAGYPYVAMGIKKRDILNKETRDTTKMQECIDKYGLNLPMVTYVKDELRA
EV-F1 BEV-261 (NC_021220)	NIHKPVTEEMEIAIDHYANQLKQLNIDPTPISMEDAIYGTEGLEALDLGTSAGYPYVALGIKKRDILNKETRDVSKMQQCIDKYGLNLPMVTYVKDELRS
EV-F1 IL/alpaca (KC748420)	NIHKPVTEEMEIAIDHYANQLKQLNIDPTPISMEDAIYGTEGLEALDLGTSAGYPYVALGIKKRDILNKETRDVSKMQQCIDKYGLNLPMVTYVKDELRS
EV-F2 BHM26 (HQ917060)	NKHIAVTEEMEIAIDHYANQLKQLDIDPNPISMEDAIYGTEGLEALDLGTSAGYPYVALGIKKRDILNKETRDVSKMQQCIDKYGLNLPMVTYVKDELRS
EV-F2 BJ001 (HQ663846)	NKHIAVTEEMEIAIDHYANQLKQLDIDPSPISMEDAIYGTEGLEALDLGTSAGYPYVALGIKKRDILNKETRDVSKMQQCIDKYGLNLPMVTYVKDELRS
EV-F2 PS89 (DQ092795)	NIHKPVTEEMEIAIDHYANQLKQLNIDPTPISMEDAIYGTEGLEALDLGTSAGYPYVALGIKKRDILNKETRDVSKMQQCIDKYGLNLPMVTYVKDELRS
EV-F2 3A (AY508697)	NIHKPVTEEMEIAIDHYANQLKQLDIDPTPISMEDAIYGTEGLEALDLGTSAGYPYVALGIKKRDILNKETRDTSKMQQCIDKYGLNLPMVTYVKDELRS
Ho12/Bos taurus/JPN/2014	NVHKPVTEEMEIAIDHYANQLKQLNIDPTPISMEDAIYGTEGLEALDLGTSAGYPYVALGIKKRDILNKETRDVTKMQRCIDKYGLNLPMVTYVKDELRS
IS2/Bos taurus/JPN/1990	NIHKPVTEEMEIAIDHYANQLKQLNIDPTPISMEDAIYGTEGLEALDLGTSAGYPYVALGIKKRDILNKETRDVTKMQQCIDKYGLNLPMVTYVKDELRS
EV-F3 PS87/Belfast (DQ092794)	NIHKPVTEEMEIAIDHYANQLKQLNIDPTPISMEDVIYGTEGLEALDLGTSAGYPYVALGIKKRDILNKETRDVSKMQQCIDKYGLNLPMVTYVKDELRS
EV-F4 Possum enterovirus W1 (AY462106)	NIDKPVTEEMEIAIDHYANQLKQLNIDPTPISMEEAIYGTEGLEALDLGTSAGYPYVALGIKKRDILNKETRDTSKMQQCIEKYGLNLPMVTYVKDELRS
EV-F4 Possum enterovirus W6 (AY462107)	NIDRPVTEEMEIAIDHYANQLKQLNIDPTPISMEEAIYGTEGLEALDLGTSAGYPYVALGIKKRDILNKETRDISKMQQCIDKYGLNLPMVTYVKDELRS
EV-A (NC 001612)	NTLHEPDEYIKEAALHYANQLKQLDINTSQMSMEEACYGTENLEAIDLHTSAGYPYSALGIKKRDILDPTTRDVSKMKFYMDKYGLDLPYSTYVKDELRS
EV-B (NC 001472)	NVNTHVDEYMLEAVDHYAGQLATLDISTEPMRLEDAVYGTEGLEALDLTTSAGYPYVALGIKKRDILSKKTRDLTKLKECMDKYGLNLPMVTYVKDELRS
EV-C (NC 002058)	NKITEVDEYMKEAVDHYAGQLMSLDINTEQMCLEDAMYGTDGLEALDLSTSAGYPYVAMGKKKRDILNKQTRDTKEMQKLLDTYGINLPLVTYVKDELRS
EV-D (NC 001430)	NKTMLMDEYMEEAVDHYVGCLEPLDISTEPIKLEEAMYGMDGLEALDLTTSAGYPYLLQGKKKRDIFNRQTRDTTEMTKMLDKYGVDLPFVTFVKDELRS
EV-G (NC 004441)	NVDIEMPEEMKEAVDHYANQLLALDIPTEPLSMEEAIYGTEGLEALDLTTSAGYPYVTMGIKKRDILNKETRDVKKMQECIDKYGLNLPMVTYIKDELRS
EV-H (NC 003988)	NVNKEIDQYMAEAIDDYASNLLSLGINTEPNSMEQAVYGMDGLEALDLSTSAGYPYVTQGIKKRDLF-KPNRDLSKFKECMDKYGLNLPMVTFLKDELRP
EV-J (NC 010415)	NVNTHVDEYMQEAIDHYAGQLMTLDIDISEMCLEDAVYGTEGLEALDLTTSAGYPYVTMGIKKRDILSKKTRDLSKLKECMDKYGLNLPMVTYVKDELRS
RV-A (NC 001617)	NPACQVTPHMKIAVAHYAAQLSTLDINPQPLSLEESVFGIEGLEALDLNTSAGFPYVSLGIKKKDLIDKKTKDITKLRKAIDEYGIDLPMVTFLKDELRK
RV-B (NC 001490)	NVNTEPTENMLVAVDHYAGQLLSLDIPTSELTLKEALYGVDGLEPIDITTSAGFPYVSLGIKKRDILNKETQDTEKMKFYLDKYGIDLPLVTYIKDELRS
RV-C (NC 009996)	NIDVPMNEYIETAVDHYAAQLYMLDINPEPITMEQAIYGYANLEPLDLTTSPGFPYVSLGVKKKDILNNATKDTRKMQQMLDLYGIDLPYITFLKDELRA

Table S2. Multiple alignments result using amino acid sequences of polyprotein (continued)

AN12/Bos taurus/JPN/2014	KEKVKKGKSRLIEASSLNDSVAMRCAFGNLYKAFHTNPGTLTGCAVGCNPETFWSKIPVMMDGE-LFGFDYTAYDASLSPVWFQCLYLLLEKIGFGHCKH
EV-E1 PA12-24791 (KC667561)	PDKIKKGKSRLIEASSLNDSVAMRCYFGNLYKAFHTNPGTISGCAVGCDPEIFWSKIPVMMDGE-LFGFDYTAYDASLSPMWFHALAEVLRRIGFVECKH
EV-E1 VG-5-27 (D00214)	PDKIRKGKSRLIEASSLNDSVAMRCYFGNLYKVFHTNPGTISGCAVGCDPETFWSKIPVMMDGE-LFGFDYTAYDASLSPMWFHALAEVLRRIGFVECKH
EV-E1 LC-R4 (DQ092769)	PDKIKKGKSRLIEASSLNDSVAMRCYFGNLYKAFHTNPGTISGCAVGCDPETFWSKIPVMMDGE-LFGFDYTAYDASLSPMWFHALAEVLRRIGFVECKH
EV-E1 Vir 404/03 (DQ092771)	PDKIKKGKSRLIEASSLNDSVAMRCYFGNLYKAFHTNPGTISGCAVGCDPETFWSKIPVMMDGE-LFGFDYTAYDASLSPMWFHALAEVLRRIGFVECKH
EV-E2 PS83 (DQ092793)	PDKIKKGKSRLIEASSLNDSVAMRCYFGNLYKAFHTNPGTITGCAVGCDPETFWSKIPVMMDGE-LFGFDYTAYDASLSPMWFHALAEVLRRIGFVECKH
EV-E2 PS42 (DQ092792)	PDKIKKGKSRLIEASSLNDSVAMRCYFGNLYKAFHTNPGTITGCAVGCDPETFWSKIPVMMDGE-LFGFDYTAYDASLSPMWFHALAEVLRRIGFVECKH
EV-E2 SL305 (AF123433)	PDKIKKGKSRLIEASSLNDSVAMRCYFGNLYKAFHTNPGTISGCAVGCDPETFWSKIPVMMDGE-LFGFDYTAYDASLSPMWFHALAEVLRRIGFVECKH
EV-E2 IS1/Bos_taurus/JPN/1990	PDKIKKGKSRLIEASSLNDSVAMRCYFGNLYKAFHTNPGTISGCAVGCDPETFWSKIPVMMDGE-LFGFDYTAYDASLSPMWFHALAEVLRRIGFVECKH
EV-E2 K2577 (AF123432)	PDKIKKGKSRLIEASSLNDSVAMRCYFGNLYKAFHTNPGTISGCAVGCDPETFWSKIPVMMDGE-LFGFDYTAYDASLSPMWFHALAEVLRRIGFVECKH
EV-E3 HY12 (KF748290)	PDKIKKGKSRLIEASSLNDSVAMRCYFGNLYKAFHTNPGTISGCAVGCDPETFWSKIPVMMDGE-LFGFDYTAYDASLSPMWFHALAEVLRRIGFVECKH
EV-F1 BEV-261 (NC_021220)	KEKVKKGKSRLIEASSLNDSVAMRCAFGNLYKAFHTNPGTLTGCAVGCNPETFWSKIPVMMDGE-LFGFDYTAYDASLSPVWFQCLYLLLEKIGFGHCKH
EV-F1 IL/alpaca (KC748420)	KEKVKKGKSRLTKR-EFNDSVAMRCAFGNLYKAFHMNPGTLTGCAVGCNPETFWSKIPVMMDGE-LFGFDYTAYDASLSPVWFQCLYLLLDKIGFGHCKH
EV-F2 BHM26 (HQ917060)	KEKVKKGKSRLIEASSLNDSVAMRCAFGNLYKAFHTNPGTLTGCAVGCNPETFWSKIPVMMDGE-LFGFDYTAYDASLSPVWFQCLYLLLEKIGFGHCKH
EV-F2 BJ001 (HQ663846)	KEKVKKGKSRLIEASSLNDSVAMRCAFGNLYKAFHTNPGTLTGCAVGCNPETFWSKIPVMMDGE-LFGFDYTAYDAGLSPVWFQCLYLLLEKIGFGHCKH
EV-F2 PS89 (DQ092795)	KEKVKKGKSRLIEASSLNDSVAMRCAFGNLYKAFHTNPGTLTGCAVGCNPETFWSKIPVMMDGE-LFGFDYTAYDASLSPVWFQCLYLLLEKIGFGHCKH
EV-F2 3A (AY508697)	KEKVKKGKSRLIEASSLNDSVAMRCAFGNLYKAFHTNPGTLTGCAVGCNPETFWSKIPVMMDGE-LFGFDYTAYDASLSPVWFQCLYLLLEKIGFGHCKH
Ho12/Bos taurus/JPN/2014	REKVKKGKSRLIEASSLNDSVAMRCAFGNLYKAFHTNPGTLTGCAVGCNPETFWSKIPVMMDGE-LFGFDYTAYDASLSPVWFQCLYLLLEKIGFGHCKH
IS2/Bos taurus/JPN/1990	KEKVKKGKSRLIEASSLNDSVAMRCAFGNLYKAFHTNPGTLTGCAVGCNPETFWSKIPVMMDGE-LFGFDYTAYDASLSPVWFQCLYLLLEKIGFGHCKH
EV-F3 PS87/Belfast (DQ092794)	KEKVKKGKSRLIEASSLNDSVAMRCAFGNLYKAFHTNPGTLTGCAVGCNPETFWSKIPVMMDGE-LFGFDYTAYDASLSPVWFQCLYLLLEKIGFGHCKH
EV-F4 Possum enterovirus W1 (AY462106)	KEKVKKGKSRLIEASSLNDSVAMRCAFGNLYRAFHTNPGTLTGCAVGCNPETFWSKIPVMMDGE-LFGFDYTAYDASLSPVWFKCLNLLLEKIGFGHCKH
EV-F4 Possum enterovirus W6 (AY462107)	KEKVKKGKSRLIEASSLNDSVAMRCAFGNLYKAFHTNPGTLTGCAVGCNPETFWSKIPVMMDGE-LFGFDYTAYDASLSPVWFKCLNLLLEKIGFGHCKH
EV-A (NC 001612)	IDKIKKGKSRLIEASSLNDSVYLRMAFGHLYETFHANPGTITGSAVGCNPDTFWSKLPILLPGS-LFAFDYSGYDASLSPVWFRALELVLREVGYSEEAV
EV-B (NC 001472)	AEKVAKGKSRLIEASSLNDSVAMRQTFGNLYKTFHLNPGIVTGSAVGCDPDLFWSKIPVMLDGH-LVAFDYSGYDASLSPVWFACLKLLLEKLGYSHKET
EV-C (NC 002058)	KTKVEQGKSRLIEASSLNDSVAMRMAFGNLYAAFHKNPGVITGSAVGCDPDLFWSKIPVLMEEK-LFAFDYTGYDASLSPAWFEALKMVLEKIGFGDRVD
EV-D (NC 001430)	REKVEKGKSRLIEASSLNDSVAMRVAFGNLYATFHKNPGVATGSAVGCDPDLFWSKIPVXLDGK-IFAFDYTGYDASLSPVWFACLKKTLVKLGYTHQTA
EV-G (NC 004441)	KEKVKKGKSRLIEASSLNDSVAMRCYFGNLYKAFHQNPGTLTGCAVGCDPDTFWSKIPVMMDGE-LFGFDYTAYDASLSPLMFQALQMVLEKIGFGEGKH
EV-H (NC 003988)	LSKVEAGKTRLIEASSVNDSVAMRQAFGNLYAAFHSNPGVITGSAVGCDPDQFWSKIPVLLEGE-LFAFDYSNYDASLSPCWFAALKVLLEKIGFGDRTY
EV-J (NC 010415)	REKVEQGKSRLIEASSLNDSVAMRQCFGNLYKTFHRNPGTVTGSAVGCNPDTFWSKIPVMLDGE-LFAFDYTGYDASLSPVWFACLSRVLEKLGYDYKAG
RV-A (NC 001617)	KEKIKDGKTRVIEANSVNDTVLFRSVFGNLFSAFHKNPGIVTGSAVGCDPEVFWSTIPLMLDGECLMAFDYSNYDGSLHPVWFKCLSMLLEDIGFS---S
RV-B (NC 001490)	VDKVRLGKSRLIEASSLNDSVNMRMKLGNLYKAFHQNPGVLTGSAVGCDPDVFWSVIPCLMDGH-LMAFDYSNFDASLSPVWFVCLEKVLTKLGFAG--S
RV-C (NC 009996)	PEKIKAGKTRIVEAASVNDTVYFRTTFGNLYSTFHANPGILTGSAVGCNPDVFWSQMHAMLDGE-LIAFDYTNYDGSLEPVWFKALGKVLDQLGFP---G

AN12/Bos taurus/JPN/2014	-FIDQLCCSNHLFMDKRYVVVGGMPSGCSGTSIFNSMINNIIIRTLVLTVYKNIDLDDLKIIAYGDDVIASYPFELDAKLLADAGKSFGLIMTPPDKSSE
EV-E1 PA12-24791 (KC667561)	-FIDQLCCSHHLYMDKHYYVVGGMPSGCSGTSIFNSMINNLIIRTLVLTVYKNIDLDDLKIIAYGDDVLASYPYEIDASLLAEAGKSFGLIMTPPDKSAE
EV-E1 VG-5-27 (D00214)	-FIDQLCCSHHLYMDKHYYVVGGMPSGCSGTSIFNSMINNLIIRTLVLTVYKNIDLDDLKIIAYGDDVLASYPYEIDASLLAEAGKSFGLIMTPPDKSAE
EV-E1 LC-R4 (DQ092769)	-FIDQLCCSHHLYMDKHYYVVGGMPSGCSGTSIFNSMINNLIIRTLVLTVYKNIDLDDLKIIAYGDDVLASYPYEIDASLLAEAGKSFGLIMTPPDKSAE
EV-E1 Vir 404/03 (DQ092771)	-FIDQLCCSHHLYMDKHYYVVGGMPSGCSGTSIFNSMINNLIIRTLVLTVYKNIDLDDLKIIAYGDDVLASYPYEIDAALLAEAGKSFGLIMTPPDKSAE
EV-E2 PS83 (DQ092793)	-FIDQLCCSHHLYMDKHYYVVGGMPSGCSGTSIFNSMINNLIIRTLVLTVYKNIDLDDLKIIAYGDDVLASYPYEIDASLLAEAGKSFGLIMTPPDKSAE
EV-E2 PS42 (DQ092792)	-FIDQLCCSHHLYMDKHYYVLGGMPSGCSGTSIFNSMINNLIIRTLVLTVYKNIDLDDLKIIAYGDDVLASYPYEIDTSLLAEAGKSFGLIMTPPDKSAE
EV-E2 SL305 (AF123433)	-FIDQLCCSHHLYMDKHYYVVGGMPSGCSGTSIFNSMINNLIIRTLVLTVYKHIDLDDLKIIAYGDDVLASYPYEIDASLLAEAGKSFGLIMTPPDKSAE
EV-E2 IS1/Bos_taurus/JPN/1990	-FIDQLCCSHHLYMDKHYYVVGGMPSGCSGTSIFNSMINNLIIRTLVLTVYKNIDLDDLKIIAYGDDVLASYPYEIDASLLAEAGKSFGLIMTPPDKSAE
EV-E2 K2577 (AF123432)	-FIDQLCCSHHLYMDKHYYVVGGMPSGCSGTSIFNSMINNLIIRTLVLTVYKNIDLDDLKIIAYGDDVLASYPYEIDASLLAEAGKSFGLIMTPPDKSAE
EV-E3 HY12 (KF748290)	-FIDQLCCSHHLYMDKHYYVVGGMPSGCSGTSIFNSMINNLIIRTLVLTVYKNIDLDDLKIIAYGDDVLASYPYEIDAGLLAEAGKSFGLIMTPPDKSAE
EV-F1 BEV-261 (NC_021220)	-FIDQLCCSNHLFMDKRYVVVGGMPSGCSGTSIFNSMINNIIIRTLVLTVYKNIDLDDLKIIAYGDDVIASYPFELDAKLLADAGKSFGLIMTPPDKSAE
EV-F1 IL/alpaca (KC748420)	-FIDQLCCSNHLFMDKRYVVVGGMPSGCSGTSIFNSMINNIIIRTLVLTVYKNIDLDDLKIIAYGDDVIASYPFELDAKLLADAGKSFGLIMTPPDKSAE
EV-F2 BHM26 (HQ917060)	-FIDQLCCSNHLFMDKRYVVVGGMPSGCSGTSIFNSMINNIIIRTLVLTVYKNIDLDDLKIIAYGDDVIASYPFELDAKLLADAGKSFGLIMTPPDKSAE
EV-F2 BJ001 (HQ663846)	-FIDQLCCSNHLFMDKRYVVVGGMPSGCSGTSIFNSMINNIIIRTLVLTVYKNIDLDDLKIIAYGDDVIASYPFELDAKLLADAGKSFGLIMTPPDKSSE
EV-F2 PS89 (DQ092795)	-FIDQLCCSNHLFMDKRYVVVGGMPSGCSGTSIFNSMINNIIIRTLALTVYKNIDLDDLKIIAYGDDVIASYPFELDAKLLADAGKSFGLIMTPPDKGAE
EV-F2 3A (AY508697)	-FIDQLCCSNHLFMDKRYVVVGGMPSGCSGTSIFNSMINNIIIRTLVLTVYKNIDLDDLKIIAYGDDVIASYPFELDAKLLADAGRSFGLIMTPPDKSAE
Ho12/Bos taurus/JPN/2014	-FIDQLCCSNHLFMDKRYVVIGGMPSGCSGTSIFNSMINNIIIRTLVLTVYKNIDLDDLKIIAYGDDVIASYPFELDAKLLADAGKSFGLIMTPPDKSSE
IS2/Bos taurus/JPN/1990	-FIDQLCCSNHLFMDKRYVVVGGMPSGCSGTSIFNSMINNIIIRTLVLTVYKNIDLDDLKIIAYGDDVIASYPFELDAKLLADAGKSFGLIMTPPDKSAE
EV-F3 PS87/Belfast (DQ092794)	-FIDQLCCSNHLFMDKRYVVVGGMPSGCSGTSIFNSMINNIIIRTLVLTVYKNIDLDDLKIIAYGDDVIASYPFELDAKLLADAGKSFGLIMTPPDKSDE
EV-F4 Possum enterovirus W1 (AY462106)	-FIDQLCYSNHLYMDKRYVVAGGMPSGCSGTSIFNSMINNLIIRTLVLTVYKNIDLDDLKIIAYGDDVIASYPFELDAKLLADAGKSFGLIMTPPDKSSE
EV-F4 Possum enterovirus W6 (AY462107)	-FIDQLCYSNHLYMDKRYVVAGGMPSGCSGTSIFNSMINNIIIRTLVLTVYKHIDLDDLKIIAYGDDVIASYPFEIDAKLLADAGKSFGLIMTPPDKSSD
EV-A (NC 001612)	SLIEGINHTHHVYRNKTYCVLGGMPSGCSGTSIFNSMINNIIIRTLLIKTFKGIDLDELNMVAYGDDVLASYPFPIDCLELARTGKEYGLTMTPADKSPC
EV-B (NC 001472)	NYIDYLCNSHHLYRDKHYFVRGGMPSGCSGTSIFNSMINNIIIRTLMLKVYKGIDLDQFRMIAYGDDVIASYPWPIDASLLAEAGRDCGLIMTPADKGDC
EV-C (NC 002058)	-YIDYLNHSHHLYKNKTYCVKGGMPSGCSGTSIFNSMINNLIIRTLLLKTYKGIDLDHLKMIAYGDDVIASYPHEVDASLLAQSGKDYGLTMTPADKSAT
EV-D (NC 001430)	-FVDYLCHSVHLYKDRKYIVNGGMPSGSSGTSIFNTMINNIIIRTLLLKVYKGIDLDQFKMIAYGDDVIASYPHEIDPGLLAKAGKEYGLIMTPADKSSG
EV-G (NC 004441)	-FIDNLCYSHHLFRDKYYFVKGGMPSGCSGTSIFNSMINNIIIRTVVLQTYKGIELDQLKIIAYGDDVIASYPYRIDPAELAKAGAKLGLHMTPPDKSET
EV-H (NC 003988)	-FINYLCFSHHIFKDSHYYVSGGMPSGCSGTSIFNTMINNLIIRTLLVKVYKRISLSDLRMIAYGDDVIASLPYKIDAGKLAIAGRDYGLTMTPADKGET
EV-J (NC 010415)	RYISYLCHSYHLYKNKHYFVRGGMPSGCSGTSIFNSMINNIIIRTLLLKTYKNIDLDKFKMIAYGDDVIASYPYPIDAGLLAKAGKEYGLTMTPADKSKE
RV-A (NC 001617)	QLINQICNSKHIYKSKYYEVEGGMPSGCAGTSIFNTIINNIIIRTLVLDAYKNIDLDKLKILAYGDDVIFSYNFKLDMAVLAKEGEKYGLTITPADKSDV
RV-B (NC 001490)	SLIQSICNTHHIFRDEIYVVEGGMPSGCSGTSIFNSMINNIIIRTLILDAYKGIDLDKLKILAYGDDLIVSYPYELDPQVLATLGKNYGLTITPPDKSET
RV-C (NC 009996)	HLTQRLCNTTHIFRDTTYDVKGGMPSGISGTSIFNTMMNNIIIRTLVLETYKNINLDKLRIIAYGDDVVASYPDELDPKEIAMTAKRYGLTITPPDKTDQ

Table S2. Multiple alignments result using amino acid sequences of polyprotein (continued)

AN12/Bos taurus/JPN/2014	FVKLTWDNVTFLKRSFVKDERFPFLIHPVMKMSDIHESIRWTKDAKSTQDHVRSLCLLAWHCGQEQYEEFLEKIRSVPVGRALSLPSFKALQRSWYDSF
EV-E1 PA12-24791 (KC667561)	FVKLTWDNVTFLKRKFVRDARYPFLVHPVMDMSNIHESIRWTKDPRHTEDHVRSLCLLAWHCGEKEYNEFVTKIRSVPVGRALHLPSFKALERKWYDSF
EV-E1 VG-5-27 (D00214)	FVKLTWDNVTFLKRKFVRDARYPFLVHPVMDMSNIHESIRWTKDPRHTEDHVRSLCLLAWHCGEEEYNEFVTKIRSVPVGRALHLPSFKALERKWYDSF
EV-E1 LC-R4 (DQ092769)	FVKLTWDNVTFLKRKFVRDTRYPFLVHPVMDMSNIHESIRWTKDPRHTEDHVRSLCLLAWHCGEEEYNEFVSKIRSVPVGRALHLPSFKALERKWYDSF
EV-E1 Vir 404/03 (DQ092771)	FVKLTWDNVTFLKRRFVRDTRYPFLVHPVMDMSNIHESIRWTKDPRHTEDHVRSLCLLAWHCGEKEYNEFVSKIRTVPVGRALHLPSFKALERKWYDSF
EV-E2 PS83 (DQ092793)	FVKLTWDNVTFMKRRFVRDARHPFLVHPVMDMSNIHESIRWTKDPRHTEDHVRSLCLLAWHCGEKEYNEFVSKIRSVPVGRALHLPSFKALERKWYDSF
EV-E2 PS42 (DQ092792)	FVKLTWDNVTFLKRRFVRDARHPFLVHPVMDMSNIHESIRWTKDPRHTEDHVRSLCLLAWHCGEKEYNEFVSKIRSVPVGRALHLPSFKALERKWYDSF
EV-E2 SL305 (AF123433)	FVKLTWDNVTFLKRRFVRDARYPFLVHPVMDMSNIHESIRWTKDPRHTEDHVRSLCLLAWHCGEREYNEFITKIRSVPVGRALHLPSFKALERKWYDSF
EV-E2 IS1/Bos_taurus/JPN/1990	FVKLTWDNVTFLKRRFVRDARYPFLVHPVMDMSNIHESIRWTKDPRHTEDHVRSLCLLAWHCGEKEYNEFVSKIRSVPVGRALHLPSFKALERKWYDSF
EV-E2 K2577 (AF123432)	FVKLTWDNVTFLKRKFVRDTRYPFLVHPVMDMSNIHESIRWTKDPRHTEDHVRSLCLLAWHCGEKEYNEFVSKIRSVPVGRALHLPSFKALERKWYDSF
EV-E3 HY12 (KF748290)	FVKLTWDNVTFLKRRFVRDQRYPFLVHPVMDMSNIHESIRWTKDPRHTEDHVRSLCLLAWHCGEKEYNEFVSKIRSVPVGRALHLPSFKALERKWYDSF
EV-F1 BEV-261 (NC_021220)	FVKLTWDNVTFLKRSFVKDERFPFLIHPVMKMSDIHESIRWTKDAKSTQDHVRSLCLLAWHCGQEQYEEFLEKIRSVPVGRALSLPSFRALQRSWYDSF
EV-F1 IL/alpaca (KC748420)	FVKLTWDNVTFLKRSFVKDERFPFLIHPVMKMSDIHESIRWTKDAKSTQDHVRSLCLLAWHCGQEQYEEFLEKIRSVPVGRALSLPSFKALQRSWYDSF
EV-F2 BHM26 (HQ917060)	FVKLTWDNVTFLKRSFVRDERFPFLVHPVMKMSDIHESIRWTKDAKSTQDHVRSLCLLAWHCGQSEYEEFLEKIRSVPVGRALSLPSFKALQRSWYDSF
EV-F2 BJ001 (HQ663846)	FVKLTWDNVTFLKRSFVRDERFPFLVHPVMKMSDIHESIRWTKDAKSTQDHVRSLCLLAWHCGQEQYEEFLEKIRSVPVGRALSLPSYKSLQRSWYDSF
EV-F2 PS89 (DQ092795)	FVKLTWDNVTFLKRSFVKDERFPFLIHPVMKMSDIHESIRWTKDAKSTQDHVRSLCLLAWHCGQEQYEEFLEKIRSVPVGRALSLPSFKALQRSWYDSF
EV-F2 3A (AY508697)	FVKLTWDNVTFLKRSFVKDERFPFLIHPVMKMSDIHESIRWTKDAKSTQDHVRSLCLLAWHCGQEQYEEFLEKIRSVPVGRALSLPSFKALQRSWYDSF
Ho12/Bos taurus/JPN/2014	FVKLTWDNVTFLKRSFVKDERFPFLIHPVMKMSDIHESIRWTKDAKSTQDHVRSLCLLAWHCGQEQYEEFLEKIRSVPVGRALSLPSFKALQRSWYDSF
IS2/Bos taurus/JPN/1990	FVKLTWDNVTFLKRSFVKDERFPFLIHPVMKMSDIHESIRWTKDAKSTQDHVRSLCLLAWHCGQEQYEEFLEKIRSVPVGRALSLPSFKALQRSWYDSF
EV-F3 PS87/Belfast (DQ092794)	FVKLTWDNVTFLKRSFVKDERFPFLIHPVMKMSDIHESIRWTKDAKSTQDHVRSLCLLAWHCGQEQYEEFLEKIRSVPVGRALSLPSFKALQRSWYDSF
EV-F4 Possum enterovirus W1 (AY462106)	FVKLTWDNVTFLKRSFVKDERFPFLIHPAMKMTDIHESIRWTKDAKSTQDHVRSLCLLAWHCGEKQYEEFLEKIRSVPVGRALSLPSFKALQRTWYDSF
EV-F4 Possum enterovirus W6 (AY462107)	FVKLNWDNVTFLKRSFVKDRRFPFLIHPAMKMSDIHESIRWTKDAKSTQDHVRSLCLLAWHCGQDQYEEFLEKIRSVPVGRALSLPSFKALQRSWYDSF
EV-A (NC 001612)	FNEVNWGNATFLKRGFLPDEQFPFLIHPTMPMKEIHESIRWTKDARNTQDHVRSLCLLAWHNGKQEYEKFVSTIRSVPVGKALAIPNYENLRRNWLELF
EV-B (NC 001472)	FNEVTWANVTFLKRYFRADEQYPFLVHPVMPMKDIHESIRWTKDPKNTQDHVRSLCLLAWHNGEHEYEEFIRKIRSVPVGACLTLPAFSTLRRKWLDSF
EV-C (NC 002058)	FETVTWENVTFLKRFFRADEKYPFLIHPVMPMKEIHESIRWTKDPRNTQDHVRSLCLLAWHNGEEEYNKFLAKIRSVPIGRALLLPEYSTLYRRWLDSF
EV-D (NC 001430)	FTETTWENVTFLKRYFRADEQYPFLIHPVMPMKEIHESIRWTKDPRNTQDHVRSLCLLAWHNGEETYNEFCRKIRTVPVGRALALPVYSSLRRKWLDSF
EV-G (NC 004441)	YVDLDWTNVTFLKRNFVPDEKYPFLVHPVMPMKEIYESIRWTRDARNTQDHVRSLCLLAWHNGRKEYEEFCRKIRSVPVGRALHLPSYSSLLREWYEKF
EV-H (NC 003988)	FTDVTWENVTFLKRYFRSDERFPFLIHPVMPMSEIFESIRWTRNAKHTQEHVTSLCYLAWHNGEEKYNDFLKRIRSVPVGKCLNLPAFKALEREWYDKF
EV-J (NC 010415)	FNNVTWENVTFLKRYFRADEQYPFLVHPVMPMKEIHESIRWTKDAKNTQEHVRSLCELAWHNGQEVYEEFLSKIRSVPVGRCLLLPAYTTLRRKWLDQF
RV-A (NC 001617)	FQELTYKNVTFLKRGFRADERHSFLIHPTFPVAEIHDSIRWTKNPSCMQEHVLSLCHLMWHNGRHAYQEFIKGIRSVSAGRALYIPAYEVLEHEWYEKF
RV-B (NC 001490)	FTKMTWENLTFLKRYFKPDQQFPFLVHPVMPMKDIHESIRWTKDPKNTQDHVRSLCMLAWHSGEKEYNEFIQKIRTTDIGKCLILPEYSVLRRRWLDLF
RV-C (NC 009996)	FSKMTWDNVTFLKRKFKPDSKYKFLIHPVYSMQDVYESIRWTKDPKNTQDHVHSLCLLAWHNGEEVYEDFCAKIRTTSVGKSLYLPPYKYLYRQWIDLF
